# 3D‐Printed Polymeric Biomaterials for Health Applications

**DOI:** 10.1002/adhm.202402571

**Published:** 2024-11-05

**Authors:** Yuxiang Zhu, Shenghan Guo, Dharneedar Ravichandran, Arunachalam Ramanathan, M. Taylor Sobczak, Alaina F. Sacco, Dhanush Patil, Sri Vaishnavi Thummalapalli, Tiffany V. Pulido, Jessica N. Lancaster, Johnny Yi, Jeffrey L. Cornella, David G. Lott, Xiangfan Chen, Xuan Mei, Yu Shrike Zhang, Linbing Wang, Xianqiao Wang, Yiping Zhao, Mohammad K. Hassan, Lindsay B. Chambers, Taylor G. Theobald, Sui Yang, Liang Liang, Kenan Song

**Affiliations:** ^1^ Manufacturing Engineering, The School of Manufacturing Systems and Networks (MSN), Ira A. Fulton Schools of Engineering Arizona State University (ASU) Mesa AZ 85212 USA; ^2^ School of Environmental, Civil, Agricultural, and Mechanical Engineering (ECAM), College of Engineering University of Georgia Athens GA 30602 USA; ^3^ School of Chemical, Materials and Biomedical Engineering (CMBE), College of Engineering University of Georgia Athens GA 30602 USA; ^4^ Department of Immunology Mayo Clinic Arizona 13400 E Shea Blvd Scottsdale AZ 85259 USA; ^5^ Department of Medical and Surgical Gynecology Mayo Clinic Arizona 5777 E Mayo Blvd Phoenix AZ 85054 USA; ^6^ Division of Laryngology, Department of Otolaryngology Mayo Clinic Arizona Phoenix AZ USA; ^7^ Division of Engineering in Medicine, Department of Medicine, Brigham and Women's Hospital Harvard Medical School Cambridge MA 02139 USA; ^8^ Physics, Franklin College of Arts and Sciences University of Georgia Athens GA 30602 USA; ^9^ Center for Advanced Materials Qatar University Doha 2713 Qatar; ^10^ Materials Science and Engineering, School for Engineering of Matter Transport and Energy (SEMTE) at Arizona State University Tempe AZ 85287 USA; ^11^ 123DTechs Inc Athens GA 30602 USA

**Keywords:** advanced manufacturing, biomedical, healthcare, pharmaceutical, regenerative medicine

## Abstract

3D printing, also known as additive manufacturing, holds immense potential for rapid prototyping and customized production of functional health‐related devices. With advancements in polymer chemistry and biomedical engineering, polymeric biomaterials have become integral to 3D‐printed biomedical applications. However, there still exists a bottleneck in the compatibility of polymeric biomaterials with different 3D printing methods, as well as intrinsic challenges such as limited printing resolution and rates. Therefore, this review aims to introduce the current state‐of‐the‐art in 3D‐printed functional polymeric health‐related devices. It begins with an overview of the landscape of 3D printing techniques, followed by an examination of commonly used polymeric biomaterials. Subsequently, examples of 3D‐printed biomedical devices are provided and classified into categories such as biosensors, bioactuators, soft robotics, energy storage systems, self‐powered devices, and data science in bioplotting. The emphasis is on exploring the current capabilities of 3D printing in manufacturing polymeric biomaterials into desired geometries that facilitate device functionality and studying the reasons for material choice. Finally, an outlook with challenges and possible improvements in the near future is presented, projecting the contribution of general 3D printing and polymeric biomaterials in the field of healthcare.

## Graphical abstract

This review explores the potential of 3D printing for creating functional polymeric health‐related devices, examining current techniques and commonly used biomaterials. It highlights key applications in biosensors, bioactuators, and soft robotics, while addressing challenges like material compatibility, printing resolution, and speed. The review concludes with future outlooks for 3D‐printed biomaterials in healthcare advancements.

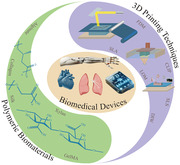

## 1 Introduction

**Figure 1:**
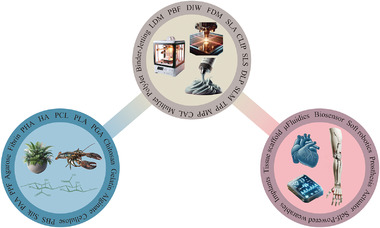
Overview of 3D printing, biopolymers, and their interactions for human health and medical applications.

As life expectancy has consistently increased over the past century,^[^ ^1^ ^]^ the quality of life has drawn a greater concern. Biosensors, actuators, wearable robotics, advanced energy storage systems, and self‐powered devices are enabling tools for improving the quality of life. For instance, biosensors continuously collect and monitor wearers’ bio‐signals under normal life conditions,^[^ ^2^ ^]^ which is essential for analyzing the health status of individuals, especially the elderly, athletes, and people with chronic conditions.^[^ ^3^ ^]^ Recent advances in materials science, electrical engineering, biochemistry, and microelectronics have rapidly promoted the applications of these techniques in health‐related applications.^[^ ^4^ ^]^ It is estimated that the market for point‐of‐care biosensors will be worth ≈$33 billion by 2027.^[^ ^5^ ^]^ Yet, there is still potential for improvement regarding miniaturization, biocompatibility, robustness, and lightweight. The utilization of 3D printing and biocompatible polymers constitutes two pivotal approaches for addressing the aforementioned challenges (**Figure** **1**). Conventional manufacturing of biomedical gadgets involves various methods, including but not limited to surface micromachining,^[^ ^6^ ^]^ soft lithography,^[^ ^7^ ^]^ fusion splicing,^[^ ^8^ ^]^ microcontact printing,^[^ ^9^ ^]^ and screen printing,^[^ ^10^, ^11^ ^]^ among others.^[^ ^12^, ^13^, ^14^ ^]^ As compared to these traditional manufacturing methods, 3D printing possesses advantages such as reduced material waste, enhanced flexibility in geometric design, customizability, and localized production,^[^ ^15^ ^]^ which provides immense potential for the promotion of individualized biomedical devices. On the other hand, the swift advancements in biomaterials have prompted exploration into controllable degradation, tissue regeneration, and immune response.^[^ ^16^, ^17^ ^]^

In recent years, there have been reviews on biomaterials that cover the historical development,^[^ ^18^ ^]^ a broad range of biomaterials,^[^ ^19^ ^]^ or focus on a subdivided material type.^[^ ^20^ ^]^ For example, to bridge the general knowledge of 3D printing^[^ ^21^ ^]^ with biomaterials, Sing et al. reviewed metallic biomaterials that are often processed with powder bed fusion (PBF) and directed energy deposition (DED).^[^ ^22^ ^]^ Nouri et al. focused on load‐bearing biomaterials that are manufactured by laser powder bed fusion (L–PBF).^[^ ^23^ ^]^ Yu et al. assessed specifically photopolymerizable biomaterials and the light‐based 3D printing strategies.^[^ ^24^ ^]^ In addition, the fundamental about the processibility of polymeric biomaterials for 3D printing was discussed by Pugliese et al.^[^ ^25^ ^]^

The exploration of polymeric biomaterials and 3D printing techniques in this review underscores the pivotal role of these technologies in transforming healthcare, particularly in the realm of personalized medicine. One of the core strengths of additive manufacturing lies in its capacity to fabricate highly customizable devices that cater to the unique anatomical and functional needs of individual patients. This capability is crucial in applications such as regenerative medicine, where 3D‐printed tissue scaffolds mimic the natural extracellular matrix (ECM), promoting tissue growth and healing. For example, scaffolds made from biocompatible polymers such as PCL and PLA have been successfully used in bone tissue engineering, providing both structural support and a conducive environment for cellular proliferation and differentiation. Moreover, 3D‐printed drug delivery systems have emerged as an innovative approach to address chronic inflammation and localized treatment in diseases such as cancer and musculoskeletal disorders. These systems can be designed with controlled degradation rates, ensuring precise release kinetics of therapeutic agents such as anti‐inflammatory drugs or growth factors, enhancing the efficacy of the treatment, and reducing the need for invasive procedures.

In addition to tissue engineering and drug delivery, 3D printing has shown tremendous potential in the development of bioactuators and soft robotics for wearable health devices, which will also be reviewed in this contribution. Soft robotics, fabricated with flexible polymers such as thermoplastic polyurethane (TPU), can be seamlessly integrated into wearable devices to monitor physiological parameters like heart rate, respiratory function, or muscle activity. These devices, enabled by 3D printing, offer a high degree of customization, allowing for the design of soft and stretchable materials that conform to the wearer's body, improving user comfort and accuracy of the collected data. For instance, in applications such as pelvic organ prolapse (POP) treatment, customized 3D‐printed implants can be designed to provide mechanical support while also incorporating biocompatible drug reservoirs for localized treatment of inflammation. As these devices evolve, the integration of biosensors with 3D‐printed polymers could allow for real‐time health monitoring, further enhancing the personalized care experience. The future of healthcare may see more innovative solutions where 3D printing bridges the gap between traditional medical devices and highly individualized, patient‐centered treatments, significantly impacting the management and treatment of chronic diseases and injury rehabilitation.

## 2 3D Printing

### 2.1 Fused Deposition Modeling (FDM)

**Figure 2:**
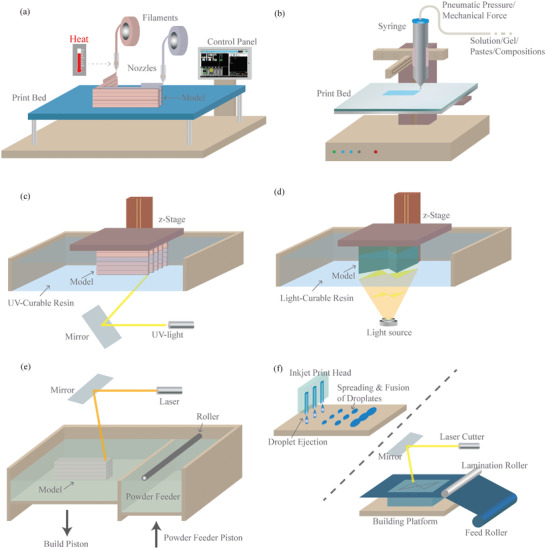
Frequently used 3D printing methods for health applications: a) FDM, b) DIW, c) SLA, d) DLP, e) PBF, f) Inkjet printing/LOM.

FDM is a prominent additive manufacturing technique that has revolutionized various industries by enabling the creation of intricate 3D objects.^[^ ^26^ ^]^ This method, also known as fused filament fabrication (FFF), operates on the principle of layer‐by‐layer deposition of material, making it a versatile and widely adopted 3D printing process. FDM involves the precise extrusion of a thermoplastic filament through a heated nozzle, which then solidifies upon cooling, building up layer upon layer to construct a complete object (**Figure** **2**a). Thus, FDM offers not only speed and cost‐effectiveness but also exceptional design flexibility, making it a preferred choice across industries.

In FDM 3D printing, several crucial parameters dictate the quality, accuracy, and mechanical properties of the printed objects. These parameters include factors such as layer height, nozzle temperature, print speed, infill density, and material choice.^[^ ^27^ ^]^ The layer height defines the thickness of each printed layer, affecting the object's surface finish. Nozzle temperature ensures proper material flow and adhesion between layers. Print speed directly influences the time taken for printing, which must be balanced with accuracy. Infill density determines the internal structure of the printed object, impacting its strength‐to‐weight ratio. Careful calibration and optimization of these parameters are imperative for achieving the desired output, be it intricate prototypes or functional parts. While FDM has garnered widespread popularity, it is not devoid of challenges. Achieving high precision and intricate detailing can be challenging due to factors such as layer adhesion, warping, and dimensional accuracy. Poor layer adhesion may lead to structural weaknesses, affecting the overall integrity of the object.^[^ ^28^ ^]^ Warping, caused by uneven cooling of layers, can distort the final product's dimensions and compromise accuracy. Moreover, achieving fine surface finishes can be challenging, often requires post‐processing techniques. Additionally, material selection plays a pivotal role, with material properties influencing the mechanical, thermal, and even biocompatibility aspects of the printed objects.

The applications of FDM span a vast array of fields, particularly in the biological, biomedical, pharmaceutical, and medical domains. In the biological sector, FDM is employed to create custom laboratory equipment, such as specialized reaction vessels and microfluidic devices.^[^ ^29^ ^]^ In the biomedical field, it finds use in the fabrication of patient‐specific implants, prosthetics, and anatomical models for surgical planning and medical training.^[^ ^30^ ^]^ In pharmaceuticals, FDM assists in the production of drug delivery systems, offering precise control over release rates and dosage forms.^[^ ^31^ ^]^ The medical arena benefits from FDM‐produced surgical tools, assistive devices, and even tissue‐engineering scaffolds that aid in regenerative medicine.^[^ ^32^ ^]^

The exceptional material diversity of FDM makes it conducive to creating prototypes, functional parts, and tools with intricate designs, ultimately advancing progress in these critical sectors. Several biopolymers commonly used in FDM include polylactic acid (PLA), ε‐polycaprolactone (PCL), polyhydroxyalkanoates (PHAs), polyacrylic acids (PAAs), poly (butylene succinate) (PBS), poly (propylene fumarate) (PPF)and poly(ethylene glycol) (PEG). For example, PLA is known for its ease of use and biodegradability, while PCL offers flexibility and slow biodegradation. PHAs and PEG, known for their biocompatibility and biodegradability, may be enhanced by cellulose for mechanical durability during degradation. The printing mechanism of FDM dictates the use of thermoplastic materials characterized by robust thermal stability, such as the aforementioned synthetic biocompatible polymers.

### 2.2 Direct Ink Writing (DIW)

DIW has emerged as a powerful additive manufacturing technique that enables precise deposition of materials in a controlled manner, offering remarkable versatility and potential for various applications.^[^ ^33^ ^]^ DIW, also known as robocasting or extrusion‐based 3D printing, involves the layer‐by‐layer deposition of viscoelastic inks or pastes, creating intricate three‐dimensional structures (Figure 2b). This method allows for the fabrication of objects with complex geometries and tailored material compositions, making it well‐suited for creating functional components across industries.

In DIW 3D printing, a multitude of parameters govern the final quality and properties of the printed structures. These parameters include ink composition, printing speed, nozzle size, layer height, and printing temperature.^[^ ^34^ ^]^ The ink composition directly influences the material's rheological properties and its ability to flow through the nozzle. Printing speed and nozzle size determine the precision and resolution of the printed object. Layer height impacts the vertical resolution, while printing temperature affects material viscosity and adhesion between layers. Optimizing these parameters is crucial for achieving the desired mechanical, electrical, and functional properties of the printed objects. Despite its capabilities, DIW 3D printing presents certain challenges. Achieving high resolution and intricate detailing can be complex due to factors such as nozzle clogging, material extrusion consistency, and print stability.^[^ ^35^ ^]^ Maintaining a consistent ink flow is essential for uniform layer deposition and structural integrity. Additionally, controlling the shape and stability of printed objects during the deposition process is crucial to prevent deformation or collapse. Moreover, post‐processing steps, such as curing or drying, can affect the final properties of the printed structures. Addressing these challenges requires a deep understanding of material behavior, ink formulation, and process parameters.

DIW 3D printing finds a multitude of applications in the development of advanced functional systems. In the realm of sensors, DIW is employed to create custom‐designed sensing structures with intricate geometries and precise material compositions.^[^ ^36^ ^]^ Actuators benefit from DIW by allowing the fabrication of complex, multi‐material structures capable of controlled motion.^[^ ^37^ ^]^ Soft robotics leverage DIW to create flexible, deformable components that mimic biological systems, enabling intricate and versatile robotic designs. Beyond this, DIW plays a pivotal role in the creation of smart systems, including wearable devices,^[^ ^38^ ^]^ energy‐harvesting components,^[^ ^39^ ^]^ and microfluidic platforms.^[^ ^40^ ^]^ The adaptability of DIW to various materials, including conductive and piezoelectric ones, positions it as a key enabler in developing intelligent systems with enhanced functionalities and performance.

DIW 3D printing utilizes a range of biopolymers similar to those in FDM, each with distinct biocompatibility and biodegradability characteristics. For instance, alginate, derived from seaweed,^[^ ^41^ ^]^ forms biocompatible hydrogels and is employed in tissue scaffolds and cell encapsulation.^[^ ^42^, ^43^ ^]^ Similarly, chitosan, obtained from crustacean shells, supports cell adhesion and tissue regeneration in applications such as wound healing and drug delivery. Hyaluronic acid (HA), a natural component of the extracellular matrix (ECM), is used in regenerative medicine and tissue engineering for its biocompatibility. Collagen, a major structural protein, offers a tissue‐like environment for cartilage repair and skin grafts. Gelatin, derived from collagen, promotes cell growth and is applied in tissue engineering and drug delivery. Fibrin, crucial in blood clotting, is used to bioprint vascularized tissue constructs. Xylan, a plant‐derived polysaccharide, can be incorporated into DIW as a biodegradable and environmentally friendly product, particularly in the realm of packaging and disposable items. Agarose, sourced from seaweed, is used in DIW for bioprinting applications. It serves as a support material for the creation of complex structures like vascular networks within tissue constructs. After printing, agarose can be dissolved, leaving behind hollow channels for blood flow or nutrient transport in engineered tissues. Carrageenan, another seaweed‐derived biopolymer, is utilized in DIW for fabricating edible and biodegradable objects in food‐related 3D printing, where it can be used to create custom‐shaped food products and eco‐friendly food packaging. Additionally, silk fibroin, sourced from silkworms, provides mechanical support for bone and cartilage regeneration. Compared to FDM, the benign process conditions of DIW allow more vulnerable materials to serve as the feedstock. Some of these materials are nature‐derived and resemble the structure of ECM. These biopolymers collectively enable the creation of biocompatible and biodegradable structures via DIW 3D printing for diverse biomedical applications. Furthermore, with the capability to incorporate cells and growth factors, DIW offers additional avenues for regenerative medicine and tissue engineering.

### 2.3 Stereolithography (SLA)

SLA is a groundbreaking additive manufacturing technique that has redefined how intricate three‐dimensional objects are created. This method is recognized for its high precision and ability to produce complex geometries with exceptional surface finish.^[^ ^44^ ^]^ SLA operates through the precise point‐by‐point and then layer‐by‐layer curing of liquid photopolymer resins mostly using ultraviolet (UV) light. The resin solidifies when exposed to UV light, gradually forming the final object (Figure 2c). Renowned for its accuracy and ability to create intricate details, SLA 3D printing is widely employed in industries requiring precision, such as jewelry design, aerospace, and medical devices.^[^ ^45^ ^]^

Numerous parameters influence the quality and characteristics of SLA 3D‐printed objects. These parameters include layer thickness, exposure time, resin formulation, and printing speed.^[^ ^46^ ^]^ Layer thickness determines the vertical resolution of the printed object, with thinner layers yielding finer details. Exposure time controls the degree of resin solidification, impacting the strength and mechanical properties of the final part. The choice of resin, whether standard, flexible, or specialized, determines material properties such as stiffness, durability, and transparency. Adjusting these parameters requires a fine balance to achieve the desired outcome, be it an intricately detailed prototype or a functional part. Despite its advantages, SLA 3D printing presents certain challenges.^[^ ^47^ ^]^ Achieving consistent curing and layer adhesion is critical for structural integrity. Inadequate curing or incomplete layer bonding can lead to weak spots or reduced mechanical strength. Furthermore, resin shrinkage during the curing process can cause distortion and affect dimensional accuracy. Post‐processing steps, such as cleaning and curing, are essential but can be time‐consuming. The materials used in SLA, particularly the photopolymer resins, may have limited mechanical properties compared to traditional manufacturing materials. Addressing these challenges requires careful consideration of resin selection, exposure settings, and post‐processing techniques.

SLA 3D printing boasts a diverse range of applications, particularly in crafting functional systems across various fields. For instance, SLA‐printed electronics include complete electrical structures,^[^ ^48^ ^]^ conductive structures,^[^ ^49^ ^]^ sensors,^[^ ^50^ ^]^ antennas,^[^ ^51^ ^]^ waveguide,^[^ ^52^ ^]^ passive components,^[^ ^53^ ^]^ and dielectric structures.^[^ ^54^ ^]^ In the realm of thermal systems, SLA assists in creating intricate cooling structures for electronics and advanced heat exchangers.^[^ ^55^ ^]^ Wearable electronics benefit from SLA's precision in producing custom‐designed enclosures and flexible components. Optical applications leverage the ability to create intricate lens shapes and transparent parts with excellent surface quality. Besides high resolution and good surface quality, negligible porosity and isotropy are also factors that make SLA suitable for producing optical components.^[^ ^56^ ^]^ Compatible transparent feedstocks can be synthesized^[^ ^57^ ^]^ or obtained commercially,^[^ ^58^ ^]^ and post‐treatment can be considered to further enhance the optical properties.^[^ ^57^, ^58^ ^]^ Acoustic systems benefit from SLA by enabling the fabrication of precisely tuned sound waveguides and resonators.^[^ ^59^ ^]^ Additionally, SLA plays a pivotal role in crafting functional prototypes, intricate models for architectural design, patient‐specific medical devices, and other biomedical applications such as implantable devices,^[^ ^60^ ^]^ tissue engineering,^[^ ^61^ ^]^ and cell‐containing hydrogels.^[^ ^62^ ^]^ The adaptability of SLA to a wide range of materials and the exceptional surface finish it offers make it a valuable tool in developing advanced functional systems across diverse industries.

SLA 3D printing primarily utilizes photosensitive resins rather than traditional biopolymers due to the photopolymerization process involved. These resins, when exposed to light, undergo a chemical reaction that causes them to solidify and form 3D structures. For example, PAAs, PCL, PEG, PPF, chitosan, gelatin, HA, and alginate can be UV‐curable after chemical modification and are compatible with SLA (**Table** **1**). Some formulations of SLA resins can be modified or combined with biocompatible materials to create bioresins suitable for specific medical and dental applications.^[^ ^63^ ^]^ These bioresins may incorporate biopolymers or materials with biocompatible properties, but they are typically not easily degradable biopolymers. Examples of such biocompatible bioresins used in SLA 3D printing include dental resins for creating dental prosthetics and medical‐grade resins for anatomical models and surgical guides. These resins are designed to meet regulatory standards for patient safety and biocompatibility.

**Table 1:** 3D printing involving particle processing.

Printing mechanism	3D Printing	Material compatibility	Typical printing resolution		Manufacturing features	
			*xy* [µm]	*z* [µm]	Strengths	Weaknesses
Vat polymerization‐based	General SLA	Monomers	10–200	20–300	Fine resolution, smooth surface, minimal wrapping, transparency	Limited to UV‐curable resins, slow processing
	DLP	Monomers and some polymers (e.g., PEG and PVA)	30–200	20–150	High speeds, relatively good surface quality, transparency	Limited resolutions, existence of defects, costly, photopolymers only
Jetting‐based	Direct InkJet	Monomers	10–150	10–50	Multimaterial & multi‐nozzle design, high resolution	Limited low viscosity, limited mechanical properties
	EHD Jet	Small molecules of PCL, PEO, PEDOT: PSS, PLA, PVA.	0.03–50	1	Ultra‐high resolution, wide materials choice	Slow printing, no commercial models, coffee ring effect, complex setup
	Binder Jetting	Monomers and resins as binders	50	50–200	Low‐viscosity, flexible with powder types, cost‐effective for large parts	Low resolution, post‐processing required, low mechanics, high defects
Extrusion‐based	LDM/ DIW	PEG, PEGDA, PLA, PLG, PLLA, PLGA, PU, PVA	50–500	100–400	Compatibility with solutions and gels, easy mechanical enhancement, affordable, customizable nozzles	Limited resolution & material diffusion or relaxation during printing, low overall mechanical properties
	FDM/ FFF	PETG, PLA, PU, PVA, TPU	20–200	10–200	Broad material choices, low cost, simple setup, easy enhancement in multiple properties	Anisotropy, poor layer adhesion, nozzle clogging, defects (e.g., voids and cracks, poor surface finish)

### 2.4 Digital Light Processing (DLP)

DLP 3D printing is a cutting‐edge additive manufacturing technique that has gained significant attention for its high speed and precision. This method utilizes planar digital light projection to solidify liquid photopolymer resins directly layer‐by‐layer, forming intricate 3D objects.^[^ ^64^ ^]^ In DLP, a light source projects patterns onto the resin, causing it to selectively cure and harden (Figure 2d). The ability to achieve fine details and a smoother surface finish makes DLP a preferred choice for applications demanding intricate geometries and high‐resolution prints. Both DLP and SLA are resin‐based 3D printing technologies that use light to cure liquid photopolymer resin into solid objects. However, they differ in their light sources and printing methods. DLP employs a digital micromirror device (DMD) or a liquid crystal display (LCD) to project a whole layer of the object onto the resin simultaneously, allowing for faster printing speeds as each entire layer cures at once.^[^ ^65^ ^]^ The DMD is composed of an array of tiny mirrors, each of which has “on” and “off” states that determine whether it reflects light or not.^[^ ^64^ ^]^ As a result, the resolution of the generated pixelated light fields and the selective curing sites in the resin tank were limited by the resolution of the DMD. In contrast, SLA uses a laser to selectively solidify one point or a small area at a time, leading to potentially slower print times but often offering higher precision and finer details. As a result, DLP can be more cost‐effective for large‐scale, rapid production, while SLA tends to excel in intricate, highly detailed applications such as jewelry, dental prosthetics, and engineering prototypes. Both DLP and SLA fall under the category of vat polymerization, and each method has its advantages and is chosen based on the specific requirements of the project.

Various parameters play a pivotal role in achieving optimal results in DLP 3D printing. Parameters such as layer thickness, exposure time, resin composition, and light intensity influence the final quality of the printed object. Layer thickness affects vertical resolution, while exposure time determines how well the resin solidifies under the light. The resin's composition dictates mechanical properties, such as stiffness and flexibility. Moreover, light intensity impacts curing depth and the rate of photopolymerization. Precise calibration and optimization of these parameters are crucial to creating objects with the desired level of accuracy and mechanical integrity. On the positive side, DLP 3D printing offers numerous advantages, such as faster printing speed, higher resolution, and smoother surface finish; however, it also presents certain challenges.^[^ ^66^ ^]^ Achieving consistent curing throughout the printed object can be complex, leading to potential defects or uneven mechanical properties. Post‐processing, such as cleaning and curing, is essential but can be time‐consuming. Material considerations are crucial, as photopolymer resins may have limitations in terms of mechanical strength and resistance to environmental factors. Additionally, as with many AM techniques, scaling up to larger prints without sacrificing quality can be challenging.

DLP 3D printing has made significant strides in the realm of energy systems. This technology enables the creation of intricate structures for energy storage devices such as batteries and fuel cells.^[^ ^67^ ^]^ The precision and fine details achievable with DLP play a role in optimizing electrode designs and enhancing energy efficiency. For thermoelectronics, DLP aids in fabricating thermoelectric materials with customized geometries to efficiently convert heat into electricity.^[^ ^68^ ^]^ Optoelectronic components also benefit from DLP, with the ability to print intricate light‐guiding structures for optical devices. In the realm of biomedical applications, DLP plays a role in creating physical models and personalized external devices,^[^ ^69^ ^]^ medical implants,^[^ ^70^ ^]^ drug delivery systems,^[^ ^71^ ^]^ and tissue engineering constructs.^[^ ^72^ ^]^ DLP's capacity to create highly detailed and complex structures positions it as a valuable tool in advancing energy systems' performance and functionality across a range of applications.

Similar to SLA, in the context of DLP 3D printing, the utilization of biopolymers is limited, primarily due to challenges in their photo‐curability. Biopolymers such as PAAs,^[^ ^73^ ^]^ PEG,^[^ ^74^ ^]^ PPF,^[^ ^75^ ^]^ gelatin,^[^ ^76^ ^]^ and silk fibroin^[^ ^77^ ^]^ are reported to serve as the ink for DLP after proper functionalization. On the other hand, the technology does allow for the use of blends of photopolymerizable compounds and biocompatible additives. These specially formulated bioresins are tailored to meet strict regulatory standards for biocompatibility and safety, making them suitable for various medical and dental applications. One notable category of bioresins includes those developed for dental applications.^[^ ^78^ ^]^ These dental resins are engineered to be safe for intraoral use, making them ideal for the production of dental prosthetics like crowns, bridges, and dentures. Additionally, medical‐grade resins are employed for creating anatomical models and surgical guides, adhering to stringent biocompatibility requirements when these printed objects come into contact with the human body. Furthermore, specific biocompatible photopolymers have been designed to cater to emerging fields like bioprinting and tissue engineering. These materials are meticulously crafted to ensure compatibility with biological cells and tissues,^[^ ^79^ ^]^ enabling the creation of intricate and biocompatible structures using DLP 3D printing technology. The selection of bioresins or biocompatible materials depends on the intended application and required biocompatibility and safety for the 3D‐printed objects.

### 2.5 Selective Laser Sintering (SLS)/Selective Laser Melting (SLM)

SLS and SLM are advanced additive manufacturing techniques that utilize laser technology to create complex three‐dimensional objects, both belonging to the PBF category. In SLS, powdered materials are selectively fused together layer by layer using a high‐power laser,^[^ ^80^ ^]^ while in SLM, metallic powders are melted and fused to form solid structures (Figure 2e).^[^ ^81^ ^]^ These methods enable the fabrication of intricate and functional parts by sintering or melting the material powder based on a digital model. Both SLS and SLM offer high precision, design flexibility, and the capability to work with a wide range of materials, including plastics, metals, and ceramics.

A multitude of parameters impact the outcome of SLS and SLM 3D printing processes. Layer thickness, laser power, scanning speed, and material properties significantly influence the final part's mechanical properties and surface finish.^[^ ^82^ ^]^ Layer thickness defines the vertical resolution, while laser power determines the energy input for fusion or sintering. Scanning speed affects the speed of the process and the material's exposure to the laser. Material properties, including powder size and composition, play a crucial role in achieving the desired material behavior during the laser interaction. The optimization of these parameters is essential to ensure the quality, integrity, and accuracy of the printed parts. In spite of their remarkable advantages, both SLS and SLM also present unique challenges.^[^ ^83^ ^]^ Achieving uniform energy distribution to ensure consistent sintering/melting throughout the printed object is a complex task. The thermal properties of materials impact energy absorption and dissipation, leading to potential defects or variations in mechanical properties. Powder handling and recycling require careful consideration to maintain the quality and consistency of the printed material. In SLM, issues like residual stress, microcracks, and warping can arise due to the rapid heating and cooling during the melting process. Additionally, post‐processing steps such as support removal and heat treatment may be necessary to achieve the final desired properties.

SLS and SLM 3D printing have gained significant traction in the development of self‐powered systems. These techniques offer the ability to create intricate structures and functional components for energy‐harvesting devices. Examples of applications include piezoelectric generators that convert mechanical vibrations into electricity,^[^ ^84^ ^]^ thermoelectric modules for harvesting waste heat,^[^ ^85^ ^]^ and flexible solar cell components integrated into wearable electronics.^[^ ^86^ ^]^ These energy‐related materials usually consist of ceramics, metals, and conjugated polymers, which are relatively difficult to process without binders or solvents when using other 3D printing methods. While the inclusion of additional materials might affect electrical properties, such as capacity and conductivity, PBF can yield higher functional material content and better grain boundaries, potentially benefiting the performance of self‐powered systems. Furthermore, SLS and SLM enable the precise fabrication of complex geometries necessary for efficient energy conversion and collection. These techniques empower the creation of self‐powered systems that contribute to sustainable and autonomous technologies in industries, such as electronics, aerospace, and healthcare.

SLS 3D printing employs a variety of biopolymers to cater to different applications and requirements. These biopolymers are favored for their biocompatibility, biodegradability, and versatility in additive manufacturing. Among the commonly used options are PLA and PCL, known for their biodegradability and ease of printing. PCL is valued for its flexibility and slow biodegradation, making it suitable for long‐lasting applications. PBS, derived from succinic acid and 1,4‐butanediol, is a biodegradable polyester with potential applications in packaging, agriculture, and biomedical area. PEG, compatible with other 3D printing techniques as well, is often used in the formulation of drug delivery systems and as a coating on medical devices to reduce immunogenicity. PGA belongs to the family of aliphatic polyesters, in the same class as PLA. It plays an important role in medical sutures, tissue engineering, and drug delivery. PHAs, a type of biopolyester produced by certain bacteria, are biodegradable in various environments and can be applied in packaging materials, agricultural films, and medical devices. Collagen contributes to tissue engineering with their biocompatible and tissue‐mimicking properties. Starch‐based biopolymers cater to sustainable, biodegradable products. These biopolymers collectively provide a versatile toolkit for creating functional, biocompatible, and environmentally friendly 3D‐printed objects, spanning from medical devices to sustainable packaging materials.

As compared, SLM 3D printing primarily utilizes metal powders and ceramics for precision engineering applications, rather than traditional biopolymers. The SLM process involves the precise melting and solidification of these materials using a high‐energy laser. While biopolymers are not commonly used in the SLM process due to their limited heat resistance and thermoplastic nature, the technology does find applications in the medical field, particularly in the production of customized orthopedic implants and dental prosthetics. These implants are typically composed of biocompatible materials such as titanium alloys or cobalt‐chromium, chosen for their strength, durability, and ability to integrate seamlessly with the human body.

### 2.6 Other 3D Printing Mechanisms

In addition to well‐established 3D printing methods like FDM, DIW, SLA, DLP, and SLS/SLM, there are several other innovative 3D printing methods that offer unique capabilities and applications in diverse fields. These methods include inkjet (e.g., MultiJet and PolyJet), binder jetting, Laminated Object Manufacturing (LOM), and Electron Beam Melting (EBM) (Figure 2f). Among them, LOM employs layers of adhesive‐coated paper, plastic, or metal sheets.^[^ ^87^ ^]^ These sheets are cut and bonded together using heat or pressure to create intricate objects. Thus, the use of biopolymers in LOM is relatively uncommon due to the specific requirements of the process, which relies on sheets that can be easily cut and bonded by lasers or other cutting mechanisms. EBM uses an electron beam to melt metal powder layer by layer, resulting in a highly dense and structurally sound end product, often employed in aerospace and medical industries.^[^ ^88^ ^]^ This method is particularly useful for metals, ceramics, and sand‐casting molds. Thus, biopolymers are not typically used in EBM because the process requires materials with high melting points and excellent thermal stability, which most biopolymers do not possess.

Binder Jetting is a 3D printing technology that primarily utilizes powdered materials, such as ceramics, metals, and polymers,^[^ ^89^ ^]^ but it is not commonly associated with the use of traditional biopolymers. Binder Jetting relies on selectively depositing a liquid binding agent onto thin layers of powdered material to create 3D objects. As compared, Inkjet printing, a versatile additive manufacturing method, employs inkjet printheads to deposit droplets of liquid material onto a substrate, creating intricate layers that gradually build up into usually 2D or 2.5D objects.^[^ ^90^ ^]^ This technique, often referred to as “droplet‐based” printing, enables precise control over material deposition, making it suitable for applications requiring fine details and multi‐material capabilities. Inkjet printing finds applications in various industries, including electronics, biofabrication, and custom manufacturing, offering the potential to create functional components, intricate prototypes, and even complex biological constructs. In bioprinting and tissue engineering, some commonly used biopolymers or biocompatible materials for binders in binder jetting and inks in inkjet may include alginate derived from seaweed, collagen as a major structural protein in the human body, fibrin as a natural protein involved in blood clotting, and HA as a component of the ECM, as well as Matrigel, a gelatinous protein mixture derived from the Engelbreth‐Holm‐Swarm (EHS) mouse sarcoma tumor.

These alternative 3D printing methods can also offer exciting possibilities for creating objects with biocompatible materials, such as biopolymers, cells, and tissues. In the realm of biopolymers, these methods enable the fabrication of customized implants,^[^ ^91^ ^]^ drug delivery systems,^[^ ^92^ ^]^ and medical devices with tailored mechanical properties. Cellular and tissue applications include creating intricate scaffolds for tissue engineering and regenerative medicine.^[^ ^93^ ^]^ Moreover, these methods contribute to developing personalized medical devices and implants, advancing research in drug testing using 3D‐printed tissue models, and paving the way for the creation of functional organs through bioprinting. The versatility of these methods, combined with the potential of biocompatible materials, presents a new frontier in the field of additive manufacturing and biomedical applications. Also, see Table **1** of the 3D printing pros and cons as a summary.

## 3 Biopolymers for 3D Printing from the Molecular Perspective

### 3.1 Biopolymer Overview

**Figure 3:**
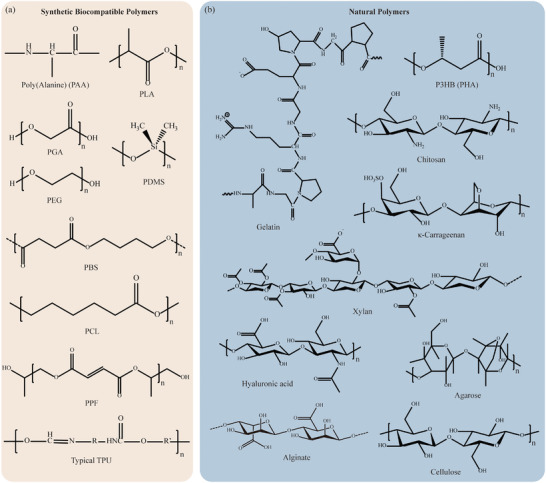
Molecular structures of common a) synthetic biocompatible polymers and b) natural polymers.

In the realm of biocompatible polymers, two broad categories exist: bioinert and biodegradable. Typical bioinert polymers include polyetheretherketone (PEEK), polypropylene (PP), thermoplastic polyurethane (TPU), polydimethylsiloxane (PDMS), polytetrafluoroethylene (PTFE), poly(methyl methacrylate) (PMMA), and polyethylene terephthalate (PET). Although non‐degradable, these polymers have been approved for the production of medical implants.^[^ ^94^, ^95^ ^]^ These commercialized synthetic polymers are considered biocompatible due to their bioinertness, while others are designed to degrade in vivo after fulfilling their function. The biodegradable polymers can be classified into two categories: synthetic biodegradable polymers and natural polymers. Synthetic polymers engineered for biodegradability have gained prominence in both environmentally conscious industries and medical fields. For example, polymers like polylactic acid (PLA), poly(glycolic acid) (PGA), and poly(ε‐caprolactone) (PCL), are designed to naturally degrade into non‐toxic byproducts over time, minimizing their environmental impact. This category of materials usually undergo ester hydrolysis in vivo since most of them are polyester, with the exception for other polymer types such as polyamino acids (PAAs) and polyethylene oxide (PEO). In the medical sector, these polymers offer the advantage of temporary implantation, eliminating the need for surgical removal. This marriage of synthetic polymers with varying biodegradability addresses sustainability concerns and aligns with the increasing demand for eco‐friendly solutions across diverse industries. Natural polymers, derived from living organisms, exhibit more similarities with the ECM in the human body, benefiting not only cell viability but also cell adhesion. Proteins, polysaccharides, and nucleic acids are widely seen as natural polymers. For instance, proteins such as collagen and gelatin, commonly found in connective tissues, serve as excellent biomaterials due to their biocompatibility. Similarly, polysaccharides like chitosan, derived from chitin, possess biocompatibility and wound‐healing properties. Alginate, derived from seaweed, forms hydrogels that can encapsulate cells for bioprinting applications. PHAs, produced by microorganisms, offer biodegradability similar to traditional polymers but with reduced environmental impact. These biomacromolecules are widely employed in tissue engineering, wound dressing, and drug delivery systems, facilitating cellular adhesion and integration within the body. In advancing additive manufacturing techniques, they play a pivotal role and contribute to the development of personalized medical implants and regenerative medicine solution. **Figure** **3** demonstrates the molecular structures of typical synthetic biocompatible polymers and natural polymers. Their properties and compatible manufacturing methods are listed in **Table** **2**. In the following paragraphs, a detailed introduction is provided for these biocompatible polymers, particularly those reported to possess biodegradability.

**Table 2:** List of typical synthetic and natural polymers for biomedical applications.

Polymers		Processing		Mechanical properties		Thermal properties			Biocompatibility		Reference
Type	Name (alphabetical order)	General manufacturing	3D printability	E [MPa]	σ [MPa]	T_g_ [°C]	T_m_ [°C]	T_c_ (°C)	Cell viability	Biodegradability (lifetime for human body environment)	
Synthetic	PAAs	Solution‐based casting, spin coating, dip coating, & melt‐based extrusion and molding	FDM, SLA, DIW, DLP	1225–2745	37–77	40–140	95–168	≈114	Good, non‐immunogenic	Various degradation rates, enzymatic degradable	[96, 97, 98, 99, 100, 101, 102]
	PBS	Extrusion, injection molding, blow molding, film casting, foaming, thermoforming	FDM, SLS, pellet extrusion 3D printer	326.3–387	34–55	−40 to −10	90–120	60–82	Good	Potentially enzymatic degradable	[103, 104, 105, 106, 107, 108]
	PCL	Fiber bonding, salt leaching, solvent casting, phase separation, injection molding, gas foaming, microsphere sintering	FDM, EHDP, DIW, SLA, SLS, DLP	240–420	16–24	∼−60	∼57	34–50	Good viability, low adhesion	Slow degradation	[109, 110, 111, 112]
	PDMS	Casting and molding, soft lithography, injection molding, spin coating, extrusion, solvent casting	DIW, DLP, SLA, PolyJet, SLS	1–3	3–5	−150 to −123	−40 to −50	←70	Hydrophobic, low adhesion	Non‐degradable	[113, 114, 115, 116, 117, 118, 119]
	PEG	Melt or solution for extrusion, solvent casting, injection molding, compression molding	FDM, SLA, DLP, DIW, SLS	0.01–0.035	0.02	−85 to −50	1–66	−6 to 42.5	Good	Varies based on polymer Mw and environments	[120, 121, 122, 123, 124, 125]
	PGA	Melt extrusion, solution processing, injection modeling, compression molding, blow molding	FDM, SLS, DIW	8400	890	34–40	222–230	∼180	Good	Rapid degradation	[126, 127, 128, 129]
	PLA	Extrusion, injection molding, injection stretch blow molding, casting, thermoforming	FDM, SLS	50–13 800	40–60	50–65	170–180	100–120	Good	Slow degradation	[130, 131, 132]
	PPF	Gas foaming, electrospinning, extrusion, injection molding, solvent casting	FDM, SLM, SLA, cDLP	195–1000	≈62	24–32			Good	Slow degradation	[133, 134, 135, 136, 137, 138, 139]
	TPU	Inject molding, extrusion, blow molding, thermofoaming, calendaring, compression molding	FDM, SLS, SLA, DLP, MJF	10–50	20–80	−73 to −23	170–230	50–120	Generally good	Non‐degradable	[140, 141, 142, 143, 144]
Natural	Agarose	Gel electrophoresis, molding	DIW	0.083–3.69	0.048–0.453	∼−30	∼90 (Gel state)	∼30 (Aqueous solution)	Inert, cells do not adhere	Generally degradable	[145, 146, 147]
	Bacterial cellulose	Compression molding, casting	DIW	15 000–35 000	200–300		120–160		Excellent	Limited	[148, 149]
	Carrageenan	Extrusion, spray drying, emulsification, gel forming, film and coating	DIW	0.0025–0.35	5.5–37.3		31–68 (Gel state)	19–43 (Aqueous solution)	Excellent	Controllable degradation	[150, 151, 152, 153, 154, 155, 156, 157]
	Chitosan	Solution casting, compression molding, extrusion, electrospinning, ionic gelation	DIW, SLA, 2PP, DLP	2–2.7	20–330	140–150	102.5		Excellent viability, limited adhesion	Rapid degradation	[158, 159, 160, 161, 162, 163, 164]
	Collagen	Injection molding, extrusion	DIW, Inkjet, SLS, CAWS	3500	40	∼95	40–180	75–105	Excellent	Degradable both extracellularly and intracellularly	[165, 166, 167, 168, 169, 170]
	Fibrin	Electrospinning	DIW, Inkjet	0.01–15	0.013–0.133		53–62		Excellent	Enzymatic degradation	[171, 172, 173, 174, 175]
	Gelatin	Solution casting, extrusion, injection molding, compression molding, electrospinning, coacervation, emulsification, film coating	DIW, DLP, SLA	0.001–0.1	1–10	120–220	30–40 (Gel state)	10–35 (Aqueous solution)	Excellent	Enzymatic degradation	[176, 177, 178, 179, 180, 181]
	Hyaluronic acid	Solution casting, electrospinning, compression molding, extrusion	DIW, Inkjet, SLA, DLP	0.02–0.2	0.01–0.9	−80 to −48(Hydrated system)	241–247		Excellent viability, only physical interaction with cells	Rapid degradation	[182, 183, 184, 185]
	PHAs	Injection molding, extrusion	FDM, SLS, CAWS	3500	40	2–147	40–180	75–105	Excellent	Degradable both extracellularly and intracellularly	[165, 166, 167, 168, 186]
	Silk fibroin	Solution casting, electrospinning, spray coating	DIW, Inkjet, Binder Jetting, DLP	1000–15 000	500–25000	174–194	237–287	160–200	Excellent	Enzymatic degradation	[77, 187, 188, 189, 190, 191]
	Sodium alginate	Solution casting, ionic gelation, extrusion, emulsification	DIW, Inkjet, SLA, DLP	0.001–0.1	0.001–1	100–130 (Ionically crosslinked)	>99	Can crystallize at 4 oC	Excellent viability, lack of binding domains	Rapid degradation	[192, 193, 194, 195, 196, 197]
	Xylan	Extrusion, injection molding, compression molding, solution casting, calendaring, blow molding, compression coating	DIW	2735–3350	55–65	121–132	No T_m_		Excellent	Degrade over time	[198, 199, 200, 201, 202, 203, 204]

2PP, two‐photon polymerization; CAWS, computer‐aided wet‐spinning; cDLP, continuous digital light processing; DIW, direct ink writing; DLP, digital light processing; EHDP, electrohydrodynamic printing; FDM, fused deposition modeling; MJF, multi‐jet fusion; PAA, polyamino acid; PBS; poly(butylene succinates); PCL; polycaprone lactone; PDMS; polydimethylsiloxane; PEG; polyethylene glycol; PGA; poly(glycolic acid); PHAs; polyhydroxyalkanoates; PLA; polylactic acid; PPF; poly(propylene fumarate); TPU; thermoplastic polyurethane; SLA, stereolithography; SLM, selective laser melting; SLS, selective laser sintering.

### 3.2 Synthetic Polymers with Varying Biocompatibility and/or Biodegradability

#### 3.2.1 Poly (Amino Acids) (PAAs)

PAAs are a class of polymers characterized by the presence of amino acid units linked together through peptide bonds.^[^ ^101^ ^]^ Despite being present in the body, PAAs have been artificially synthesized since the 1950s, with one of the classic methods involving the ring‐opening polymerization of amino acid N‐carboxyanhydride to obtain high molecular weight PAAs.^[^ ^205^ ^]^ These polymers exhibit versatility due to their multifarious side chains, allowing for the formation of crosslinks and the detection of various markers.^[^ ^206^ ^]^ This adaptability enables PAAs to be utilized in multiple analytical methods such as electrochemical, fluorescence, photoluminescent, and colorimetric techniques in biosensing applications.^[^ ^101^ ^]^ These polymers also exhibit unique properties that make them suitable for various applications, including additive manufacturing. PAAs can be synthesized using techniques such as ring‐opening polymerization of amino acid derivatives or solid‐phase peptide synthesis. In additive manufacturing, PAAs can be processed using methods like FDM, where PAA filament is heated and extruded through a nozzle to build up layers, or SLA, which uses UV light to selectively cure liquid PAA resin layer by layer. These techniques enable the fabrication of complex three‐dimensional structures with precise control over geometry and composition.

PAAs possess diverse mechanical and thermal properties depending on the specific amino acids incorporated into their structure. Generally, PAAs exhibit good mechanical strength, flexibility, and toughness, making them suitable for applications requiring durable and resilient materials. Additionally, PAAs can have tunable thermal properties, with glass transition temperatures and melting temperatures that vary depending on the amino acid composition and polymer chain length. These properties allow PAAs to withstand a wide range of environmental conditions and processing temperatures, making them versatile materials for additive manufacturing and other engineering applications.

In terms of biocompatibility, PAAs are inherently biocompatible due to their peptide bond structure, which can be enzymatically hydrolyzed in biological environments.^[^ ^207^ ^]^ This property makes PAAs suitable for use in biomedical applications such as tissue engineering scaffolds, drug delivery systems, and implantable medical devices. Additionally, PAAs exhibit good cell viability and promote cell adhesion and proliferation, making them ideal materials for supporting tissue growth and regeneration. Commercial products based on PAAs include biodegradable sutures, wound dressings, and drug delivery matrices, demonstrating the wide‐ranging applications and potential of these polymers in the biomedical field. However, PAAs are inherently biocompatible due to their resemblance to natural proteins. Thus, controlling the degradation of PAAs poses a challenge, as the rate of enzymatic hydrolysis of the amide bond can vary depending on individual enzymatic activity.^[^ ^208^ ^]^ To address issues related to poor mechanical strength and enzymatic degradation, a new class of polymers derived from amino acids has emerged. These polymers feature a backbone formed by utilizing the side‐chain functional groups on α‐l‐amino acids or dipeptides, leading to the development of pseudopoly(amino acids).^[^ ^209^ ^]^ This class of materials includes three main categories: hydroxyproline‐derived polyesters, serine‐derived polyesters, and tyrosine‐derived polymers, each offering unique properties and potential applications in biomedical and biotechnological fields.^[^ ^210^, ^211^ ^]^

#### 3.2.2 Poly(Butylene Succinates) (PBS)

PBS is a synthetic biodegradable polymer characterized by its repeating units of butylene glycol and succinic acid. This polymer has gained attention in various applications due to its biocompatibility, biodegradability, and environmental friendliness.^[^ ^212^ ^]^ One of the key advantages of PBS is its biodegradability. It can be broken down by microorganisms in the environment,^[^ ^213^ ^]^ ultimately decomposing into harmless byproducts. This feature makes PBS an eco‐friendly choice for applications where biodegradability is essential, such as disposable packaging, agricultural films, and more. Moreover, PBS has found utility in the biomedical field, particularly in the development of biodegradable medical implants and drug delivery systems.^[^ ^212^ ^]^ Its ability to degrade naturally within the body while maintaining biocompatibility makes it a promising material for controlled drug release and tissue engineering applications.

PBS exhibits favorable mechanical and thermal properties, making it suitable for a wide range of applications.^[^ ^214^ ^]^ Its mechanical strength and flexibility make it valuable for use in products that require durability and stability during use. PBS typically exists as a solid at room temperature and can be processed using various manufacturing techniques (Table **2**). For example, PBS is known for its relatively low melting point, which enables it to be processed using methods such as injection molding and extrusion. Besides, PBS has the potential for 3D printing, but it is not as commonly used as some other thermoplastics in 3D printing. PBS's printability depends on factors like its grade, formulation, and processing temperature. While it may be compatible with certain FDM 3D printers that can reach the required extrusion temperature,^[^ ^215^ ^]^ it is not as prevalent in the 3D printing world as materials like PLA or ABS. Some industrial 3D printers, as well as customized or modified machines, may offer the capability to process PBS pellets or powders, especially if they can achieve the necessary processing conditions. Successful 3D printing with PBS requires careful consideration of material properties, extrusion temperatures, and printer compatibility, making it a less common choice compared to other thermoplastics.^[^ ^216^ ^]^

The PBS biodegradability and biocompatibility characteristics contribute to its growing importance in sustainable and environmentally conscious product development, particularly in the development of biodegradable medical implants and devices. Some examples of successful biomedical implants and medical devices made from PBS or PBS‐based materials include surgical sutures, drug delivery systems,^[^ ^217^ ^]^ orthopedic implants, tissue engineering scaffolds,^[^ ^218^ ^]^ wound dressings, and drug‐eluting stents. PBS‐based implants and devices provide benefits such as gradual degradation within the body, eliminating the need for removal, controlled drug release, and support for tissue regeneration. Their successful implementation requires rigorous testing, biocompatibility assessments, and regulatory approvals to ensure safety and efficacy in clinical applications. Ongoing research and innovation in biodegradable polymers may lead to further advancements in PBS‐based medical products.

#### 3.2.3 ε‐Polycaprolactone (PCL)

PCL is a versatile synthetic polymer with a well‐defined chemical structure. It is composed of repeating units of ε‐caprolactone monomers, forming a linear chain. PCL is known for its flexibility and biodegradability, making it a valuable material in various applications. PCL's molecular weight can vary, affecting its physical properties; however, it typically exists as a solid at room temperature. The polymer can be easily synthesized through the ring‐opening polymerization of ε‐caprolactone monomers, followed by processes like purification and formulation into various forms, such as pellets or filaments.

PCL is particularly favored in the field of 3D printing, thanks to its excellent printability (Table **2**). Its low melting point, typically around 60 °C to 70 °C, allows for easy extrusion and layer‐by‐layer deposition in FDM 3D printers.^[^ ^219^ ^]^ During printing, PCL exhibits minimal warping and good adhesion between layers, resulting in strong and cohesive prints. Its versatility extends to other 3D printing methods, such as SLS, where modified PCL pellets or powders may be used for specific applications. PCL's slow cooling rate aids in layer adhesion and reduces the risk of print defects. Additionally, PCL's solubility in various solvents, including chloroform, carbon tetrachloride, dichloromethane, and toluene, makes it compatible with processes like DIW, further expanding its utility in 3D printing and beyond.

PCL's biodegradability is another critical feature, with its degradation rate influenced by factors like molecular weight, environmental conditions, and crystallinity.^[^ ^220^, ^221^ ^]^ For example, PCL can exhibit a notable crystallinity of up to 69%,^[^ ^222^ ^]^ and its degradation initiates from its amorphous phase, potentially leading to an increase in crystallinity.^[^ ^223^ ^]^ The reduction in molecular weight occurs due to the cleavage of ester bonds, contributing to its biodegradation.^[^ ^221^, ^224^ ^]^ While the human body lacks specific enzymes for PCL degradation,^[^ ^225^ ^]^ the polymer can undergo autocatalytic degradation due to carboxylic acids released during hydrolysis.^[^ ^226^ ^]^ This property makes PCL suitable for applications in which controlled degradation is desired, such as in tissue engineering (e.g., soft tissue repair in hernia repair meshes and breast reconstruction), orthopedic implants (i.e., screws, pins, and plates), and medical devices (e.g., drug delivery microspheres, wound dressings and nerve conduits).^[^ ^227^ ^]^

#### 3.2.4 Polydimethylsiloxane (PDMS)

PDMS is a widely used silicone‐based polymer known for its unique properties and versatility in various applications. In additive manufacturing, PDMS can be utilized through techniques such as SLA, DLP, and DIW.^[^ ^228^, ^229^ ^]^ In SLA, liquid PDMS resin is selectively cured by ultraviolet (UV) light to create complex three‐dimensional structures layer by layer. DLP utilizes a projector to selectively cure liquid PDMS resin layer by layer using UV light. This technique enables the fabrication of complex PDMS structures with high resolution and surface quality. DIW, on the other hand, involves extruding PDMS ink through a nozzle onto a substrate to build up intricate designs. These additive manufacturing methods allow for the fabrication of customized PDMS components with precise control over geometry and structure.

PDMS exhibits advantageous mechanical and thermal properties that make it suitable for a wide range of applications. It possesses excellent elasticity, low surface tension, and high flexibility, allowing for deformation without permanent damage. Additionally, PDMS demonstrates good thermal stability, with a wide operating temperature range of ≈−50 to 200 °C. Its low glass transition temperature of ≈−125 °C enables PDMS to maintain flexibility and elasticity even at low temperatures, making it ideal for cryogenic applications. These properties make PDMS well‐suited for use in microfluidic devices, biomedical implants, flexible electronics, and other advanced engineering applications.

In terms of biocompatibility, PDMS is widely regarded as safe for use in biomedical and healthcare applications.^[^ ^230^ ^]^ It is inert, non‐toxic, and non‐reactive, making it suitable for contact with biological tissues and fluids. PDMS exhibits good cell viability and compatibility, making it an ideal material for fabricating medical devices such as catheters,^[^ ^231^ ^]^ prosthetic devices, and microfluidic systems.^[^ ^232^ ^]^ Moreover, PDMS is biologically inert and non‐biodegradable, meaning it does not undergo degradation in the body over time. Its biocompatibility and stability have led to the development of various commercial products, including medical implants, diagnostic tools, and wearable devices for monitoring health parameters.

#### 3.2.5 Polyethylene Glycol (PEG)

PEG, also known as PEO, is a synthetic polymer with a simple and repeating chemical structure. It consists of repeating units of ethylene glycol (─CH_2_─CH_2_─O─)_n_, where the “n” value represents the number of ethylene glycol units and determines the molecular weight of the polymer. PEG is more commonly used in the context of low to medium molecular‐weight polyethylene glycols, typically with molecular weights up to ≈20 000 g mol^−1^. PEGs are often liquids or waxy solids at room temperature. PEO is a term more commonly associated with higher molecular weight polyethylene glycols, often those with molecular weights exceeding 20 000 g mol^−1^. PEOs are typically solid materials at room temperature. PEG is a water‐soluble, odorless, and colorless polymer that can exist in various forms, including liquids, waxes, and solid powders, depending on its molecular weight. The physical structure of PEG can vary from low‐molecular‐weight liquids to high‐molecular‐weight waxy or solid substances, making it a versatile material employed in various manufacturing processes, including extrusion, solvent casting, injection molding, compression molding, and 3D printing technologies such as FDM, SLA, DLP, DIW, and SLS (Tables **1** and 2).^[^ ^233^, ^234^ ^]^

PEG exhibits desirable properties for these applications, including favorable mechanical and thermal characteristics (Table 2). Additionally, PEG stands out in terms of biodegradability and biocompatibility. Lower molecular weight PEG variants are more readily biodegradable. These molecules can be broken down by microorganisms present in the environment, such as bacteria and fungi.^[^ ^235^ ^]^ They can also undergo hydrolysis in aqueous environments, ultimately leading to the degradation of the polymer into smaller, less harmful compounds. Higher molecular weight PEO may be less biodegradable, as they are less soluble and more resistant to microbial degradation due to their size and structural complexity. Besides, factors such as temperature, pH, oxygen availability, and the presence of specific microorganisms all influence the rate and extent of biodegradation.

As an FDA‐approved biopolymer for a few products, PEG boasts good biocompatibility and biodegradability with degradation rates that can be tailored to suit specific needs. This makes PEG particularly valuable in biomedical, health, and pharmaceutical fields, where its biocompatible nature and controlled degradation profiles find numerous applications, from drug delivery systems to tissue engineering and medical devices. For example, MiraLAX is an over‐the‐counter laxative commonly used to relieve occasional constipation. It contains PEG 3350 as its active ingredient and has been FDA‐approved for this purpose. Besides, PEG is a common component in drug delivery systems,^[^ ^236^ ^]^ including injectable hydrogels and controlled‐release formulations. PEG‐based hydrogels have been used in ophthalmic applications, including contact lenses and intraocular lenses, to improve biocompatibility and patient comfort. Similarly, orthopedic implants and scaffolds designed for bone and cartilage repair, wound dressings, hernia repair or breast reconstruction, vascular grafts, and artificial blood vessels, have also incorporated PEG‐based hydrogels.^[^ ^237^, ^238^ ^]^

#### 3.2.6 Polyglycolic Acid (PGA)

PGA falls under the category of poly(α‐hydroxy acids), which can be synthesized through both poly‐condensation of glycolic acid and ring‐opening polymerization of glycolide.^[^ ^239^ ^]^ Glycolic acid, the monomer used in PGA production, is the smallest alpha‐hydroxy acid and can be sourced from natural origins like sugarcane and pineapple,^[^ ^240^ ^]^ though industrial‐scale production primarily relies on petrochemistry. Co‐polymerization of PLA and PGA has been a common strategy for enhancing material performance, allowing for adjustments in degradation time, mechanical properties, and gas barrier properties.^[^ ^240^ ^]^

PGA is a biodegradable polymer widely used in additive manufacturing processes due to its favorable properties and compatibility with various fabrication techniques. In general, PGA can be processed through methods such as melt extrusion and solution processing to create printable filaments or powders for additive manufacturing technologies like FDM. These methods enable the fabrication of complex structures with precise control over geometry, making PGA suitable for applications ranging from tissue engineering scaffolds to drug delivery systems.

When considering the physical properties of PGA, its mechanical and thermal characteristics play a crucial role in its performance. PGA exhibits robust mechanical properties (e.g., Young's modulus of GPa and strength of MPa), ensuring structural integrity in various applications. Moreover, PGA possesses favorable thermal stability, with a melting point higher than 200 degrees Celsius, enabling it to withstand the temperatures encountered during thermal‐based AM processes.

Originally developed as a suture material named Dexon as far back as 1962, PGA was engineered to retain mechanical stability for several weeks before rapidly absorbing into the body.^[^ ^241^ ^]^ In terms of biocompatibility, PGA demonstrates excellent compatibility with biological systems, making it suitable for medical applications. On one hand, its biodegradability ensures that PGA‐based implants or devices gradually degrade in the body over time, eliminating the need for removal surgeries and reducing the risk of complications. On the other hand, PGA and copolymerized PLA/PGA has been extensively studied for its effects on cell viability and tissue regeneration, with research indicating its ability to support cell growth and proliferation. Commercial products utilizing PGA in the biomedical field include surgical sutures, tissue engineering scaffolds, and drug delivery systems, highlighting its widespread use and importance in medical device manufacturing.

#### 3.2.7 Polylactic Acid (PLA)

PLA, an aliphatic polyester, with its monomer lactic acid, especially its L‐isomer, is naturally produced and metabolized through regular metabolic pathways in mammals.^[^ ^242^ ^]^ With an estimated annual production of 190 000 tons,^[^ ^243^ ^]^ PLA is primarily synthesized through condensation polymerization of lactic acid or ring‐opening polymerization of lactide. This synthesis yields various polymer types such as poly‐L‐lactic acid (PLLA), poly‐D‐lactic acid (PDLA), and poly‐D,L‐lactic acid (PDLLA), which differ based on the chiral structure of the monomers.^[^ ^244^ ^]^ PLA is also a widely used biodegradable polymer that can be derived from renewable resources such as corn starch or sugarcane. PLA's thermoplastic properties make it easily processable using traditional methods,^[^ ^245^ ^]^ enabling the production of films and fibers (Table 2). It is a popular choice for additive manufacturing due to its versatility, ease of processing, and environmentally friendly nature.^[^ ^246^ ^]^ PLA can be processed using various additive manufacturing techniques, including SLA, FDM, and SLS. In FDM, PLA filament is heated and extruded through a nozzle to build up layers, while SLA utilizes a UV laser to cure liquid resin layer by layer, and SLS employs a laser to sinter powdered PLA material. These methods allow for the creation of complex, custom‐designed objects with precise control over geometry.

PLA possesses favorable mechanical and thermal properties suitable for a wide range of applications. It exhibits moderate tensile strength, typically ranging from 40 MPa to 60 MPa, and an average Young's modulus of around 3 GPa, indicating its stiffness and ability to resist deformation. PLA also demonstrates good thermal stability, with a glass transition temperature of ≈50–65 °C and a melting temperature of ≈170–180 °C. These properties make PLA suitable for applications requiring dimensional stability and moderate mechanical strength, such as packaging materials, consumer goods, and medical devices.

In addition to its mechanical and thermal properties, PLA is biocompatible and biodegradable, making it well‐suited for biomedical applications. PLA degrades into non‐toxic lactic acid through simple hydrolysis in the body without the need for enzyme involvement,^[^ ^242^ ^]^ making it compatible with human tissues and reducing the risk of adverse reactions. It has been extensively studied for use in tissue engineering scaffolds, drug delivery systems, and medical implants.^[^ ^247^, ^248^ ^]^ Commercial products incorporating PLA include surgical sutures, orthopedic implants, and drug delivery devices. PLA's biodegradability also contributes to its appeal in sustainable packaging and disposable products, where it can reduce environmental impact compared to traditional petroleum‐based plastics.

#### 3.2.8 Poly(Propylene Fumarate) (PPF)

PPF is a biodegradable polyester derived from fumaric acid, a chemical abundant in nature and a component of the Krebs cycle,^[^ ^249^ ^]^ also serves as a food additive known for its fruity taste. As an unsaturated polyester, PPF contains double bonds in its backbone, offering the potential for cross‐linking. Various compounds like methyl methacrylate (MMA), poly (ethylene glycol)‐dimethacrylate (PEG‐DMA), and N‐vinyl pyrrolidone (NVP) have been utilized as thermo‐crosslinking agents for PPF. PPF is a biocompatible and slowly biodegradable polymer that has garnered significant attention in the field of biomedical engineering and regenerative medicine (e.g., bone engineering or tissue reconstructions).^[^ ^135^ ^]^ As a thermoplastic polymer, PPF exhibits favorable properties for additive manufacturing processes. Its processability via techniques, such as SLA, FDM, and DLP, enables the fabrication of intricate three‐dimensional structures with precise control over geometry and porosity.^[^ ^250^, ^251^ ^]^ For example, PPF exhibits photo‐crosslinking capabilities,^[^ ^252^, ^253^ ^]^ enabling its suitability for 3D printing with enhanced temporal and spatial control.^[^ ^135^ ^]^ Commonly, copolymerization with poly (ethylene glycol) (PEG) is employed to modify PPF properties,^[^ ^254^ ^]^ resulting in poly(propylene fumarate‐co‐ethylene glycol) (P(PF‐co‐EG)) or oligo(poly(ethylene glycol) fumarate) (OPF).^[^ ^255^ ^]^ This incorporation of PEG enhances the material's hydrophilicity while retaining available sites for covalent crosslinking on the PPF portion. Consequently, the fumarate to PEG ratio impacts the post‐crosslinking hydrogel swelling, offering tunability in material properties. PPF‐based scaffolds and implants can be tailored to match the mechanical properties and architecture of native tissues, making them promising candidates for tissue engineering applications.

In terms of physical properties, PPF possesses desirable mechanical characteristics and thermal stability suitable for biomedical applications. It exhibits excellent tensile strength and modulus (Table **2**), allowing for the fabrication of load‐bearing implants and scaffolds that can withstand mechanical stresses in physiological environments. Additionally, PPF demonstrates good thermal stability (Table **2**), retaining its structural integrity at elevated temperatures encountered during processing and sterilization. These properties make PPF an attractive material for manufacturing implants, tissue scaffolds, and drug delivery systems intended for use in clinical settings.

Biocompatibility and biodegradability are crucial considerations for biomaterials used in biomedical applications, and PPF excels in both aspects. As with many synthetic biodegradable polymers, the degradation of PPF‐based molecules occurs via hydrolysis of ester bonds along their backbones.^[^ ^255^ ^]^ PPF has been shown to support cell adhesion, proliferation, and differentiation, making it conducive to tissue regeneration and integration with host tissues. Furthermore, PPF undergoes gradual degradation via hydrolysis of its ester bonds, with degradation products that are metabolizable and non‐toxic to the body. This controlled degradation process ensures that PPF‐based implants and scaffolds maintain their structural integrity while facilitating tissue ingrowth and remodeling. Commercially, PPF‐based products such as tissue scaffolds, bone graft substitutes, and drug delivery systems are being developed and evaluated for various clinical applications, demonstrating the potential of PPF in advancing regenerative medicine and personalized healthcare.

#### 3.2.9 Thermoplastic Polyurethanes (TPU)

TPU is a versatile class of polymers widely used in various industries due to their exceptional mechanical properties, flexibility, and chemical resistance. TPU is synthesized through the reaction of diisocyanates with diols or polyols, resulting in a linear polymer chain with urethane linkages. This chemical structure imparts thermoplastic behavior, allowing TPUs to be melted, molded, and extruded multiple times without undergoing significant degradation. In additive manufacturing, TPUs are employed in processes such as FDM, SLS, and DIW,^[^ ^256^, ^257^ ^]^ where their excellent melt processability enables the production of complex geometries with high precision.

TPU exhibits a unique combination of mechanical and thermal properties that make it suitable for a wide range of applications. It possesses excellent tensile strength, tear resistance, and elasticity, making it ideal for applications requiring flexibility and durability. Additionally, TPUs have good abrasion resistance and can withstand harsh environmental conditions, such as exposure to chemicals and UV radiation. In terms of thermal properties, TPUs have a relatively low melting point, typically ranging from 150 °C to 220 °C, which facilitates their processing in additive manufacturing processes. Furthermore, TPUs exhibit good thermal stability, retaining their mechanical properties over a wide temperature range, from −40 °C to 100 °C or higher, depending on the specific formulation.

In biomedical and healthcare applications, TPUs have gained significant attention due to their biocompatibility, low toxicity, and potential for biodegradability. TPUs are commonly used in medical devices, prosthetics, and implants, where they come into direct contact with biological tissues and fluids. Their compatibility with human tissues and fluids makes them suitable for long‐term implantation without eliciting adverse reactions or inflammation. Although most TPU materials usually referred to are non‐degradable, some TPU formulations have been engineered to be biodegradable, enabling them to degrade over time in the body through hydrolysis or enzymatic processes.^[^ ^258^ ^]^ These TPUs typically contain polyester polyols, aliphatic diisocyanates, and other chain extenders,^[^ ^259^ ^]^ and the new concept about dynamic covalent chemistry is adding more potential to the biodegradability of TPUs.^[^ ^260^ ^]^ Commercial products incorporating TPU include medical tubing, catheters, wound dressings, orthopedic implants, and cardiovascular devices, highlighting their widespread use in the healthcare industry.

#### 3.2.10 Other Biopolymers with Limited 3D Printing History

Polyanhydride (PA): Since their initial synthesis was reported in 1909,^[^ ^261^ ^]^ numerous PA structures have been developed,^[^ ^262^ ^]^ including aliphatic,^[^ ^263^ ^]^ unsaturated,^[^ ^264^ ^]^ aromatic,^[^ ^265^ ^]^ poly(ester anhydride),^[^ ^266^ ^]^ poly(ether anhydride),^[^ ^267^ ^]^ fatty acid‐based, and amino acid‐based variants.^[^ ^268^ ^]^ PA represents a biodegradable polymer class particularly relevant to drug delivery applications.^[^ ^269^ ^]^ They exhibit favorable in vivo biocompatibility,^[^ ^270^ ^]^ undergoing degradation into non‐toxic diacid byproducts that can be safely eliminated from the body as metabolites.^[^ ^271^ ^]^ Some PAs also offer the capability of photocrosslinking.^[^ ^272^ ^]^ Due to the limitation in rheology and composition control, efforts are focused on developing novel PA‐based printable materials, such as PA‐based composites or blends with other polymers, to improve print resolution, mechanical properties, and degradation behavior. With attributes such as low‐cost building blocks, one‐step synthesis, processibility at low temperatures, well‐defined polymer structure, and predictable biodegradation, PAs have shown promise in diverse medical applications ranging from brain tumor^[^ ^273^ ^]^ and eye disorder treatments,^[^ ^274^ ^]^ to addressing osteomyelitis,^[^ ^275^ ^]^ and others.^[^ ^276^, ^277^ ^]^

Polydioxanone (PDX): PDX has the monomer, paradioxanone, which undergoes polymerization via a ring‐opening reaction to form PDX, resulting in a poly (ether‐ester) structure.^[^ ^278^ ^]^ Compared to sutures made from PLA, monofilament PDX that initially serving as a monofilament suture for wound closure from the 1980s degrades more rapidly.^[^ ^279^ ^]^ The rate of degradation depends on various factors such as the molecular weight of the polymer, the surrounding environment, and the specific application of the PDX implant or device. Generally, PDX degrades at a rate that matches the healing process of the tissue it is supporting, making it suitable for use in temporary medical implants like sutures and tissue scaffolds. With mechanical properties on par with those of the native ECM, PDX has found application in artificial vascular conduits.^[^ ^280^ ^]^ Its shape memory property enhances graft resilience,^[^ ^280^ ^]^ providing resistance to rebound and kinking, although its use with spooled shapes may pose challenges.^[^ ^281^ ^]^ Other molecules in this category include, but are not limited to, poly(1,4‐dioxane), polyoxalate, and poly(p‐dioxanone).^[^ ^211^ ^]^ PDX has limited applicability in 3D printing compared to other polymers due to its unique properties and processing requirements. While PDX can be processed using conventional methods such as melt extrusion, it presents challenges in additive manufacturing due to its relatively high melting point and viscosity.

Poly(ortho esters) (POE): POEs emerged in the realm of drug delivery as a novel solution designed to erode solely at the surface layers, ensuring consistent drug release kinetics when uniformly dispersed.^[^ ^282^ ^]^ This unique property arises from their inherent hydrophobicity, which limits water penetration into the bulk material, coupled with the rapid hydrolysis of the polymer backbone.^[^ ^282^ ^]^ The degradation process typically involves the cleavage of ester linkages by water molecules, resulting in the breakdown of the polymer chains into smaller, biocompatible molecules.^[^ ^211^, ^282^ ^]^ Importantly, the degradation of POEs can be tailored to achieve specific degradation rates by modifying the chemical structure of the polymer. This tunability allows for the design of POEs with degradation profiles suited to different applications, such as drug delivery systems or tissue engineering scaffolds. Additionally, POEs can degrade into non‐toxic byproducts, making them suitable for use in biomedical devices intended for implantation or temporary therapeutic purposes. However, the quest for ideal POEs has been riddled with challenges and obstacles. Generally, four distinct families of POEs have been developed, each grappling with its own set of limitations, including the first three of i) the production of acidic hydrolysis products, ii) excessive stability under physiological conditions, and iii) issues related to reproducibility.^[^ ^283^ ^]^ Conversely, the fourth family exhibits promise due to its versatile synthesis methods. While ongoing research on POEs is underway, their commercialization remains limited compared to other biodegradable synthetic polymers like PLA and PGA.^[^ ^283^ ^]^ While POEs can be synthesized into biodegradable polymers, their 3D printability is not widely reported in the literature.^[^ ^284^, ^285^ ^]^ For example, since POEs have unique degradation properties and are typically processed using solvent‐based methods rather than melt‐based methods like extrusion‐based 3D printing, their direct 3D printability may be limited.

Polyphosphazenes (PPHOSs): PPHOSs are characterized by their distinctive backbone structure, which consists of alternating phosphorus and nitrogen atoms,^[^ ^286^ ^]^ making them hybrid inorganic–organic macromolecules. While the majority of PPHOSs, numbering over 700 different variants, exhibit hydrolytic stability, the choice of organic side groups attached to each phosphorus atom can render them hydrolytically unstable, leading to biodegradability.^[^ ^287^ ^]^ Upon degradation, these hydrolytically sensitive PPHOSs undergo hydrolysis to form phosphate and ammonia from their backbone, along with the release of free side groups.^[^ ^288^ ^]^ Since phosphate can be metabolized and ammonia can be excreted from the body, the degradation products of PPHOSs are considered benign.^[^ ^289^ ^]^ Aminated PPHOSs constitute the primary class of degradable PPHOSs, while some alkoxy‐substituted variants also fall under this category.^[^ ^290^ ^]^ Due to the diverse nature of pendant groups, PPHOSs can exhibit either bulk erosion mode, similar to PLA and PGA, or surface erosion mode, akin to polyanhydrides and POEs.^[^ ^291^ ^]^ While PPHOSs offer intriguing properties such as biodegradability and tunable degradation rates, their application in 3D printing is not as well‐established as other polymers. Currently, there is limited literature on the direct 3D printing of PPHOSs, likely due to challenges associated with their processing and rheological behavior. By combining PPHOSs with other polymers or additives that enhance printability, such as plasticizers or processing aids, researchers aim to overcome hurdles related to filament extrusion, printing resolution, and mechanical properties.

### 3.3 Natural Macromolecules

Natural polymers, aptly named for their origin from plants or animals,^[^ ^292^ ^]^ closely mimic the ECM of native tissues, fostering excellent cellular attachment and minimal immunological reactions.^[^ ^293^ ^]^ Additionally, these polymers typically exhibit remarkable biodegradability in the presence of enzymes,^[^ ^293^, ^294^ ^]^ obviating the need for removal surgeries. Examples of natural polymers abound and encompass proteins (e.g., collagen, gelatin, fibrinogen, and silk),^[^ ^295^, ^296^ ^]^ polysaccharides (e.g., alginate, HA, chitosan, cellulose),^[^ ^297^, ^298^ ^]^ and bacterial polyesters (e.g., PHAs and polyhydroxybutyrates (PHBs)).^[^ ^299^, ^300^ ^]^

#### 3.3.1 Agarose

Agarose belongs to the class of polysaccharide and is extracted from abundant marine red algae.^[^ ^301^ ^]^ It accounts for the gelling part agar and includes 1,3‐linked β‐D‐galactose and 1,4‐linked 3,6‐anhydro‐α‐L‐galactose on its backbone.^[^ ^302^ ^]^ Besides the resemblance to the ECM as other natural polymers, its thermos‐reversible gelation behavior garners scientific interest. It gels at 30–40 °C and melts at 80–90 °C without the need for crosslinking agents, such as genipin,^[^ ^301^ ^]^ owing to the presence of oxygen and hydrogen in the side groups. The transition temperatures vary depending on the molecular weight, though the average molecular weight of agarose is ≈100 kDa.^[^ ^302^ ^]^ In the form of a hydrogel, DIW is the most compatible 3D printing method for agarose. And it is added to materials systems such as gelatin and alginate during 3D printing to modify the gelation behavior, mechanical performance, printability, and porosity.^[^ ^303^, ^304^ ^]^

Compared to synthetic polymers, many natural polymers, including agarose, do not possess high stiffness or tensile strength. Therefore, agarose is more frequently utilized in soft tissue engineering applications.^[^ ^305^ ^]^ Its shear‐thinning behavior in the solution state favors DIW printing, endowing it with good printability. The elasticity and porosity of agarose gels can be adjusted with the concentration and gelation conditions. Yet, the most notable feature of agarose is still its self‐gelling behavior.

With a high‐water absorbance capacity, agarose gels possess adequate biocompatibility for cell proliferation and differentiation. However, the cell attachment is limited due to a lack of adhesion sites. Surface modification is required to enhance cell adhesion.^[^ ^306^, ^307^ ^]^ Even so, agarose‐based biomaterials cover a vast range of biomedical applications, including artificial pancreas,^[^ ^308^ ^]^ bone and cartilage,^[^ ^309^ ^]^ spermatogenesis,^[^ ^310^ ^]^ neurogenesis, injectable hydrogels,^[^ ^311^ ^]^ drug delivery,^[^ ^302^ ^]^ and wound healing.^[^ ^312^ ^]^ 3D‐printed tissue scaffolds have also been reported.^[^ ^313^ ^]^ In summary, agarose's biocompatibility, gelation properties, and ease of use have led to its widespread adoption in various biomedical applications. Its versatility makes it a valuable material for researchers and practitioners working on diverse projects ranging from molecular biology techniques to advanced tissue engineering and drug delivery systems.

#### 3.3.2 Bacterial Cellulose

Cellulose, the most abundant organic polysaccharide on Earth, serves as a fundamental structural component in the cell walls of plants. It is derived from renewable resources,^[^ ^314^ ^]^ such as wood pulp and cotton linters, comprising 40–50% of wood and over 90% of cotton.^[^ ^315^ ^]^ Structurally, cellulose consists of D‐glucopyranose ring units connected by β‐1,4‐glycosidic bonds,^[^ ^316^ ^]^ with inter‐ and intra‐chain hydrogen bonding providing stability.^[^ ^317^ ^]^ While extraction from plant sources involves removing lignin and hemicellulose through harsh treatments, bacterial cellulose (BC) offers a purer alternative. BC, composed solely of glucose monomers, boasts a crystallinity of 84–89%^[^ ^318^ ^]^ as compared to the plant cellulose crystallinity of 40–60%,^[^ ^319^ ^]^ resulting in impressive mechanical properties with a high Young's modulus (15–35 GPa) and tensile strength (200–300 MPa),^[^ ^148^ ^]^ making it ideal for reinforcement in 3D printing applications.^[^ ^93^, ^320^, ^321^ ^]^ Cellulose is extensively used in additive manufacturing also due to its versatile properties and biocompatibility. The material is often processed into filaments or powders for use in various 3D printing techniques, including FDM, SLA, DLP, and SLS. Cellulose‐based filaments exhibit good printability and compatibility with existing 3D printers, making them suitable for a wide range of applications in biomedical engineering, packaging, and sustainable manufacturing.

In terms of physical properties, cellulose possesses remarkable mechanical strength and thermal stability, rendering it suitable for load‐bearing applications. Its tensile strength and Young's modulus can be comparable to some synthetic polymers, making it an attractive option for structural components in tissue engineering scaffolds and biodegradable implants. Moreover, cellulose exhibits excellent thermal resistance, allowing it to withstand elevated temperatures during processing without significant degradation. These properties make cellulose‐based materials highly desirable for applications requiring robust mechanical performance and thermal stability.

Biocompatibility is a key advantage of cellulose‐based materials, as they are inherently biocompatible and non‐toxic. For example, acetobacter xylinum, an aerobic gram‐negative bacterium,^[^ ^322^ ^]^ is recognized as a highly efficient producer of BC. It metabolizes various sugars and secretes protofibrils of glucose chains, which assemble into nanofibril cellulose ribbons.^[^ ^323^ ^]^ Besides, cellulose scaffolds support cell attachment, proliferation, and differentiation, making them suitable for tissue engineering and regenerative medicine applications. Furthermore, cellulose is biodegradable, meaning it can be broken down by biological processes into harmless byproducts, making it environmentally friendly. Commercially, cellulose‐based products such as wound dressings, drug delivery systems, and food packaging materials are widely available, showcasing the versatility and practicality of cellulose in various industries. Despite its degradable nature, the degradation of cellulose in animal and human bodies is limited due to the absence of specific hydrolases that can be produced by fungi and microbes.^[^ ^324^ ^]^ As a result, cellulose‐based implants must be carefully considered for their compatibility with the in vivo environment. Thus, while cellulose offers promising mechanical properties and biocompatibility, its biodegradability should be considered in the design of biomedical applications, ensuring compatibility with the body's natural processes for optimal performance and longevity.

#### 3.3.3 Carrageenan

Carrageenan is another natural polysaccharide extracted from red seaweeds, specifically from several species of Rhodophyta, including *Chondrus*, *Eucheuma*, *Gigartina*, and *Hypnea*.^[^ ^325^ ^]^ It has a relatively long history of being used as an ingredient in food and cosmetics due to its gelling, thickening, stabilizing, and emulsifying properties.^[^ ^326^ ^]^ Back in the 1960s, the commercial cultivation of the tropical seaweed *Kappaphycus alvarezii* has already boosted carrageenan production.^[^ ^327^ ^]^ Chemically, it is a sulfated galactan composed of D‐galactose residues linked alternately in 3‐linked‐β‐D‐galactopyranose and 4‐linked‐α‐D‐galactopyranose units.^[^ ^328^ ^]^ Other carbohydrate residues, such as xylose, glucose, and uronic acids, and substituents, such as methyl ethers, might present as well.^[^ ^327^ ^]^

Covalently connected to the carbon atoms C‐2, C‐4, or C‐6 of the galactose residues via ester linkages, the ─O─SO_3_^−^ makes carrageenan negatively charged. And the degree of sulfation, usually ranges from 0 to 41%, determines the type of carrageenan.^[^ ^327^ ^]^ The most common types are the κ‐, τ‐, and λ‐carrageenan, with calculated sulfate contents of 20 wt%, 33 wt%, and 41 wt%, respectively.

The difference in sulfate contents affects the mechanical and rheological properties of carrageenan. λ‐carrageenan loses its ability to form gels, while κ‐ and τ‐carrageenan form firm and soft gels, respectively.^[^ ^327^ ^]^ This process is due to the formation of helix structure by contacting gel‐inducing cations, such as K^+^ and Ca^2+^. This gelation behavior contributes to its suitability for DIW applications.^[^ ^152^ ^]^ The wide span of the mechanical properties of different types of carrageenan has earned them a place in food industry (i.e., as binders and stabilizers),^[^ ^329^ ^]^ the daily chemistry industry (i.e., in toothpaste, firefighting foam, and cosmetic creams), and pharmaceuticals.^[^ ^330^ ^]^

Although carrageenan can undergo enzymatic degradation,^[^ ^331^ ^]^ there is no evidence that the human body produces carrageenanases. Subcutaneously, it degrades through hydrolysis of either the crosslinker or the polymer backbone.^[^ ^332^ ^]^ Nevertheless, its applications in drug delivery, even biomacromolecule and cell delivery, are rapidly expanding.^[^ ^328^ ^]^ Carrageenan demonstrates low cytotoxicity in vitro and induces low inflammatory response in vivo,^[^ ^333^ ^]^ leading to its vast applications in the fields of wound healing, tissue engineering, biocompatible coatings, cell encapsulation, dental application, and antimicrobial.

#### 3.3.4 Chitosan

Chitosan is derived from chitin through deacetylation, during which procedure the deacetylation process converts repeating N‐acetyl‐D‐glucosamine units into β‐1,4‐D‐glucosamine, introducing crucial amino groups to the molecule and enhancing its biological effects and water solubility for its versatility and biocompatibility.^[^ ^334^, ^335^ ^]^ Chitin, a natural polysaccharide found abundantly in the exoskeletons of crustaceans and insects, serves as the precursor to chitosan.^[^ ^336^ ^]^ Upon deacetylation, chitin's acetyl groups are removed, resulting in chitosan, which exhibits enhanced solubility in acidic solutions.^[^ ^337^ ^]^ However, delineating chitosan from chitin is not straightforward, given the variable degree of deacetylation.^[^ ^338^, ^339^ ^]^ Commonly sourced from abundant chitin‐rich shells of shrimp, crab, lobster, crayfish, and oyster,^[^ ^340^, ^341^, ^342^ ^]^ chitosan's manufacturing process varies. This characteristic makes chitosan highly amenable to various manufacturing processes, including additive manufacturing techniques like DIW. In such applications, chitosan can be used as a bioink for the fabrication of tissue scaffolds, drug delivery systems, and medical implants, offering a promising avenue for the development of personalized biomedical devices. Besides, utilizing chitosan in additive manufacturing is facilitated by simple alkaline treatment, prompting gelation as the polymer is generally soluble in acidic conditions.^[^ ^343^, ^344^ ^]^ Furthermore, it forms physical crosslinks with tripolyphosphate/sodium tripolyphosphate (TPP)^[^ ^345^ ^]^ and negatively charged biocompatible polymers such as alginate,^[^ ^346^ ^]^ gelatin,^[^ ^347^ ^]^ dextran sulfate,^[^ ^348^ ^][^ ^349^ ^]^ as well as chemical crosslinks with agents like glutaraldehyde,^[^ ^350^ ^]^ genipin,^[^ ^351^ ^]^ and 1‐ethyl‐3‐(3‐dimethylaminopropyl)‐carbodiimide (EDC).^[^ ^352^ ^]^ Extensive exploration of chitosan's applications in 3D printing has been comprehensively reviewed.^[^ ^353^ ^]^

In addition to its processability, chitosan boasts favorable mechanical and thermal properties that contribute to its suitability for additive manufacturing. Chitosan‐based materials exhibit good tensile strength and elasticity, making them suitable for constructing load‐bearing structures in tissue engineering and regenerative medicine. Furthermore, chitosan exhibits thermal stability at elevated temperatures, allowing for precise control over the printing process without compromising the integrity of the final product. These properties make chitosan an attractive candidate for use in additive manufacturing applications where structural integrity and thermal stability are paramount.

Beyond its physical attributes, chitosan is highly biocompatible and biodegradable, rendering it suitable for a wide range of biomedical applications. While chitosan exhibits a relatively slower degradation rate compared to collagen and fibrin,^[^ ^354^ ^]^ its enzymatic degradation in the body, facilitated by enzymes like lysozyme, allows for the hydrolysis of β (1‐4) linkages.^[^ ^353^, ^355^ ^]^ Due to its resemblance to glycosaminoglycans, a component of the ECM, chitosan promotes cell adhesion and proliferation, facilitating tissue regeneration and wound healing processes. Moreover, chitosan undergoes enzymatic degradation in the presence of lysozyme and other chitinolytic enzymes found in the human body, ensuring its safe and gradual breakdown into non‐toxic byproducts. Commercially, chitosan‐based products are utilized in wound dressings, drug delivery systems, and tissue engineering scaffolds, highlighting their widespread adoption in the biomedical field.

#### 3.3.5 Collagen

Collagen, often regarded as the most abundant and constitutes up to one‐third of the total protein content in the human body, plays a vital role in providing structural support and integrity to various tissues, including skin, bone, cartilage, and tendons.^[^ ^356^ ^]^ At least 28 types of collagen have been discovered, consisting of different α chains.^[^ ^357^ ^]^ Generally, types I, II, III, V, and XI appear fibrillar and can be found in connective tissues,^[^ ^358^ ^]^ while type IV is the major basement membrane collagen.^[^ ^359^ ^]^ Other types are present in smaller quantities in tissue and serve as connecting elements between these major structures.^[^ ^358^ ^]^ As a natural polymer, collagen possesses remarkable biocompatibility, making it a valuable biomaterial for a wide range of applications in tissue engineering and regenerative medicine. Derived from connective tissues of animals, particularly bovine and porcine sources, collagen can be processed into various forms suitable for additive manufacturing. Through techniques such as electrospinning and 3D bioprinting,^[^ ^360^ ^]^ collagen‐based scaffolds and constructs can be fabricated with precise control over their geometry and architecture, mimicking the native ECM to promote cell adhesion, proliferation, and tissue regeneration.

In terms of physical properties, collagen exhibits exceptional mechanical strength and flexibility, characteristics essential for supporting mechanical loads and maintaining structural integrity in tissues. Its unique triple helical structure, composed of three polypeptide chains intertwined in a rope‐like manner, contributes to its mechanical resilience. Moreover, collagen's thermal properties, including its melting temperature and heat stability, are crucial considerations in additive manufacturing processes. The thermal processing of collagen‐based materials must be carefully controlled to preserve its structural integrity and bioactivity while achieving the desired mechanical properties in the final fabricated constructs.

Biodegradability is a key feature of collagen‐based biomaterials, as they are designed to degrade and remodel within the body as new tissue forms. Collagen scaffolds undergo enzymatic degradation by matrix metalloproteinases (MMPs) and other proteases present in the extracellular environment, leading to the gradual breakdown of the scaffold material and the incorporation of new tissue components. This controlled degradation process allows for the seamless integration of the scaffold with the surrounding tissue and facilitates the regeneration of functional tissue structures. Besides, collagen features a characteristic structure composed of three polypeptide chains, known as α chains, forming a triple‐helical domain prevalent across the entire collagen superfamily.^[^ ^361^ ^]^ This structural configuration underpins the piezoelectric effects observed in collagen crystals, where hydrogen bond polarization or displacement within the polypeptide chains induces this phenomenon.^[^ ^362^ ^]^ Leveraging this property, researchers have developed collagen‐based piezoelectric sensors for real‐time physiological signal monitoring.^[^ ^363^, ^364^, ^365^ ^]^ Additionally, the attachment ligands present in collagen molecules facilitate cell adhesion and mitigate immune reactions,^[^ ^366^ ^]^ rendering collagen widely used in bioprinting applications, albeit requiring reinforcement to bolster its relatively weak mechanical properties^[^ ^360^, ^367^ ^]^ Commercially, collagen‐based products are widely used in wound healing dressings, tissue engineering scaffolds, and cosmetic applications, demonstrating the broad utility and versatility of collagen in the biomedical field.

#### 3.3.6 Fibrinogen (Fibrin)

Fibrinogen is a glycoprotein present in blood plasma and plays a crucial role in the blood clotting process, specifically in the formation of fibrin clots. Structurally, it is composed of three pairs of polypeptide chains, namely alpha, beta, and gamma chains,^[^ ^368^ ^]^ which are interconnected by disulfide bonds as present in human blood plasma and plays a pivotal role in hemostasis or wound healing^[^ ^369^ ^]^ by converting into an insoluble state known as fibrin. Upon activation by thrombin, fibrinogen is cleaved into fibrin monomers, which then polymerize to form a stable fibrin meshwork, providing the structural framework for blood clot formation.^[^ ^368^ ^]^ This unique ability to undergo polymerization makes fibrinogen a promising material for additive manufacturing^[^ ^370^, ^371^, ^372^ ^]^ by directly exposing it to thrombin, enabling the fabrication of intricate 3D structures with precise control over spatial distribution and morphology^[^ ^373^ ^]^ particularly useful in tissue engineering and regenerative medicine.^[^ ^370^, ^371^, ^372^ ^]^

In terms of physical properties, fibrinogen exhibits viscoelastic behavior, allowing it to form flexible and pliable fibrin clots that can withstand mechanical stresses. Its mechanical properties, including tensile strength and elasticity, can be modulated by altering the concentration of fibrinogen in solution or by incorporating other bioactive molecules. Moreover, fibrinogen possesses thermal stability, maintaining its structural integrity under physiological conditions. These properties make fibrinogen an attractive candidate for additive manufacturing processes such as bioprinting, where precise control over mechanical properties is essential for fabricating functional tissue constructs.

Biocompatibility is a key aspect of fibrinogen, as it is a naturally occurring protein found in the human body and is involved in essential physiological processes. Fibrinogen promotes cell adhesion, proliferation, and migration, making it suitable for tissue engineering applications where cell viability is crucial. Furthermore, fibrinogen is biodegradable, as it undergoes enzymatic degradation by proteolytic enzymes such as plasmin. This controlled degradation process ensures that fibrin clots are gradually replaced by newly formed tissue during the wound‐healing process. Commercially, fibrinogen‐based products are available for various medical applications, including surgical sealants, hemostatic agents, and tissue engineering scaffolds, demonstrating its widespread use and clinical relevance in the field of biomedicine.

#### 3.3.7 Gelatin

Gelatin, derived from the hydrolysis of collagen (i.e., destruction of cross‐linkages between the polypeptide chains and some breakage of polypeptide bonds), is a widely used natural polymer in various fields, including additive manufacturing.^[^ ^374^ ^]^ It possesses a unique combination of properties, such as the retaining crucial biological motifs of the Arg‐Gly‐Asp sequence, making it suitable for bioprinting applications.^[^ ^375^ ^]^ In the realm of 3D printing, GelMA (gelatin methacryloyl) serves as a prevalent UV‐curable feedstock,^[^ ^376^ ^]^ synthesized by methacrylate substitution of gelatin's amine‐containing side groups.^[^ ^377^, ^378^ ^]^ This transformation enables GelMA to undergo UV‐induced chemical crosslinking,^[^ ^379^ ^]^ allowing for on‐the‐fly curing during the printing process.

In terms of mechanical and thermal properties, gelatin demonstrates favorable characteristics for additive manufacturing processes. It forms thermally reversible gels, enabling the fabrication of complex structures with precise control over shape and geometry. Additionally, gelatin‐based bioinks possess tunable mechanical properties, depending on factors such as concentration, crosslinking density, and processing conditions. This versatility allows for the customization of bioinks to match the mechanical properties of native tissues, providing support and structural integrity to printed constructs.

Gelatin exhibits excellent biocompatibility, as it closely resembles the ECM found in human tissues. This compatibility promotes cell adhesion, proliferation, and differentiation, making gelatin‐based bioinks ideal for tissue engineering and regenerative medicine applications.^[^ ^380^ ^]^ Moreover, gelatin is biodegradable, allowing for the gradual degradation of printed constructs over time as new tissue forms. Yet, its derivatives are more commonly used for printing applications. Besides the GelMA, thiol‐ene reactions, thiol‐Michael additions, Diels‐Alder‐based click systems, and Schiff's‐base reaction can be used for UV‐crosslinking of gelatin.^[^ ^381^ ^]^ Commercially, gelatin‐based products are widely available in the biomedical and pharmaceutical industries. These products include wound dressings, drug delivery systems, and tissue engineering scaffolds.^[^ ^382^ ^]^ Gelatin‐based scaffolds have been used in clinical applications for skin regeneration, bone repair, and cartilage regeneration. Furthermore, Unlike native collagen, gelatin exhibits solubility in cold water, swelling to form a colloidal aqueous solution above 40 °C.^[^ ^383^ ^]^ As a result, gelatin is commonly used as a food additive, photography, biomedicine, and in the production of capsules for oral medication. Its safety, biocompatibility, and versatility make gelatin a valuable material for additive manufacturing and various biomedical applications.

#### 3.3.8 Hyaluronic Acid (HA)

HA is a naturally occurring polysaccharide^[^ ^384^ ^]^ found in the ECM of connective tissues, such as skin, cartilage, eyes and umbilical cord.^[^ ^385^ ^]^ It is composed of repeating disaccharide units of glucuronic acid and N‐acetylglucosamine, rendering it hydrophilic and negatively charged, and it plays a crucial role in tissue hydration, lubrication, and wound healing.^[^ ^386^ ^]^ In additive manufacturing, HA has been utilized as a bioink in bioprinting due to its ability to form hydrogels under physiological conditions. These hydrogels can mimic the natural ECM, providing a supportive environment for cell growth and tissue regeneration. Additive manufacturing techniques such as inkjet printing, extrusion‐based printing, and SLA have been employed to fabricate complex 3D structures with precise control over HA distribution and architecture. The physical properties of HA hydrogels can be adjusted by varying factors such as HA concentration, crosslinking density, and molecular weight. HA hydrogels exhibit excellent water retention capacity, which is beneficial for maintaining a hydrated microenvironment conducive to cell proliferation and migration. Additionally, HA hydrogels possess shear‐thinning behavior, meaning they can flow under shear stress during the printing process and quickly recover their gel‐like state upon removal of the stress.^[^ ^387^, ^388^ ^]^ As a result of this, researchers have integrated HA into bioinks to serve as a biocompatible viscosity modifier or mechanical enhancer. This rheological property enables the deposition of HA bioink layer by layer after being methacrylated^[^ ^389^, ^390^ ^]^ or thiolated^[^ ^391^ ^]^ during additive manufacturing while maintaining structural integrity and fidelity.

Notably, HA functions to absorb shock and lubricate joints, contributing to the alleviation of knee pain and the improvement of joint mobility in individuals with osteoarthritis following intra‐articular injections of HA.^[^ ^384^ ^]^ Interestingly, the biological activity of HA is heavily influenced by its molecular weight, with high molecular weight HA exhibiting anti‐inflammatory properties and inhibiting cell movement,^[^ ^392^ ^]^ while low molecular weight HA enhances cell motility and may promote inflammation.^[^ ^393^, ^394^, ^395^ ^]^ HA‐based bioinks offer tunable mechanical properties, allowing researchers to tailor the stiffness and viscoelasticity of printed constructs to match specific tissue requirements. Moreover, HA hydrogels demonstrate good thermal stability, allowing for processing under mild conditions compatible with cell viability and bioactivity.

Due to its biocompatibility and biodegradability, HA has gained significant attention in various biomedical applications, including tissue engineering, drug delivery, and wound healing. In terms of biocompatibility, HA is naturally present in the human body and is well tolerated without eliciting significant immune responses or cytotoxic effects. Its biodegradability is mediated by enzymatic degradation by hyaluronidases, which cleave the glycosidic bonds in HA molecules. This controlled degradation process ensures the safe resorption of HA hydrogels over time, making them suitable for temporary scaffolds in tissue engineering applications. Commercial products based on HA include dermal fillers for cosmetic augmentation, ophthalmic viscosurgical devices for eye surgery, and drug delivery formulations for intra‐articular injections. Overall, the versatile properties of HA make it a promising biomaterial for additive manufacturing and various biomedical applications.

#### 3.3.9 Polyhydroxyalkanoates (PHAs)

PHAs represent a class of biopolyesters synthesized by numerous microorganisms as intracellular carbon and energy storage compounds. While some researchers categorize it as a synthetic biodegradable polymer due to its relatively simple molecular structure as an aliphatic polyester,^[^ ^211^ ^]^ it is often viewed as natural since it is produced by blue‐green algae, estuarine micro‐flora, and soil bacteria. They are composed of hydroxyalkanoic acid monomers and vary in chain length, degree of crystallinity, and mechanical properties depending on the microbial strain and fermentation conditions. PHAs have garnered significant attention in additive manufacturing due to their biodegradability, biocompatibility, and thermoplastic behavior.^[^ ^396^, ^397^ ^]^ With the rising demand for sustainable materials, PHAs offer a promising alternative to conventional petroleum‐based plastics in various industrial applications. Also note that PHB is one of the most well‐known and extensively studied members of the PHA family. It is a homopolymer consisting solely of 3‐hydroxybutyrate monomers.^[^ ^398^ ^]^ To tailor PHB properties, the copolymer poly(3‐hydroxybutyrate‐co‐3‐hydroxyvalerate) (PHBV) is commonly synthesized for large‐scale production, extending its applications to sutures, specialty packaging, orthopedic devices, and more.^[^ ^399^ ^]^ With a melting point of 180 °C,^[^ ^400^ ^]^ PHB is compatible with additive manufacturing techniques like FDM and SLS.^[^ ^401^, ^402^ ^]^

The mechanical and thermal properties of PHAs can be tailored by adjusting the monomer composition, molecular weight, and processing conditions. Generally, PHAs exhibit mechanical properties comparable to traditional petroleum‐based plastics, with tensile strength, modulus, and elongation at break varying depending on the specific PHA type. Additionally, PHAs possess good thermal stability, with melting temperatures typically ranging from 40 to 180 °C, making them suitable for processing via various additive manufacturing techniques. Their thermoplastic behavior allows PHAs to be processed into complex three‐dimensional structures using methods such as FDM, SLS, or SLA.

PHAs are renowned for their excellent biocompatibility and biodegradability, making them highly suitable for medical and biomedical applications. These biopolymers degrade naturally in various environments, including soil, water, and biological systems, without leaving behind harmful residues. In biomedical fields, PHAs have been utilized in tissue engineering, drug delivery systems, surgical implants, and wound dressings. Their compatibility with living tissues and ability to degrade into non‐toxic byproducts ensure minimal adverse effects on the body. Commercially, PHAs have seen growing use in packaging materials, disposable items, and agricultural applications, driven by their renewable nature and environmental benefits.

#### 3.3.10 Silk

Silk, a natural protein fiber produced by silkworms and spiders, spun by certain Arachnida species and various Lepidoptera larvae.^[^ ^403^, ^404^ ^]^ It is composed mainly of fibroin, a structural protein, along with sericin, a sticky protein coating. The remarkable mechanical properties of silk, including its tensile strength, elasticity, and toughness, make it an attractive material for various engineering and biomedical applications.^[^ ^405^ ^]^ Processing‐wise, silk can be modified to immobilize growth factors,^[^ ^406^, ^407^, ^408^ ^]^ leveraging its amino acid side chains for this purpose.^[^ ^403^ ^]^ Its versatility enables manufacturing using extrusion‐based printing,^[^ ^409^ ^]^ binder jetting‐based printing,^[^ ^188^ ^]^ and DLP‐based printing methods.^[^ ^77^, ^410^ ^]^ Notably, introducing methacrylate through substitution reactions, which replace the amino groups, facilitates rapid crosslinking of silk initiated by UV light, greatly enhancing the feasibility of additive manufacturing using silk‐based bioinks.^[^ ^77^, ^410^ ^]^

In terms of physical properties, the predominantly hydrophobic nature of silk proteins repels water, leading to increased packing density and the formation of β‐crystallinity, both of which contribute to its exceptional mechanical properties. Besides, silk possesses exceptional mechanical toughness and flexibility, rivaling that of many synthetic materials. Its high tensile strength, coupled with its elasticity, enables silk to withstand considerable stress and deformation without breaking. Additionally, silk exhibits excellent thermal stability, retaining its mechanical properties at high temperatures, which is advantageous for manufacturing processes involving heat. Furthermore, silk fibers are lightweight and possess a natural luster, making them aesthetically pleasing and suitable for a wide range of applications, including textiles, medical devices, and consumer products.

The biocompatibility and biodegradability of silk make it particularly well‐suited for biomedical applications. For example, silk exhibits excellent thermal stability, biocompatibility, and biodegradability, further enhancing its suitability for use in additive manufacturing processes. Its biocompatibility allows for direct interaction with biological systems, making it ideal for tissue engineering scaffolds,^[^ ^411^, ^412^ ^]^ drug delivery systems,^[^ ^413^, ^414^, ^415^ ^]^ artificial blood vessels,^[^ ^416^ ^]^ and wound dressings.^[^ ^417^ ^]^ Silk‐based materials have been extensively studied for their potential use in tissue engineering, where they can serve as scaffolds to support cell growth and tissue regeneration. Silk biomaterials have also been explored for drug delivery systems, wound healing dressings, and surgical implants due to their ability to degrade in vivo without eliciting harmful immune responses. Commercially, silk‐based medical devices and implants have been developed for various applications, including sutures, meshes for hernia repair, and scaffolds for tissue engineering, highlighting the versatility and utility of silk in the biomedical field.

#### 3.3.11 Sodium Alginate

Alginate is a naturally occurring polysaccharide extracted from brown algae, primarily composed of linear chains of mannuronic and guluronic acid residues.^[^ ^41^ ^]^ Chemically, alginate comprises linear copolymers consisting of (1,4)‐linked β‐D‐mannuronate (M) and α‐L‐guluronate (G) units, with the ratio between these units varying depending on the seaweed species.^[^ ^418^ ^]^ Alginate can be conventionally processed through techniques such as gelation, extrusion, and molding. One common method involves dissolving alginate powder in a solvent, typically water, to form a viscous solution. The solution is then poured or injected into molds of desired shapes and sizes, where it undergoes gelation induced by the addition of divalent cations such as calcium ions. This gelation process crosslinks the alginate chains, transforming the solution into a solid hydrogel with the desired structure. Alternatively, alginate solutions can be extruded through nozzles or syringes to form filaments or three‐dimensional structures, which are subsequently immersed in a calcium chloride solution to induce gelation. Molding techniques can also be employed to shape alginate hydrogels by pouring the alginate solution into molds and allowing it to gel. Widely utilized in additive manufacturing, alginate is favored for its ability to form hydrogels through ionic crosslinking with divalent cations like calcium ions (Ca2+).^[^ ^419^, ^420^ ^]^ This property makes alginate an attractive material for biofabrication applications, particularly in tissue engineering and regenerative medicine, where it can serve as a scaffold for cell growth and tissue regeneration.^[^ ^421^ ^]^ Additive manufacturing techniques, such as light‐curing‐based SLA or DLP and extrusion‐based bioprinting or DIW have been employed to fabricate complex three‐dimensional structures using alginate‐based bioinks. For example, the G‐blocks within alginate molecules interact with divalent cations during bioprinting‐based gelation, forming cross‐links and creating junctions between adjacent polymer chains through an egg‐box structure.

In terms of physical properties, alginate hydrogels exhibit excellent biocompatibility and are biodegradable in vivo. These hydrogels possess high water content, resembling the native ECM, which promotes cell adhesion, proliferation, and differentiation. The mechanical properties of alginate hydrogels can be tuned by adjusting parameters such as polymer concentration, crosslinking density, and the addition of reinforcing materials. While alginate hydrogels typically have low mechanical strength, they can be reinforced with nanoparticles or other polymers to enhance their mechanical properties for specific applications. Additionally, alginate hydrogels demonstrate good thermal stability, retaining their structure and properties under physiological conditions.

In the field of biocompatibility, alginate‐based hydrogels have been extensively investigated for various biomedical applications due to their non‐toxic nature and ability to support cell growth and tissue regeneration. While alginate excels at forming 3D structures, it lacks mammalian cell receptors, resulting in relatively low protein adsorption.^[^ ^419^ ^]^ Consequently, researchers often modify alginate with RGD peptides to enhance cell adhesion and proliferation.^[^ ^422^, ^423^, ^424^ ^]^ Alginate hydrogels are known to degrade gradually over time through hydrolysis of the glycosidic bonds between the alginate chains. This controlled degradation allows for the gradual release of encapsulated bioactive molecules and cells, making alginate an attractive material for drug delivery systems and tissue engineering scaffolds (e.g., bones,^[^ ^42^ ^]^ cartilages,^[^ ^425^ ^]^ nerves,^[^ ^426^ ^]^ and internal organs^[^ ^427^, ^428^ ^]^). Commercial products utilizing alginate‐based hydrogels include wound dressings, cell encapsulation systems, and injectable hydrogel formulations for tissue regeneration.^[^ ^43^ ^]^ For example, its rapid sol‐gel transition makes it suitable for coextrusion into concentric grafts used as biomedical conduits, such as artificial blood vessels.^[^ ^429^, ^430^, ^431^ ^]^

#### 3.3.12 Xylan

Xylans, a class of semicelluloses, is the second abundant biopolymer in the whole plant kingdom. It can be found in wood, grasses, cereals, and herbs.^[^ ^432^ ^]^ For example, Xylans account for 25–35 wt% in dry woody tissues of dicots and lignified tissues of monocots. In some cereal grain tissues, the weight percentage can reach up to 50%.^[^ ^432^ ^]^ Specifically, xylan primarily exists in the secondary cell wall, defending against herbivores and pathogens, making it essential for plant growth.^[^ ^433^ ^]^ Consisting of a backbone of xylose units, xylan is a linear polysaccharide comprised of β‐(1,4)‐linked xylose residues substituted with acetyl, glucuronic acid (GlcA), 4‐Ο‐methylglucuronic acid (Me‐GlcA), and arabinose residues.^[^ ^433^ ^]^ The structures can vary depending on different sources. Beyond applications in papermaking, baking, and food additives,^[^ ^434^ ^]^ xylans have been reported to be utilized as the feedstock for DIW due to their viscoelastic behavior.^[^ ^435^ ^]^

Hydrogen bonding and covalent bonds are present in the interactions between xylan and other polymers in native plant tissues.^[^ ^436^, ^437^ ^]^ Yet, chemical and enzymatic modifications might be necessary to tune its mechanical properties and expand its applications. For instance, xylan can be functionalized with tyramine to be enzymic crosslinked during DIW printing.^[^ ^198^ ^]^ Although its tensile strength and Young's modulus are not outstanding, reinforcement fillers can be included due to its compatibility with other materials, allowing the production of products such as high‐strength films.^[^ ^438^ ^]^

The enzymes that degrade xylan are only found in the human digestive system, produced by certain microorganisms present in the gut microbiota.^[^ ^439^ ^]^ In medical and pharmaceutical applications, it is reported to have antioxidant and anti‐tumor activity.^[^ ^440^ ^]^ After modification, sulfated xylans can be used as biologically active components in drugs as well.^[^ ^441^ ^]^ Other biomedical related fields that xylan covers include hydrogel tissue engineering, drug delivery, wound healing, medicine encapsulation, and mucoadhesive systems.

#### 3.3.13 Other Nature Polymers with Limited 3D Printing Explorations

As products of chemical reactions, nearly unlimited types of natural polymers can be found in various organisms. Most of the natural polymers used as 3D printing materials serve as supports and membrane structures for living organisms due to their relatively robust mechanical properties and the potential to form gels. As an example for the other side, polynucleotides (e.g., ribonucleic acid (RNA) and deoxyribonucleic acid (DNA)) predominantly functions as the repository of genetic information, directing cellular processes and orchestrating the intricate workings of cells.^[^ ^442^ ^]^ Therefore, few projects have been reported to use them as feedstocks for 3D printing. Similarly, polymers related to energy storage, such as glycogen, a type of storage polysaccharide, have not garnered attention in the 3D printing industry. Yet, some natural polymers might fill the niche market for certain 3D‐printed biomedical applications. For instance, silk sericin, a by‐product of silk fibroin, possesses gel‐forming properties; cutin, the waxy layer secreted by plants, has the potential to enhance the water retention capacity of printed parts; scleroglucan, a polysaccharide produced by fungi, finds application in pharmaceuticals.^[^ ^443^ ^]^ With the continuous development of additive manufacturing techniques and biotechnology, an increasing number of biopolymers will inevitably become available for 3D printing.

### 3.4 Human Cells and Tissues

#### 3.4.1 General Human Cells and Tissues for Regenerative Medicine in 3D Printing

Regenerative medicine is a rapidly advancing field with the potential to restore or replace damaged tissues and organs by utilizing the body's own cells and tissues for repair and regeneration.^[^ ^444^ ^]^ The core of regenerative medicine lies in the use of human cells and tissues, which act as fundamental building blocks for engineering functional tissues. The diversity of human cells used in regenerative medicine is vast, ranging from stem cells to differentiated tissue‐specific cells. The most commonly used cells include mesenchymal stem cells (MSCs),^[^ ^445^ ^]^ which can be derived from sources such as bone marrow, adipose tissue, and umbilical cord blood. These MSCs have a well‐documented capacity to differentiate into multiple cell types, including osteocytes, chondrocytes, and adipocytes, making them especially valuable for applications in musculoskeletal tissue engineering and cell‐based therapies. For example, as the major stem cells for cell therapy, MSCs have been used clinically for ∼20 years for treating tissue injuries and immune disorders.^[^ ^446^ ^]^ The induced pluripotent stem cells (iPSCs) also play a key role in regenerative medicine, offering the unique ability to differentiate into almost any cell type (e.g., neurons,^[^ ^447^ ^]^ cardiomyocytes,^[^ ^448^ ^]^ or pancreatic cells^[^ ^449^ ^]^) in the body. Unlike MSCs, iPSCs are often derived from adult somatic cells reprogrammed to a pluripotent state, providing a renewable source of patient‐specific cells for tissue engineering.

Stem cells are not the only type of cells used in regenerative medicine. Embryonic stem cells (ESCs) hold significant potential because of their ability to proliferate indefinitely and differentiate into all three germ layers, offering possibilities for a wide range of regenerative applications.^[^ ^450^ ^]^ However, ethical concerns and immunogenicity have limited the widespread clinical use of ESCs,^[^ ^451^ ^]^ placing more emphasis on adult stem cells and iPSCs. These advancements in cellular biology have paved the way for tissue engineering and the development of more complex tissues. In particular, 3D printing technologies have revolutionized the field of regenerative medicine by enabling the precise arrangement of cells and biomaterials to create tissue scaffolds that mimic the extracellular matrix (ECM).^[^ ^452^ ^]^ Scaffolds provide structural support and act as a framework for the proliferation and differentiation of cells, thus facilitating the formation of functional tissues.

In recent years, 3D printing has been used to biofabricate human cells and tissues not only skin grafts,^[^ ^453^ ^]^ but also more complex tissues such as cartilage,^[^ ^454^ ^]^ bone, and even organs, including the bladder.^[^ ^455^ ^]^ These biofabricated constructs are enriched with bioactive molecules such as growth factors and cytokines^[^ ^456^ ^]^ to promote cellular functions such as proliferation, differentiation, and migration. For example, transforming growth factor‐beta (TGF‐β) is a crucial signaling molecule used in tissue engineering to promote mesenchymal cell proliferation and facilitate immunomodulatory effects.^[^ ^457^ ^]^ Similarly, vascular endothelial growth factor (VEGF) plays a pivotal role in angiogenesis, an essential process in creating vascular networks for engineered tissues.^[^ ^458^ ^]^ With advances in bioactive scaffold fabrication, including nanofiber scaffolds and hydrogels, 3D printing allows the creation of complex, multi‐layered tissues that more closely resemble the hierarchical architecture of native tissues.

The potential applications of 3D printing human cells and tissues in regenerative medicine are wide‐ranging, including the development of personalized tissue grafts,^[^ ^459^ ^]^ organoids for disease modeling,^[^ ^460^ ^]^ and biomaterial‐based drug delivery systems.^[^ ^92^ ^]^ These approaches not only provide solutions for the current shortage of organ donors but also offer a way to create personalized treatments that are tailored to individual patients. As the field progresses, further research is expected to focus on overcoming current challenges such as biomaterial biocompatibility, vascularization, and immune response. Though without an issue being biocompatible when these biomaterials are extracted from human beings or animals,^[^ ^461^ ^]^ vascularization remains a critical hurdle in tissue engineering,^[^ ^462^ ^]^ particularly for larger tissue constructs like liver and heart tissue,^[^ ^463^, ^464^ ^]^ where functional blood vessels are essential for supplying oxygen and nutrients to cells. Innovations in bioactive scaffolds incorporating pro‐angiogenic factors such as VEGF have shown promise in promoting vascular networks within engineered tissues.^[^ ^465^ ^]^ Additionally, addressing the immune response has led to the development of immunomodulatory biomaterials that reduce inflammation and prevent immune rejection,^[^ ^466^ ^]^ which is crucial for applications like skin grafts and organ transplants, where patient‐specific engineered tissues are vital for avoiding immunological complications. In conclusion, the integration of human cells and tissues into 3D printing for regenerative medicine opens new frontiers in creating functional tissues and organs, ultimately improving patient outcomes and addressing critical healthcare needs worldwide.

#### 3.4.2 3D Printing of Human Cells and Tissues Processing

3D printing has emerged as a transformative technology in regenerative medicine, offering the potential to create complex, patient‐specific tissue constructs with unprecedented precision.^[^ ^467^ ^]^ This innovative approach, often referred to as bioprinting, utilizes bioinks composed of living cells and supportive biomaterials to deposit layer upon layer and form three‐dimensional structures that closely mimic native tissues. Bioprinting techniques, including extrusion‐based, droplet‐based, and laser‐assisted methods, enable the fabrication of tissues with intricate architectures, such as blood vessels and cellular gradients,^[^ ^429^ ^]^ which are essential for proper function. The marriage of 3D printing and regenerative medicine opens up new frontiers for tissue transplantation and personalized medical treatments. Researchers are exploring bioprinting for the generation of various tissues, including neurons,^[^ ^468^ ^]^ cartilage, bone,^[^ ^469^ ^]^ and even organs such as liver and heart patches.^[^ ^470^, ^471^ ^]^ Also, the bioprinting of cellular gradients has become increasingly critical in replicating tissue interfaces such as cartilage‐to‐bone transitions, ensuring that the construct mimics the natural structural and mechanical properties of tissues at these critical junctions.

3D bioprinting has been instrumental in advancing research in tissue transplantation and personalized medical treatments.^[^ ^472^ ^]^ In the field of cartilage and bone regeneration, studies have demonstrated the successful fabrication of cartilage‐bone constructs using multi‐material bioprinting technologies.^[^ ^473^ ^]^ For instance, hydrogel bioinks reinforced with nanoparticles such as graphene oxide and other human tissues can be used to improve the mechanical properties of bioprinted cartilage tissues, leading to greater functional longevity in implants.^[^ ^474^ ^]^ This intersection of materials science and biology shows how the combination of different bioinks and printing strategies allows researchers to create tissue constructs with tunable mechanical and biological properties. Bioprinting has also been applied in creating neural tissue constructs to investigate nerve repair and regeneration.^[^ ^475^ ^]^ For instance, researchers are developing 3D‐printed scaffolds seeded with neural stem cells, providing a pathway for nerve regeneration in patients with spinal cord injuries.^[^ ^476^ ^]^

Despite the remarkable advances in bioprinting, several technical and biological challenges remain, particularly in ensuring cell viability during printing,^[^ ^296^ ^]^ achieving vascularization for nutrient supply,^[^ ^477^ ^]^ and establishing the long‐term functionality of tissues.^[^ ^478^ ^]^ During the printing process, cells are often subjected to mechanical stresses and exposure to light (in photocurable bioinks), which may reduce their viability or alter their functionality. Methods such as low‐temperature bioprinting and encapsulation of cells in hydrogels have been explored to mitigate these risks and enhance post‐printing cell survival.^[^ ^296^ ^]^ Achieving vascularization, which is essential for the survival and functionality of thicker tissues, is another significant hurdle. While some progress has been made in printing rudimentary vascular structures, ensuring their functional integration into host tissues and supporting long‐term blood flow remains a challenge. Advanced bioprinting techniques, such as co‐printing endothelial cells with scaffolding materials,^[^ ^479^ ^]^ show promise in addressing this limitation by facilitating the formation of capillaries and vascular networks within bioprinted constructs.

Additionally, the precision offered by 3D bioprinting allows for the development of patient‐specific tissues and organs, providing personalized medical treatments that improve patient outcomes.^[^ ^472^ ^]^ The ability to precisely control the spatial arrangement of cells and biomaterials allows for the creation of tissue constructs that closely resemble native tissues in structure and function. For instance, Choi et al. conducted 3D cell printing with aligned cell constructs, facilitating myotube formation, differentiation, and maturation.^[^ ^480^ ^]^ With imaging technologies like computed tomography (CT)^[^ ^481^ ^]^ and magnetic resonance imaging (MRI), personalized anatomical data can be utilized to generate highly accurate 3D models for bioprinting. These patient‐specific models are particularly valuable in applications such as bone reconstruction or cartilage replacement,^[^ ^473^ ^]^ where anatomical accuracy is crucial for the functionality of the implant. Moreover, the flexibility of 3D bioprinting enables the customization of tissue constructs to match the individual patient's needs,^[^ ^482^ ^]^ ensuring that the bioprinted tissues integrate seamlessly with the host body. For example, including bioactive molecules in the bioprinting process enables the creation of dynamic tissue constructs that can respond to environmental stimuli and adapt to changing conditions, making them more suitable for long‐term implantation and functional restoration of damaged tissues.^[^ ^17^ ^]^ 3D printing provides a novel approach to manipulating biomaterials and bioactive molecules. Although it is not the focus of this review, we foresee that as research in this field continues to advance, 3D printing holds the potential to revolutionize regenerative medicine by offering novel solutions for tissue repair and replacement in both clinical and experimental settings.

## 4 3D Printable Polymers for Biomedical Applications

### 4.1 Sensors for Health Monitoring

#### 4.1.1 Resistive/Conductive Sensors

**Figure 4:**
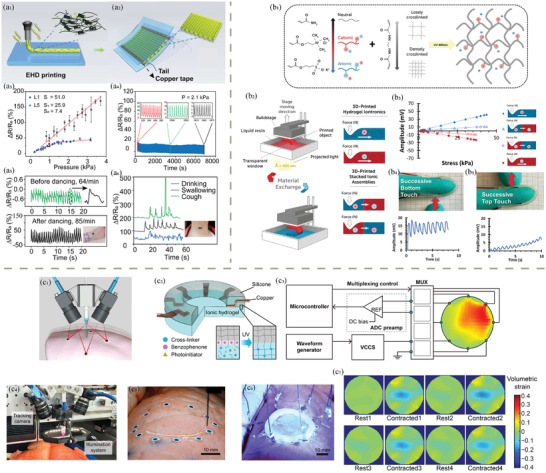
a_1_) EHD jetting of the sensing layer (PEO/PANI/Graphene). a_2_) Structure of the packaged sandwiched sensor. a_3_) Resistance response of the printed pressure sensor under various pressures (with 1 jetted layer and 5 jetted layers). a_4_) Resistance response of the sensor under 1000 loading‐unloading cycles at a frequency of 0.15 Hz. a_5_) Recorded pulse wave curves before and after dancing by the sensor. a_6_) Recorded resistance signals of cough, swallowing, and drinking. Reproduced with permission.^[^ ^489^ ^]^ Copyright 2022, ACS. b_1_) Chemical sketch of copolymerization of acrylamide (Aam) with [2‐(acryloyloxy) ethyl] trimethylammonium chloride (AETA) or 3‐sulfopropyl acrylate potassium salt (SPA) using N, N’‐methylenebisacrylamide (MBA) crosslinker, which was used in the SLA printing. b_2_) SLA printing of stacked ionic assemblies via resin vat exchange during the 3D printing process, where the ion type, charge density, and crosslinking density within the iontronic device can vary. b_3_) Output voltage amplitude as a function of the applied stress, with the compartments having different ion types of SPA versus AETA at 30 mol.%. A 3D‐printed two‐compartmented finger sleeve with tactile feedback mimicking successive touches on the fingertip (b_4_) and the fingernail (b_5_) showing different sensitivities. Reproduced with permission.^[^ ^490^ ^]^ Copyright 2023, Wiley. c_1_) Schematic image of in situ 3D printing of DIW on a breathing lung and real‐time tracking of the surface. c_2_) Layered structure of the hydrogel‐based electrical impedance tomography (EIT) sensor, with the inset image showing the silicone‐hydrogel interface bonding that enhances the robustness of the device. c_3_) Schematic of the peripheral operating circuitry for the sensing system. MUX, multiplexing; DC, direct current. c_4_) Photo of the in‐situ printing gantry system. c_5_) Photo of the printed circular layer of the hydrogel and the fiducial markers on the surface of the lung. c_6_) UV‐curing of the silicone ring to fix the embedded electrodes. c_7_) Results obtained via the spatiotemporal mapping of a porcine lung in two cyclic contractions. Reproduced with permission.^[^ ^492^ ^]^ Copyright 2020, AAAS.

Sensors play a crucial role in human health by enabling continuous, real‐time monitoring of various biological and physical parameters. Unlike traditional intermittent analytical technologies, biosensors provide ongoing data, which is essential for timely and accurate health assessments.^[^ ^483^ ^]^ This continuous monitoring capability is particularly important in managing chronic diseases, tracking vital signs, and providing immediate feedback on physiological changes, thereby enhancing the quality of life and potentially extending life expectancy.^[^ ^484^ ^]^ The advent of 3D printing technology has significantly enhanced the design and manufacturing of sensors. 3D printing allows for unprecedented customizability, enabling the creation of sensors that are tailored to the specific needs of individual patients.^[^ ^485^ ^]^ This technology supports the miniaturization of sensors, making them less invasive and more comfortable for long‐term use. Additionally, 3D printing facilitates the integration of complex sensor architectures and multifunctional materials, leading to the development of more sophisticated and efficient biosensors. To detect electrical signals, the most prevalent approach is to add a conductive phase to polymer matrices since electrical conductivity provides biomaterials with the capabilities to serve as bioactuators and biosensors.^[^ ^486^, ^487^, ^488^ ^]^ For instance, Song et al. employed a modified electrohydrodynamic (EHD) jetting method to manufacture a sandwich‐structured flexible pressure sensor (**Figure** **4**a_2_). This sensor mimics environmental conditions (Figure 4a_3_,a_4_) and monitors various physiological parameters(Figure 4a_5_,4a_6_).^[^ ^489^ ^]^ The sensing ability originated from the graphene‐polyaniline (PANI)‐embedded PEO mesh, which was EHD printed, showing linear and rapid response in resistance observed under external pressure (Figure 4a_3,4_). By recording the resistance change, physiological signals, such as changes in pulse rate in different exercise states (i.e., drinking, swallowing, and coughing, Figure 4a_5_) and movements of the throat during different activities (Figure 4a_6_), could be identified. This innovation demonstrates how 3D printing can enhance sensor functionality and responsiveness, crucial for health monitoring applications. Besides electrical conductors, ionic conductors can also sense the mechanical forces and movements of organisms. As demonstrated in Figure 4b, Raquez's team delved into iontronics, taking advantage of the piezoionic effect that a voltage will be generated regarding the flow of charges from the point of compression, and developed a touch sensor.^[^ ^490^ ^]^ The ionic hydrogel consists of polyacrylamide (PAAm) which can be processed with SLA (Figure 4b_1_). By adding ionic co‐monomers to the resin vat (Figure 4b_2_), mobile counterions were introduced into the hydrogel, endowing the material with ionic conductivity. The sensitivity is influenced by ion concentrations, cross‐link densities, and oppositely charged regions (Figure 4b_3_). A two‐compartment sensor was printed to mimic finger tactile sensation with different sensitivities at the bottom and the top of the fingers (Figure 4b_4_,b_5_). The electromechanical gradients were controlled by exchanging the resin formulation with separate reservoirs.

Both the aforementioned electrically conductive and ionically conductive sensors operate on the surface of human skin, where the criteria for biocompatibility are less stringent. In these applications, the primary concern is often limited to avoiding skin irritation or allergic reactions, which can be relatively easily managed with appropriate material choices. However, when these sensors are intended for use with internal tissues or organs, the requirements for biocompatibility become significantly more rigorous. In such cases, the biocompatibility is critically dependent on the interactions between the sensor materials and the cells, tissues, or interior organs within the body. These interactions can influence the body's immune response, the potential for inflammation, and the integration of the sensor with the biological environment.^[^ ^491^ ^]^ As a trial, Zhu et al. advanced the sensor from the skin surface to the organ surface, an in vivo environment (Figure 4c). The authors leveraged an ionic hydrogel to obtain continuous spatial mapping of lung deformation and combined it with a DIW technique that can adapt to moving biological surfaces.^[^ ^492^ ^]^ Upon dissolving lithium chloride in the PAAm matrix, the ionic hydrogel was printed as a thin sensing layer, exhibiting conductivity change with deformation (Figure 4c_1_‐c_2_). The distribution of sheet conductivity was extracted by the surrounding electrodes, which was then translated into spatiotemporal mapping results of lung deformation (Figure 4c_3_). The printing process on the lung surface took place under dynamic conditions, mimicking the respiratory cycles (Figure 4c_4_‐c_6_). The adaptive 3D printing system analyzed movement of the target surface, estimating the deformation in the form of a dynamic point cloud, and allowed for control of printhead for the dynamic surface of the lung (Figure 4c_7_). While this project holds significant engineering importance in the context of printing biosensors directly onto living organs, further in vivo experiments are needed to assess the long‐term biocompatibility of the printed material system, owing to the potential toxicity associated with SLA feedstocks.

#### 4.1.2 Capacitive Sensors

**Figure 5:**
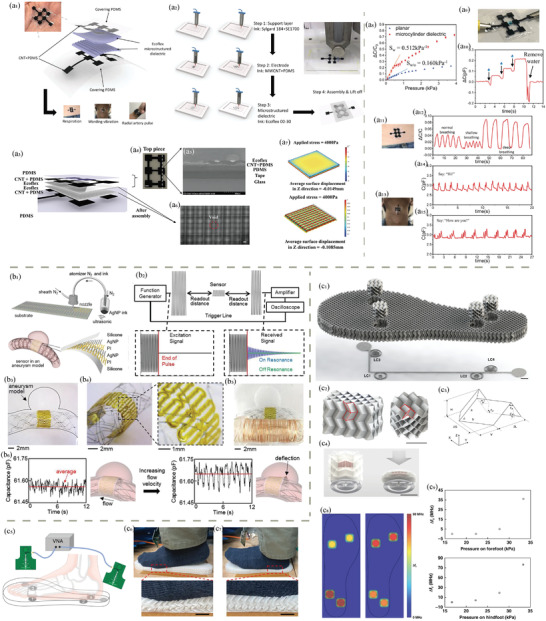
a_1_) Photograph and schematic of the multimaterial all‐3D‐printed nanocomposite‐based (M2A3DNC) capacitive pressure sensor for multiple physiological signals monitoring. a_2_) Schematic representation of 3D printing of the PDMS supporting layer, the MWCNT+CNT conductive layer, and the Ecoflex‐microstructured dielectric layer in 3 steps, followed by manual assembly of two printed pieces and exfoliation from the precoated PVA in a water bath. Inset: optical image of 3D printing of the hemicylinder‐patterned dielectric layer. a_3_) Layer schematics of the M2A3DNC sensor. a_4_) Optical image of the printed dielectric layer on top of the supporting and conductive layer, scale bar: 2 mm. a_5_) The cross‐sectional SEM image of the 3D‐printed multilayers on a double‐sided tape attached to a glass substrate. a_6_) The microscopic image of the dielectric grids, scale bar 100 µm. a_7_) Simulated Z‐axis displacement under 4 kPa for the (top) planar structured and the (bottom) microcylinder structured dielectric. a_8_) The capacitance changes under different pressures for the M2A3DNC sensors with and without a microstructure‐patterned dielectric layer. (a_9_) The photograph of the M2A3DNC sensor measuring the pressure of a water droplet (≈9 Pa), and a_10_) the recorded pressure of three sequentially applied water droplets. a_11_) The photograph of the capacitive sensor on a human abdomen, measuring a_12_) the breathing waves with different breathing tempos. a_13_) Optical image of the sensor on a human's throat, recording the acoustic vibrations of the larynx when saying a_14_) “Hi” and a_15_) “How are you”. Reproduced with permission.^[^ ^494^ ^]^ Copyright 2021, Wiley. b_1_) Schematic image of aerosol jet printing of the Ag electrode layer with an ultrasonic atomizer and a multilayer capacitance sensor implanted in an aneurysm model. b_2_) Circuit overview and example of received wireless signals in on‐ and off‐resonance cases. Photographs of b_3_) a zoomed‐out overview of the sensor, b_4_) zoomed‐in views of the capacitive sensor, b_5_) the sensor with a copper coil for wireless monitoring of hemodynamics in an in vitro study. b_6_) Pulsatile flow captured by the sensor, where the average capacitance increased with flow velocities from 0.05 (left) to 0.35 (right) m s^−1^. The schematic demonstrates that the increased capacitance resulted from the increased blood flow into the aneurysm. Reproduced with permission.^[^ ^500^ ^]^ Copyright 2019, Wiley. c_1_) Schematic of the insole with four inductor‐capacitor (LC) sensors positioned beneath cylindrical origami blocks 3D printed via the FDM method. c_2_) Descriptive schematics showing different directions of origami for the (left) overall insole part and the (right) cylindrical pressure sensing part. c_3_) The Miura‐ori foldcore unit. c_4_) 3D images demonstrate the deformation of the origami block with conductive serpentines (pink) under pressure. c_5_) Experiment setup of the sensor‐embedded insole and two antennas (green) connected to the vector network analyzer (VNA). Photographs of the deformed insole with pressure (c_6_) on the forefoot and the (c_7_) hindfoot. c_8_) Pressure mapping of two postures with a higher pressure applied to the hindfoot than the forefoot (left) and a higher pressure applied to the forefoot than the hindfoot (right). c_9_) Resonant frequency changes (Δf_r_) regarding the pressure applied on (top) forefoot position and (bottom) hindfoot position (scale bars: 10 mm). Reproduced with permission.^[^ ^502^ ^]^ Copyright 2022, Springer Nature.

In addition to resistive sensors, capacitive sensors offer another method for detecting mechanical signals from organs, alongside the conductors previously mentioned. Capacitive sensors operate on the principle of detecting changes in capacitance, which can be influenced by mechanical deformations, pressure, or proximity of objects.^[^ ^493^ ^]^ This capability makes them highly effective for applications that require precise measurement of physical changes, such as monitoring physiological movements and detecting subtle pressure variations. 3D printing can contribute to the manufacturing flexibility of their multilayered structures. Yi et al. delved into the structure of the flexible dielectric layer, whose deformation alters the capacitance change of capacitive sensors (**Figure** **5**a_1_‐5a_4_).^[^ ^494^ ^]^ Their team utilized DIW to fabricate hemicylinder microstructures of Ecoflex, a flexible aliphatic‐aromatic copolyester, for use as the dielectric layer (Figure 5a_5_,a_6_). The microstructure enhanced material deformation under equivalent pressures compared to a common planar design (Figure 5a_7_,a_8_), thus enabling the sensor to detect subtle pressures such as water droplets (Figure 5a_9_,a_10_). As shown in Figure 5a_2_, the support layer and the conductive electrode ink were 3D‐printed as well, and the efficiently assembled sensor was capable of recording different breathing modes (Figure 5a_11_,5a_12_) and differentiating simple words from throat vibrations (Figure 5a_13_‐a_15_). Although the application of this sensor is currently limited to in vitro environments, research indicates that aliphatic‐aromatic copolyesters have the potential to degrade with low toxicity.^[^ ^495^ ^]^ This suggests that there may be approaches to render the capacitive sensor degradable by selecting alternative materials for the PDMS/CNT layer. The toxicity of CNT in particular is controversial,^[^ ^496^ ^]^ and caution is warranted when applying them in vivo studies.

One pivotal advantage of capacitive sensors is their capability to act as inductor‐capacitor (LC) passive wireless sensors, an idea proposed as early as 1967.^[^ ^497^ ^]^ Essentially, a sensing capacitor is connected to a spiral inductor, forming a resonant LC tank. A readout coil is remotely coupled with the LC tank, monitoring its impedance. The capacitor change is thus reflected in a shift in the resonant frequency of the LC tank.^[^ ^498^ ^]^ The system is suitable for medical sensing applications,^[^ ^499^ ^]^ because physical connections and batteries are unnecessary.^[^ ^498^ ^]^ For instance, capacitive sensors can be used to monitor internal organ functions or measure biomechanical signals from soft tissues without invasive wiring,^[^ ^499^ ^]^ thereby reducing the risk of infection and improving patient comfort. Herbert et al. conducted aerosol jet printing (AJP) to produce a flexible and stretchable sensor, with polyimide as the dielectric layer and silver nanoparticle composite as the capacitive electrodes (Figure 5b_1_).^[^ ^500^ ^]^ With variations in the applied pressure and deflection, the thickness of the dielectric layer would change, causing the capacitance to change correspondingly. As shown in Figure 5b_2_, the transient capacitance signals could be captured wirelessly due to the inductive coupling principles between the sensor and two external coils, resulting in a resonance shift associated with the capacitance change. Moreover, the serpentine structure produced by the AJP was able to fit in contoured neurovessels (Figure 5b_3_–b_5_), monitoring hemodynamics in the brain by sensing increased flow into aneurysms (Figure 5b_6_). Given the in vivo operating environment, the primary focus of this idea is likely to evaluate biocompatibility. Although polyimide has demonstrated cell viability and proliferation in various common cell types, further systematic studies are necessary to assess its potential for clinical use.^[^ ^501^ ^]^

Apart from aerosol jet or inkjet printing as mentioned above, the FDM method as a simple processing technique can also tune material microstructures for sensing applications. For example, Kim et al. focused their effort on in vitro biomechanical signals by developing an origami insole with wireless pressure sensors (Figure 5c).^[^ ^502^ ^]^ The origami structure was fabricated by FDM using TPU as the filament, while the conductive part of the circuit was printed by DIW using a silver particle‐embedded acetate as the feedstock(Figure 5c_1_). One advantage of the origami structure was its ability to adjust the compressibility by tuning the parameters of its foldcore (Figure 5c_3_), matching users’ weight. Moreover, changing the orientation of the same origami structure could also reduce the compressibility of the insole above the LC sensors (Figure 5c_2_–c_4_), enhancing their sensitivity. As the origami blocks were compressed, the gap between the LC sensors and the serpentines varied (Figure 5c_5_–c_8_), causing a change in the resonant frequency (Figure 5c_9_). The resultant pressure mapping (Figure 5c_8_), read wirelessly by a vector network analyzer, could reflect different postures illustrated in Figure 5c_6_,c_7_. 3D printing can customize mechanical properties by instantly tuning geometry designs. The structure of the sensitive capacitors usually consists of thin conductive layers and a dielectric layer with sophisticated microstructures, which is suitable for 3D printing techniques like DIW, FDM, and InkJet printing. Also, the wireless capability, precision, and adaptability make capacitive sensors an essential tool for advancing medical diagnostics and treatment monitoring, contributing significantly to personalized medicine.

#### 4.1.3 Microfluidic Detectors and Separators

Microfluidic devices have revolutionized the field of disease detection and isolation by enabling precise manipulation of small fluid volumes, which is essential for analyzing biological samples with high sensitivity and specificity. These devices can be used to isolate circulating tumor cells (CTCs) from blood samples, allowing for early detection of cancer metastasis.^[^ ^503^, ^504^ ^]^ Additionally, microfluidics facilitates the detection of biomarkers associated with various diseases, such as proteins, nucleic acids, and small molecules, through integrated lab‐on‐a‐chip systems that perform complex assays with minimal reagent consumption and faster turnaround times. The high surface area and controlled microenvironments within microfluidic channels enhance the efficiency of capturing and analyzing disease‐related particles, leading to more accurate diagnostics and personalized treatment options.^[^ ^505^, ^506^ ^]^

In recent years, 3D printing has gained substantial attention in manufacturing microfluidics due to its fast production, accurate design, cost‐effectiveness, and the ability to produce geometrically complex parts.^[^ ^507^ ^]^ Despite contact with bioreagents, most microfluidics work in vitro, which does not pose extensive requirements on the biodegradability of the printed polymers. Instead, bioinertness becomes a more critical requirement, as no molecules should be released or absorbed, which could impact the analysis results.

**Figure 6:**
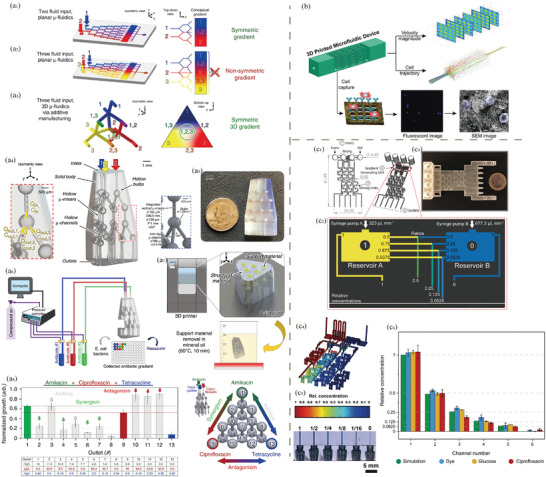
a_1_) Schematic of a conventional planar microfluidic concentration gradient generator (µ‐CGG). a_2_) The limitation of 2D µ‐CGGs, where inputs 1 and 3 form no combinations. a_3_) The advantage of a 3D microchannel network, which can generate symmetric 3D gradients of three input fluids. a_4_) Reverse solids model of the 3D microfluidic channels. a_5_) Photograph of the printed 3D µ‐CGG. a_6_) Schematic of the experiment setups for conducting the antimicrobial susceptibility testing (AST) screening of three antibiotics with the 3D µ‐CGG. Bacterial proliferation would be carried out in the fluids collected from the outlets with various antibiotic gradients. a_7_) The manufacturing process of Multijet 3D printing. a_8_) Three‐antibiotic interaction study, showing (left) E. coli proliferation profiles with fluid collected from 13 outputs and (right) illustration of the 3D channels and the number of outlets. Reproduced with permission.^[^ ^508^ ^]^ Copyright 2020, Springer Nature. b) Schematic illustration of the 3D‐printed microfluidic device (via inkjet 3D printing) for the isolation of circulating tumor cells (CTCs), including the simulated velocity magnitude and particle tracing, and the surface function of the printed parts. Reproduced with permission.^[^ ^509^ ^]^ Copyright 2020, Elsevier. c_1_) The technical drawing of the microfluidic platform for Multijet 3D printing. c_2_) Schematic of the gradient generation unit, with the black numbers indicating the channel length ratios and white numbers indicating the relative concentrations of the antibiotics. c_3_) Photograph of the printed gradient generator (GG). c_4_) The computational fluid dynamic (CFD) simulation of the antibiotic concentration using COMSOL. The total flow rate was 1000 µL min^−1^. c_5_) Photograph showing the GG outlets of a two‐fold dilution series of dye (reservoir A) and water (reservoir B). c_6_) CFD simulation and experimental results of the relative concentrations at the six outlets. The source solutions include a blue dye, glucose, and ciprofloxacin. Error bars represent the standard deviation from three consecutive experiments. Reproduced with permission.^[^ ^510^ ^]^ Copyright 2022, Royal Society of Chemistry.

Sweet et al. aimed to enhance the efficiency of antimicrobial susceptibility testing (AST) for the treatment of antimicrobial‐resistant (AMR) infections. They upgraded the traditional two‐dimensional microfluidic‐based AST platforms to three‐dimensional, which allowed three antimicrobial compounds to be tested simultaneously (**Figure** **6**a_1_–a_3_).^[^ ^508^ ^]^ The 3D microfluidic concentration gradient generator (µ‐CGG) was manufactured by Multijet 3D printing, with a triethylene glycol dimethacrylate‐based polymer as the structural material and a hydroxylated wax as the sacrificial support (Figure 6a_4_,a_5_,a_7_). With three inlets and symmetric branching microchannel networks, the cocktail solutions containing different concentrations of three antibiotics could be collected at the outlets and the corresponding biological incubation could be performed (Figure 6a_6_). For example, Figure 6a_8_ demonstrated a three‐antibiotic interaction study. By comparing the proliferation rate of *E. coli* with the fluid from 13 outputs, the authors found synergism between amikacin and ciprofloxacin, antagonism between ciprofloxacin and tetracycline, and an additive effect between amikacin and tetracycline with one single operation.

Expanding on the previous examples of utilizing 3D printing in enhancing microfluidic devices for biomedical applications, another significant study further demonstrates the versatility and potential of this technology. As one demonstration, Chen et al. leveraged the freedom of 3D printing design and strived to improve the capture efficiency of tumor cells of a microfluidic device fabricated via inkjet printing (Figure 6b).^[^ ^509^ ^]^ The porous structure with a high surface area was produced by multijet printing, followed by surface modification to coat polydopamine and anti‐EpCAM antibodies to immobilize circulating tumor cells (CTCs) at the molecular level. Five internal structures were designed to compare their capture efficiencies in both simulation and experimental results. The optimized design had capture efficiencies up to 92.42% for MCF‐7 breast cancer cells, 87.74% for SW480 colon cancer cells, and 89.35% for PC3 prostate cancer cells. This 3D‐printed microfluidic device could contribute to the isolation of rare tumor cells and detection of cancer metastasis in the future. These innovative approaches highlight how 3D printing not only aids in creating complex geometries but also integrates advanced functionalities to improve diagnostic and therapeutic outcomes. In particular, the ability to customize and optimize designs for specific applications underscores the transformative impact of 3D printing in the field of medical research and treatment.

The ability to integrate multiple functionalities within a single platform demonstrates the customizability and potential of 3D‐printed microfluidic systems. Besides creating sophisticated structures, integration of multiple functions is another merit of 3D‐printed microfluidic platforms, owing to their customizability. Heuer et al. also devoted their efforts to AST diagnostics by assembling photonic silicon chips to the 3D‐printed gradient generators (GG) to rapidly determine the minimum inhibitory concentration (MIC).^[^ ^510^ ^]^ The geometry of the GG was illustrated in Figure 6c_1_‐c_3_, where antimicrobials were injected into reservoir A and a pathogen (e.g., *Escherichia coli*) was introduced into reservoir B. According to the principle of fluid mechanics, a series of antibiotic concentrations would be generated in different channels. The generated gradients of antibiotic concentration were verified through both simulation (Figure 6c_4_) and dilution experiments involving dye, glucose, and ciprofloxacin (Figure 6c_5_‐c_6_). At the end of each channel, there was a growth chamber that allowed the bacteria to proliferate and a photonic silicon chip that analyzed changes in the reflectance spectra of the bacterial growth. This real‐time monitoring shortened the time for determining the MIC to 90 minutes, potentially avoiding the misuse of antimicrobials in clinical practices. The main factor that brought the analysis to practical applications was the versatile platform that was manufactured by MultiJet 3D printing with polyacrylate as the structural material and wax as the sacrificial phase. All three examples chose acrylate‐based polymers as the feedstock due to their UV‐curability, which is compatible with InkJet printing, providing higher resolutions than extrusion‐based methods. Generally, compared to conventional manufacturing protocols (e.g., lithography,^[^ ^511^ ^]^ micro hot embossing,^[^ ^512^ ^]^ and micro injection^[^ ^513^ ^]^), 3D printing provides more possibilities for the design of the microfluidics with exquisite spatial structure, which not only lowers the cost and manufacturing time for the devices, but also improves the efficiency of their sensing, differentiating, and screening functions.

#### 4.1.4 Wearable Microfluidic Sensors

The aforementioned microfluidic devices operate without physical contact with human bodies, necessitating printed polymers that are chemically inert and compatible with biofluids. One significant application of such technology is the development of wearable sweat sensors, which are non‐invasive tools for diagnosing or monitoring human health by quantifying biomarkers.^[^ ^514^ ^]^ These sensors must possess low toxicity to the skin, making biocompatibility crucial. Additionally, the ductility of the printed materials is essential to ensure comfort and functionality when worn. From a geometric perspective, these sensors require thin channels and reservoirs to effectively drain and collect sweat, imposing stringent requirements on manufacturing accuracy. To achieve this precision, advanced 3D printing techniques are employed. For instance, DLP and DIW are often used to fabricate the intricate microstructures necessary for efficient sweat collection and analysis.^[^ ^515^, ^516^ ^]^ The materials used in these methods must be carefully selected for their mechanical properties and ability to form fine, consistent channels. This ensures that the sensors can maintain structural integrity while being flexible enough to conform to the contours of the skin. Moreover, the integration of additional functionalities into these sensors, such as real‐time data transmission^[^ ^517^ ^]^ and multi‐analyte detection,^[^ ^518^ ^]^ further enhances their utility in personalized healthcare.

**Figure 7:**
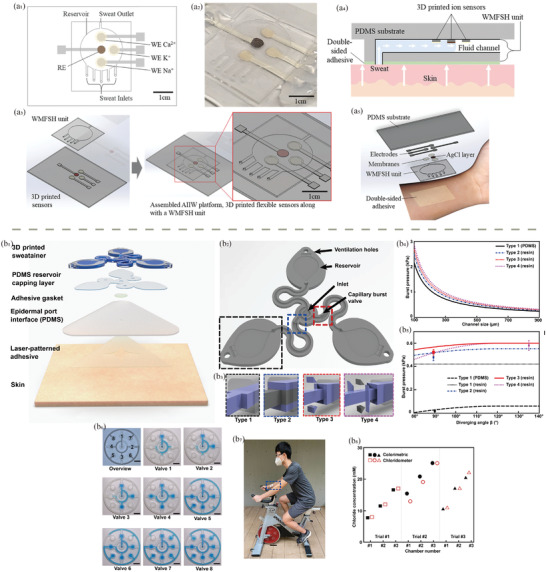
a_1_) Top‐view schematic of the all‐inclusive integrated wearable (AIIW) patch from the DIW 3D printing. a_2_) Photograph of the AIIW patch, consisting of 3D printed ion sensors and a wearable‐microfluidic sample handling (WMFSH) unit. a_3_) Schematic of the components of the AIIW patch before and after assembly. a_4_) Cross‐section of the AIIW patch attached to human skin. a_5_) Individual components of the AIIW patch, including 3D printed electrodes, PDMS substrate, electrodes membrane, adhesive layer, and WMFSH unit. Reproduced with permission.^[^ ^518^ ^]^ Copyright 2021, Wiley. b_1_) Schematic illustration of the key components of the sweatainer system and epidermal interface via the DLP 3D printing technique. b_2_) Structural detail of the sweatainer, including the inlet, capillary burst valves (CBVs), collection reservoirs, and ventilation holes. b_3_) Schematic renders of four designs of CBVs, with the blue areas highlighting the differences between the CBV designs. b_4_) Calculated bursting pressures (BPs) as a function of channel size for a square geometry using the Young‐Laplace equation. b_5_) Theoretical BP as a function of diverging angle β for a channel with a width of 600 µm and a height of 400 µm. b_6_) Sequence of photographs illustrating the liquid entering the chambers of different CBV designs in order. b_7_) Photograph of sweatainer position on the exerciser's skin. b_8_) Measured chloride concentration from both the collection (chloridometer) and colorimetric sweatainers for three independent exercises (stationary cycling for 50 min). Reproduced with permission.^[^ ^520^ ^]^ Copyright 2023, AAAS.

For example, Kim et al. developed a bioelectronic patch to measure multiple electrolyte levels in sweat (**Figure** **7**a_1_‐a_3_).^[^ ^518^ ^]^ The silver‐based conductive ink was DIW printed as the electrodes for selective ion detection (i.e., Na^+^, K^+^, Ca^2+^), and the microchannels in the device were obtained by printing a patterned mold with the same method and then casting PDMS (Figure 7a_4_,a_5_). This case demonstrated the fundamental elements for many wearable sweat sensors, including a thin inlet that allows the fluid to enter the device driven by the combination of the natural pressure of sweat and the capillarity,^[^ ^519^ ^]^ a reservoir with sensors, and a packaging with proper mechanical properties. The DIW printing process enabled precise control over the electrode geometry, ensuring high sensitivity and specificity in ion detection. The patterned mold for the microchannels was crucial for guiding sweat flow into the device, where it could be analyzed in real time. The use of PDMS provided flexibility and durability, making the sensor comfortable to wear while maintaining its structural integrity under various conditions. By integrating these components, the wearable sweat sensor could effectively monitor multiple electrolytes in sweat, providing valuable data for health and fitness assessments. The combination of advanced materials and precise 3D printing techniques exemplifies the potential of wearable technology in non‐invasive health monitoring, offering insights into hydration levels, electrolyte balance, and overall physiological status.

While Kim et al. utilized 3D printing indirectly to create the main structure of their wearable sensor, Wu et al. employed a more direct approach, using DLP to fabricate sophisticated components such as inlets, valves, and reservoirs (Figure 7b_1_).^[^ ^520^ ^]^ This method resulted in a device with a centrally symmetrical design, enabling sweat to flow from a central inlet into three sequential reservoirs (Figure 7b_2_,b_6_). As the vital components for implementing the sequential sweat collection, the capillary burst valves (CBVs) were carefully designed based on fluid surface tension, critical contact angles for the microchannels, channel diverging angles, and diverging channel widths and heights (Figure 7b_3_). Positioned between the main entrance and the reservoirs, the CBVs only allowed the sweat flow above their bursting pressure (BP) threshold (Figure 7b_4_,b_5_), which accumulated the sweat in different reservoirs from different periods of time for further analysis (Figure 7b_6_). To conduct the colorimetric assay on the collected samples, the printing parameters were optimized to obtain high optical transparency of the acrylate‐based resin (MiiCraft BV‐007A), while maintaining acceptable printing resolutions. Resultantly, the time‐dependent chloride concentrations of the sweat were measured from a bicycler as a successful demonstration (Figure 7b_7_,b_8_).

Similarly, Rogers’ team also included the CBVs in their sweat sensor,^[^ ^521^ ^]^ but the authors considered mechanical behavior, as deformations might occur during operation. Choosing methacrylate‐methacrylate (MA‐MA) as the DLP resin, the volume change of the printed fluid microcuvettes was less than 0.5%, and the interfacial stress on the skin was only 14 kPa with a stretch of 30% for a thin device. Besides the chloride concentration, this study extended the quantitative analysis to copper, pH, and glucose in sweat by using colorimetric measurements. These examples underscore the pivotal role of 3D printing in advancing wearable sweat sensors, enabling the fabrication of intricate structures and thin designs that traditional manufacturing methods find challenging. Compared to other microfluidic devices, wearable sensors require polymers with exceptional mechanical compliance and robustness due to the dynamic nature of skin curvature and strain. This necessitates careful selection of materials that can maintain flexibility and integrity under continuous deformation.

However, the integration of 3D printing in microfluidics also presents several challenges. One significant hurdle is achieving high‐resolution prints necessary for creating detailed microchannels and small features essential for effective fluid control and sensing. Current 3D printing technologies like DLP and DIW offer improved resolutions, but further advancements are needed to match the precision required for complex biomedical applications.^[^ ^522^ ^]^ Another challenge is the material properties. Many biocompatible and flexible materials suitable for wearable applications do not easily lend themselves to high‐resolution 3D printing. Developing new printable materials that combine biocompatibility, flexibility, and mechanical strength remains a critical area of research. Additionally, ensuring the reliability and reproducibility of printed microfluidic devices can be difficult,^[^ ^523^ ^]^ as variations in print quality can affect the performance of the sensors. Furthermore, integrating multiple functionalities within a single device, such as combining fluid handling with electronic sensing capabilities, requires sophisticated design and fabrication processes. This often involves multi‐material printing and precise control over the spatial distribution of different materials,^[^ ^524^ ^]^ which adds to the complexity of the manufacturing process.

#### 4.1.5 Microneedles as Sensors

**Figure 8:**
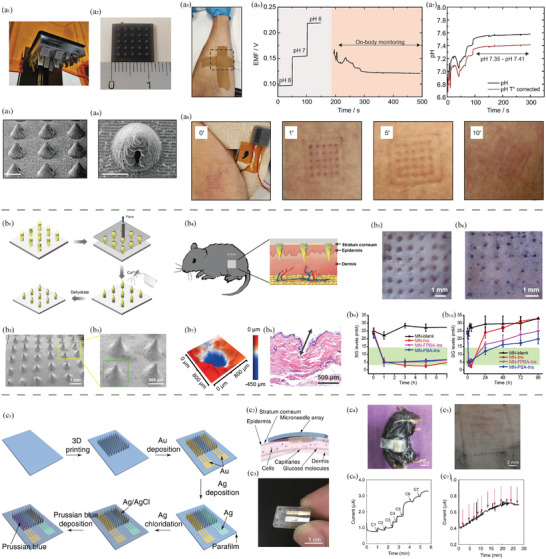
a_1_) SLA platform that manufactured the microneedles. a_2_) Photograph of the 3D‐printed hollow microneedle (HMN) array. SEM images of a_3_) the HMN array (scale bar: 1 mm) and a_4_) a single HMN (scale bar: 500 µm). a_5_) Photograph of the microneedle (MN) pH sensor attached to the forearm. a_6_) Potentiometric response of the MN pH sensor from both the in vitro calibration (left) and the on‐body experiment (right). a_7_) Temperature correction of the recorded interstitial fluid pH. a_8_) Effect of the piercing of the MN sensor on the skin after the test. Reproduced with permission.^[^ ^526^ ^]^ Copyright 2023, Elsevier. b_1_) Illustration of the fabrication process of the MN patches, including 3D printing, stretching, spraying of Ca^2+^ ions, and dehydration. b_2_,b_3_) SEM images of the MN patch. b_4_) Schematic of the MN patch working on the dorsal skin of a mouse. b_5_) Photograph of the mouse skin pierced by the MN for 10 min. b_6_) Trypan blue staining, b_7_) 3D optical profiler image, and b_8_) H&E staining image of the pierced mouse skin. In vivo blood glucose monitoring with the MN sensor of the mice within b_9_) 5 h and b_10_) 4 days. Reproduced with permission.^[^ ^529^ ^]^ Copyright 2020, Elsevier. c_1_) Fabrication process of the MN biosensor. c_2_) Schematic of the MN array inserted into the dermis. c_3_) Photograph of the 3D‐printed cone‐shaped MNs with a base diameter of 400 µm and a height of 1.5 mm. c_4_) Photograph of the MN array applied to a mouse. c_5_) Magnified image of the mouse skin pieced by the MN array biosensor. c_6_) In vitro sensing of glucose in different PBS solutions. (C1: 0.8 mM, C2: 2.2 mM, C3: 3.0 mM, C4: 6.0 mM, C5: 12 mM, C7: 14 mM) (c_7_) In vivo sensing of subcutaneous glucose in a mouse injected with glucose. Reproduced with permission.^[^ ^531^ ^]^ Copyright 2021, Springer Nature.

Microneedles (MNs) are arrays of micro‐scale needles used for sensing and drug delivery purposes, offering advantages such as painlessness and minimal invasiveness due to their small size.^[^ ^525^ ^]^ These attributes make them particularly appealing for various medical applications. The intricate and subtle structures of MNs are ideally suited for 3D printing, which allows for precise fabrication of these complex designs with high resolution and accuracy crucial for their performance and functionality. However, the invasive nature of MNs necessitates higher biocompatibility standards for the chosen biopolymers compared to non‐invasive sensors like those used for sweat monitoring. Polymers such as PLA, PCL, and GelMA are often used due to their favorable properties. In contrast, Parrilla et al. employed SLA to manufacture MN patches with hollow structures (**Figure** **8**a_1_‐a_4_).^[^ ^526^ ^]^ The micropores were filled with conductive ink (i.e., graphite paste), and a thin layer of PANI was electrodeposited onto the surface of the MN. The PANI monitored protons through the transition between emeraldine salt and emeraldine base under different pH conditions, thus revealing the pH value of the skin interstitial fluid by evaluating the open circuit potentials between the reference electrode and the MN. A demonstration showcased the accurate monitoring of the pH value after proper calibration (Figure 8a_5_‐a_7_), with minimal damage to the skin (Figure 8a_8_). Although conjugated polymers, including PANI, polypyrrole (PPy), poly(3,4‐ethylenedioxythiophene) (PEDOT), and poly(3‐hexylthiophene) (P3HT), lack biodegradability, they are used in neuronal tissue engineering,^[^ ^527^ ^]^ as the regeneration of neurons can benefit from electrical stimulation.^[^ ^528^ ^]^ From this perspective, PANI's biocompatibility is considered acceptable for Parrilla's case, as the MN patch will be removed afterward.

3D printing enables the integration of multiple materials into a single device, which can enhance the functionality of the MNs, such as incorporating drug reservoirs or sensors within the needle structure. Wu et al. used another 3D printing strategy, DIW, to produce the MNs in their preliminary state.^[^ ^529^ ^]^ The post‐treatment modified their morphology and toughened the material, leveraging the gelation behavior of sodium alginate (Figure 8b_1_‐b_3_). Since the printing feedstock contained gluconic acid‐modified bovine insulin, the insulin would be released from the MN upon encountering high glucose levels through a chemical reaction, forming a rapid sense‐release cycle. The penetration tests on mice showed limited harm to the skin (Figure 8b_4_‐b_8_), and in vivo studies demonstrated successful short‐term (Figure 8b_9_) and relatively long‐term (Figure 8b_10_) control of glucose levels in the mice. In this study, the printing ink shows the potential for high biocompatibility, as alginate is a typical natural polymer, and hydroxyapatite, used as a rheology modifier, is commonly applied in bone tissue engineering.^[^ ^530^ ^]^ Meanwhile, the versatility of DIW allows simultaneous extrusion of the polymer solution, mineral particles, and small‐molecule medicine without damaging their structures. Also focusing on diabetes and glucose level, Liu et al. manufactured the MN sensor through a series of DLP printing, magnetron sputtering, and electroplating processes (Figure 8c_1_).^[^ ^531^ ^]^ In the presence of subcutaneous glucose, H_2_O_2_ was produced on the working electrode through an enzymic reaction, thereby generating a current signal (Figure 8c_2_‐c_3_). After in vitro calibration, the MN sensors successfully detected changes in the glucose levels of mice (Figure 8c_4_‐c_7_), and the continuous monitoring can last as long as one week. Although the authors did not specify the chemical formulation of the UV‐curable resin, the animal tests partially demonstrated the sensor's adequate biocompatibility. These cases indicate that multiple 3D printing strategies are capable of manufacturing MN sensors. The biodegradation of MNs is usually not a concern as removed after serving their function. However, the coatings and deposited chemicals that penetrated into the skin should be considered.

Many introduced 3D‐printed biosensors operate in vitro or do not penetrate the skin deeply. Thus, biodegradation is often neglected. The challenge might lie in achieving both functionality and biodegradability in the same material. Moreover, the performance of the functional materials usually deteriorates, making it hard to calibrate the signals. A strategy may be to distinguish between functional materials and necessary support materials. Kong et al. developed a wireless gastric resident electronic device that relied on an FDM‐printed geometry to enable the sensor to reside in a hostile gastric environment for more than a month.^[^ ^532^ ^]^ The degradation of PLA, the support material, then caused the structure to disintegrate, allowing the safe passage of the device from the gastric space after serving its purpose. Although the biological environment differs between the digestive system and other tissues, this project serves as a significant inspiration for 3D‐printed degradable sensors. Bao's group created a completely degradable in vivo arterial‐pulse sensor capable of real‐time wireless blood flow monitoring.^[^ ^499^ ^]^ It is a brilliant demonstration of biodegradable biosensors; however, 3D printing was not included in this system. Given recent advances in degradable biosensors and 3D‐printed devices, the merging of these two ideas might be imminent. A more detailed introduction to 3D‐printed biomedical sensors has been published by Ali et al.^[^ ^533^ ^]^

### 4.2 Actuators for Imitating Biological Movements

Actuators that possess shape adaptability to ambient environments are garnering research attention for their potential to serve as robotic locomotors and manipulation grippers.^[^ ^534^ ^]^ These adaptable actuators can emulate certain biological actions and are the basis for soft robotics. With the appropriate size and degrees of freedom, bioactuators find applications in physiological monitoring,^[^ ^535^ ^]^ artificial muscles,^[^ ^536^ ^]^ and drug delivery carriers.^[^ ^537^ ^]^ The ability of bioactuators to adapt their shape in response to environmental stimuli is particularly valuable in health applications.^[^ ^534^ ^]^ For instance, bioactuators used in artificial muscles can mimic natural muscle contractions, providing more effective and comfortable prosthetic limbs.^[^ ^538^ ^]^ In physiological monitoring, bioactuators can adapt to the body's movements, ensuring continuous and accurate data collection without causing discomfort to the patient. In drug delivery, shape‐adaptive actuators can navigate through the body's complex environments to deliver medication precisely where needed, enhancing treatment efficacy and reducing side effects.^[^ ^539^ ^]^ Additionally, bioactuators can be utilized in minimally invasive surgical tools, offering precise manipulation and control within the body, reducing patient recovery time and surgical risks. Also, bioactuators can assist in rehabilitation devices,^[^ ^540^ ^]^ offering controlled and programmable movements to aid patients in regaining strength and mobility, customizing therapy to individual needs and improving overall outcomes.

**Figure 9:**
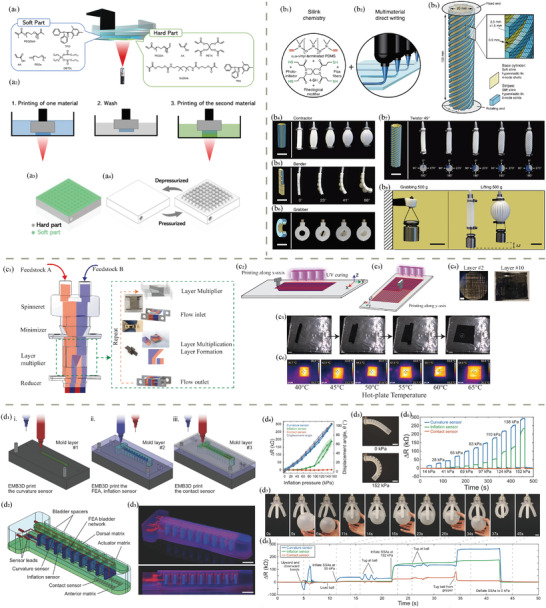
a_1_) Resin composition for the soft and rigid materials for SLA. a_2_) Process flow for the multi‐material SLA printing. a_3_) Design of a 3D‐printed microactuator chip with an air inlet on the side. The size of the chip was 20 mm × 20 mm × 3 mm, with a soft membrane of 200 µm thickness. a_4_) The unactuated state of the chip has an flat surface, while the inflated state of the chip features the soft membrane protruding and expanding. Reproduced with permission.^[^ ^541^ ^]^ Copyright 2023, MDPI. b_1_) Chemical schematic of vinyl‐terminated silicones, blended with a thiol‐crosslinker, fumed silica as rheology modifier, flax fibers as reinforce filler, and a photoinitiator. b_2_) The multimaterial direct ink writing seamlessly used to produce the actuators in a single print. b_3_) Geometry and parameters of the actuator in twisting mode. b_4_) Actuator in contraction mode, where stiff strips were along the long axis of a soft silicone tube (lead angle α = 0°). b_5_) A bender was obtained by printing soft silicone chambers onto a stiff silicone film. Inflating the air cavities with a pressure of 6 kPa resulted in bending angles up to 90°. b_6_) A grabbing actuator consisted of a larger stiff silicone cylinder and a concentric inner soft tube that could be inflated. b_7_) A twisting motion was achieved by winding slanted stiff strips around the soft inner tube, with α = 45° as an example. The twisting angle increased with the applied internal air pressure. b_8_) Demonstration of the grabbing and contractile actuators bearing loads. Scale bars are 2 cm for b_4_‐b_7_ and 4 cm for b_8_. Reproduced with permission.^[^ ^545^ ^]^ Copyright 2018, Springer. c_1_) Multiphase direct ink writing (MDIW) printing nozzle design, including the spinneret, minimizer, layer multiplier, and reducer. UV‐assisted printing along the in‐plane c_2_) x‐axis and c_3_) y‐axis. c_4_) An optical and digital photograph of thin‐ply stacking films with 2 and 10 printed layers. Scale bar is 500 µm. c_5_) 90°‐rolled 5‐layered composites expanded as a function of time on a hot plate of 60 °C. Scale bar is 1 cm. c_6_) Thermal images showing temperature distributions during the expansion of the composites.^[^ ^37^ ^]^ Copyright 2022, Elsevier. d_1_) Fabrication of the soft somatosensitive actuator (SSA), including the curvature sensor printed within the dorsal matrix (Layer 1); the actuator features and the inflation sensor printed within the actuator matrix (Layer 2); and the contact sensor printed in the anterior matrix (Layer 3). d_2_) Schematic demonstration and d_3_) images under black light exposure of the manufactured SSA, with the fugitive and sensor inks dyed blue and red, respectively, to facilitate visualization. Scale bars are 1 cm. d_4_) Resistance change of the curvature, inflation, and contact sensors and displacement angle as a function of inflation pressure without blockage. d_5_) Photographs of an SSA at 0 kPa (top) and 152 kPa (bottom) during a dynamic free displacement test. d_6_) The corresponding ΔR for each sensor as a function of time, where the 0 kPa and inflation pressures were held for 20 s, respectively, with the increasing inflation pressure in increments of 14–152kPa. d_7_) Images of the gripper grabbing a ball and then being pulled away. d_8_) The corresponding ΔR of each sensor during the interaction shown in (d_7_). Reproduced with permission.^[^ ^546^ ^]^ Copyright 2018, Wiley.

3D printing offers a multitude of design freedoms, allowing for the simple tuning of bioactuator properties. This technology enables the precise fabrication of complex geometries and the integration of multiple materials in a single device, which is crucial for creating actuators with tailored mechanical properties and functionalities. As an effective solution, the use of 3D printing allows for the creation of multi‐material actuators that combine rigid and flexible elements to achieve desired actuation behaviors. For example, Song et al. fabricated a multi‐material actuator system using SLA (**Figure** **9**a).^[^ ^541^ ^]^ For the actuation functions, the flexible phase was based on poly(ethylene glycol) dimethacrylate (PEGDMA) and PAA, while the rigid phase consisted of tetraacrylated and diacrylated crosslinkers (Figure 9a_1_). The material switch was conducted by changing the vat during the printing process (Figure 9a_2_). The authors thus developed a microactuator with the rigid part serving as the air chamber and the flexible part as and the inflatable membrane (Figure 9a_3_). The membrane deforms when pressurized, protruding from the hollow part of the chamber to alter the surface roughness of the chip (Figure 9a_4_). Additionally, 3D printing can produce actuators with embedded sensors and electronics, enhancing their functionality and integration into medical devices. As one demonstration, Odent et al. demonstrated a family of multi‐responsive actuators manufactured by SLA.^[^ ^542^ ^]^ The hydrogel can react to temperature and pH due to the low critical solution temperature of poly(N‐isopropylacrylamide) (PNIPAM) and the acidic dissolution of poly(2‐carboxyethylacrylate) (PCEA) in the composite, respectively. Three strategies of chemomechanical gradients, including surface area to volume ratio, crosslinking density, and chemical composition, were successfully tried to achieve controllable motion. This work typified the benefits of additive manufacturing in the design and manufacture of bioactuators, where its flexibility and customizability endowed the same polymers with the ability to respond to different stimuli, enriching the potential for varied applications. Although lacking biocompatibility tests, some literature indicated that PNIPAM and PCEA materials have acceptable cytotoxicity^[^ ^543^, ^544^ ^]^ and proper actuator integration into bio‐related environments.

3D printing in advanced actuator manufacturing not only facilitates the rapid prototyping and customization of bioactuators but also enables the production of actuators with intricate internal structures that would be challenging to achieve with traditional manufacturing methods. One versatile actuator design was well demonstrated by Schaffner et al. (Figure 9b_1_–b_3_), particularly in the systematic discussion of the twisting mode (Figure 9b_3_).^[^ ^545^ ^]^ The authors carried out multimaterial direct writing of α,ω‐vinyl‐terminated PDMS (Figure 9b_1_,b_2_). The added flax fibers, serving as a reinforcing phase, increased the stiffness of the rigid phase. Upon inflation, the stiffness of the actuator was dominated by the stiff strips, while its transverse behavior relied on the high compliance of the soft base layer (Figure 9b_3_). Governed by the lamination theory, the actuator could take on contraction, elongation, and twisting motion modes, by altering the lead angle of the design. Notably, the inclusion of a thiol‐crosslinker improved the interface strength not only between the printing layers, but also between the silicone inks with different stiffness, ensuring the robustness of the structures. Various functions of different actuator designs were demonstrated in Figure 9b_4_‐b_8_, showcasing the feasibility and versatility of this printed system. In addition to the bioinert PDMS, flax fibers are derived directly from nature, ensuring the total biocompatibility of the material system. Besides, incorporating multiple motion modes closely mimic the movement of real creatures for prosthetics.

The versatility and precision of 3D printing make it an indispensable tool in the development of advanced bioactuators for a wide range of health applications (e.g., drug delivery or smart tissue scaffolds), potentially leading to more effective and innovative solutions in medical technology. For instance, Ravichandran et al. developed a 3D printing technique called multiphase direct ink writing (MDIW) to produce a dual stimuli‐responsive actuator (Figure 9c).^[^ ^37^ ^]^ The one‐step formation of the multiphase was achieved by a unique printhead, where the consecutively connected multipliers could divide two feedstocks and generate 2^n+1^ alternating layers in a single move (*n* represents the number of the multipliers) (Figure 9c_1_).^[^ ^547^ ^]^ The printed TPU‐D (Ellastollan 1254 D 13 U)/PCL region was capable of shape fixing and recovery due to the temporary shape retention effect contributed by the stiff crystal region in PCL and the recovery effect caused by the molecular relaxation at higher temperature of the amorphous phase in TPU‐D. Consequently, thermal actuation could be activated by heating the layered composite that had been frozen into certain shapes (Figure 9c_5_,c_6_). This work provides inspiration for customizing 3D printing platforms to manufacture bioactuators effectively, and the material choice of PCL and TPU poses no issues with biocompatibility.

While bioactuators perform functions such as bending, lifting, and grasping, integrating sensors into actuating systems creates more possibilities in the field of smart wearables and haptic devices. Lewis's group reported a soft somatosensitive actuator that possessed the abilities of haptic, proprioceptive, and thermoceptive sensing when performing its grasping function.^[^ ^546^ ^]^ 1‐ethyl‐3‐methylimidazolium ethyl sulfate (EMIM‐ES) and Pluronic F127 were deposited as the conductive ionogel for sensors and the fugitive ink for pneumatic channels, respectively, via multimaterial, embedded 3D (EMB3D) printing (Figure 9d_1_). With different deformations of the sensors that were situated in different matrices (Figure 9d_2_,d_3_), their resistance change reflected and thus interpreted the movement pattern of the actuator. For instance, the curvature sensor responded to the bending angle, the inflation sensor read the active force that drove the actuator, and the contact sensor recorded the contact pressure that was applied at the surface of the anterior matrix (Figure 9d_4_–d_6_). The authors successfully demonstrated the distinct signals of the sensing system during the whole cycle of manipulating the actuator to grab a ball and tugging the ball from it in Figure 9d_7_,d_8_. The trial sheds light on integrated closed‐loop control of bioactuators. The human body is a large and complex functional system with plenty of bioactuators. Moreover, these natural actuators that harvest chemical energy usually exhibit higher efficiencies (≥50%) compared to man‐made ones (<30%).^[^ ^548^ ^]^ This indicates that finer structures and more biocompatible polymers are significant elements for the future of 3D‐printed bioactuators. Meanwhile, Lewis's group has considered how organisms operate, illustrating the potential for 3D‐printed bioactuators to possess the ability to adjust their motion under external stimuli, resembling what we commonly refer to as ‘smart.’ This capability opens new possibilities for creating more adaptive, responsive, and efficient devices that can integrate seamlessly into biological systems, offering advanced solutions in medical and robotic applications. Cases of 3D‐printed soft actuators were provided thoroughly by Zhang et al.^[^ ^549^ ^]^

### 4.3 Wearable Soft Robotics Replicating Muscular Motions

**Figure 10:**
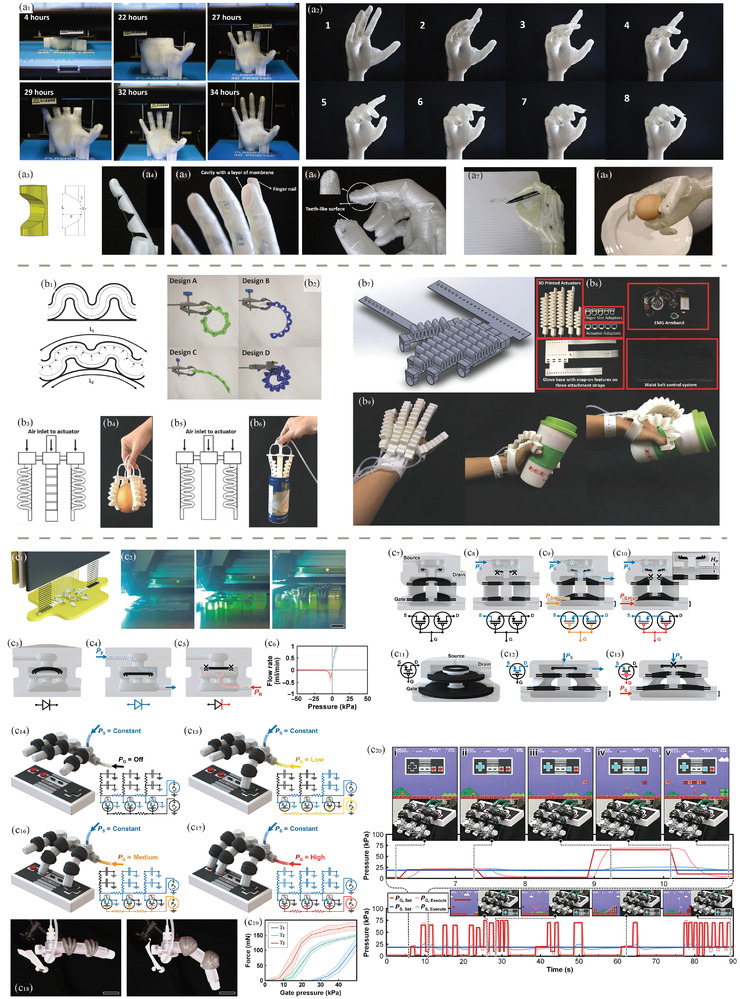
a_1_) The time lapse of FDM printing of the prosthetic hand. a_2_) Finger movement for performing pinch grasp. a_3_) Schematic of the corner‐filleted flexure joint. a_4_) Photograph of a printed finger with three corner‐filleted flexure joints. Details of the printed finger including a_5_) the fingernail and a cavity in the fingertip for higher contact surface area in pinch grasp, and a_6_) the teeth‐like surface on the index fingertip and the thumb for stable grasp. Photographs of the printed hand a_7_) performing a tripod grasp and a_8_) grasping a fragile, spherical object. Reproduced with permission.^[^ ^552^ ^]^ Copyright 2020, PLOS. b_1_) Illustration of the mechanism of the 3D‐printed pneumatic actuator, whose asymmetrical strain was caused by the constant length of the strain limiting layer (L_2_) and the increased length of the inflated serpentine (L_1_). b_2_) Image analysis for calculating the curvature indices of four printed designs, all pressurized at 200 kPa. b_3_) Schematic of the gripper with four printed actuators bending inward picking up b_4_) an orange. b_5_) Schematic of the gripper with outward facing actuators grasping b_6_) inside of a cylindrical can. b_7_) CAD model of the assistive glove. b_8_) Components of a rehabilitative device, including the 3D‐printed actuators, that use electromyography muscle signals for control. b_9_) The 3D‐printed assistive glove facilitating hand grasping. Reproduced with permission.^[^ ^555^ ^]^ Copyright 2018, Wiley. c_1_) Conceptual illustration of the multimaterial PolyJet 3D printing of the fluidic circuitry embedded robots, using rigid (white), compliant (black), and sacrificial (yellow) materials. c_2_) Time‐lapse images of the corresponding 3D printing process. Scale bar, 2 cm. c_3_–c_5_) Operation principles of the 3D‐printed fluidic diode. The architecture of the diode in its c_3_) resting state, c_4_) “forward flow” state, and c_5_) “reverse flow” state. c_6_) Experiment data of the directional flow rate versus pressure for the printed diode. c_7_–c_10_) Operation principles of the 3D‐printed normally closed fluidic transistor. The architecture of the normally closed transistor in its c_7_) resting state, c_8_) “closed” state, c_9_) “open” state, and c_10_) “reclosed” state, where P_s_ is the input source pressure, and P_G_ is the input gate pressure which could be low and high. c_11_–c_13_) Operation principles of the 3D‐printed normally open fluidic transistor. c_11_) The architecture of the normally open transistor in its c_11_) resting state, c_12_) “open” state, and c_13_) “closed” state. c_14_–c_17_) Conceptual illustration of the aperiodic fluidic input‐based soft robotic hand and analogous circuit diagrams of the integrated fluidic circuitry. Four primary states are shown, with a constant P_s_ input and different P_G_ magnitudes. The integrated normally open fluidic transistors possessed distinct pressure‐gains (γ) (γ_1_ < γ_2_ < γ_3_). Photographs of a soft robotic finger with a γ_3_ transistor under P_s_ = 20 kPa, with P_G_ = (c_18 left_) 0 kPa and (c_18 right_) 20 kPa. c_19_) Recorded fingertip actuation force versus P_G_, driven by the fluidic transistor systems with three different γ and constant P_s_ of 10 kPa. c_20_) Demonstration of the robotic hand playing the Super Mario Bros. video game controlled by a preprogrammed P_G_ input (P_s_ remained constant). Insets include the game state and the corresponding controller activation state as well as the photograph of the robotic hand pressing the controller in real time. Reproduced with permission.^[^ ^556^ ^]^ Copyright 2021, AAAS.

The history of wearable robotics dates back to the 1960s, initially developed to support the NASA Apollo program. Over time, wearable robotics have evolved to include systems for first responders, haptic devices, and rehabilitation and assistive robotics.^[^ ^550^ ^]^ These systems must be durable, meaning biodegradability is typically not a concern. Instead, compatibility with human skin and other body parts is crucial. 3D printing has revolutionized the development of wearable soft robotics, offering design flexibility and the ability to create complex structures in a single step.^[^ ^551^ ^]^ 3D printing also allows for the creation of customized, patient‐specific devices that can significantly enhance the quality of life for individuals requiring prosthetics or other assistive devices. The ability to print complex structures with integrated functionality in a single process streamlines the manufacturing process and reduces costs, making advanced wearable robotics more accessible. For example, Mohammadi et al. introduced an FDM‐printed prosthetic hand (**Figure** **10**a_1_,a_2_) capable of performing real‐world tasks such as the pinch grasp of an egg and the tripod grasp of a pencil (Figure 10a).^[^ ^552^ ^]^ The fingers contain specially‐designed monolithic flexure joints (Figure 10a_3_,a_4_), which were manufactured with 3D printing in one step (Figure 10a_1_). The highly compliant TPU and the innovative structure (Figure 10a_5_,a_6_) both contribute to its capability of replicating practical tasks, including a power‐grip force of 21.5 N, and a finger flexion speed of 1.3 s. The prosthetic hand is powered by micromotors, aiming to mimic human limbs as closely as possible (Figure 10a). The choice of TPU ensures adequate biocompatibility and provides suitable friction for grasping. Additionally, elastomers like silicone are widely used for their flexibility, resilience, and ability to withstand repeated stretching and compressing without degrading.^[^ ^553^, ^554^ ^]^ These properties are crucial for components in wearable soft robotics that need to adapt to body movements while maintaining functionality. Other advanced materials, such as conductive polymers and hydrogels, are employed for their unique electrical and mechanical properties, enabling the creation of sophisticated and responsive wearable robotic systems that enhance user comfort and performance.

Also, the versatility of FDM materials and techniques enables the development of devices that are both functional and comfortable for rehabilitation purposes in addition to manual human hands as demonstrated above. For example, Ang and Yeow used pneumatic actuators from FDM as components to develop rehabilitation gloves (Figure 10b).^[^ ^555^ ^]^ The actuators consisted of a serpentine tube that expanded when inflated, and a solid line that confined the strain on one side (Figure 10b_1_). The asymmetrical strain drove the actuator to bend towards the side with the solid line, due to the flexibility of the TPU material as well. Because the actuators were FDM printed, various designs of the serpentine structure were efficiently produced and analyzed, presenting distinct curvature indices (Figure 10b_2_). The assembled robotic gripper was able to grasp different objects from both inward and outward directions, as demonstrated in Figure 10b_3_–b_6_. Based on the gripper, a rehabilitative glove device was invented, whose bending motion mimicked the movements of human fingers, augmenting stroke patients’ finger movement (Figure 10b_7_‐b_9_). The work resonates with the idea of exoskeleton, yet inclusion of sensing systems that enable precise control demands further research.^[^ ^555^ ^]^

The integration of new 3D printing in the development of wearable soft robotics represents a significant advancement in health applications, such as in assistive devices. Sochol's group proposed a fully 3D‐printed soft robotic system that was precisely controlled by its integrated fluidic circuitry (Figure 10c).^[^ ^556^ ^]^ The multimaterial PolyJet 3D printing method allowed simultaneous production of the flexible interconnects, body features, and fluidic circuit channels with a commercialized soft photopolymer (Agilus30), a dental resin (MED610), and a water‐soluble sacrificial support material (SUP706), respectively (Figure 10c_1_,c_2_). Similar to electrical networks, the 3D‐printed elements functioned as diodes and transistors but were manipulated by fluids (Figure 10c_3_‐c_13_). For example, Figure 10c_3_–c_5_ illustrated a fluidic diode that allowed water to flow through from the top inlet, but prevented the flow from the bottom. This is because the free‐floating sealing disc would be pushed and come into contact with the top surface, obstructing the fluid. A normally closed fluidic transistor was demonstrated in Figure 10c_7_–c_10_. The input source could only pass when the gate pressure deformed the bottom diaphragm and lifted the top O‐ring via a central rigid piston. Further increasing the gate pressure would cause a larger deformation of the O‐ring in the middle layer, thus reclosing the pathway. The height of the posts in the middle layer could be adjusted to modify the propensity for the orifice, yielding different pressure‐gains (γ). Along with another normally open fluidic transistor shown in Figure 10c_11_‐c_13_, soft robotic turtles could function under different input formats. However, the more appealing application can be the programmed periodic input‐based soft robotic hand (Figure 10c_14_‐c_20_).

Consisting of normally open fluidic transistors with distinct pressure‐gain properties, the number of bent fingers could be controlled by adjusting the applied gate pressure (Figure 10c_14_‐c_17_). Consequently, an enlightening demonstration of the robotic hand using a gamepad was conducted in Figure 10c_20_. Using a preprogrammed aperiodic fluidic input, the robotic hand completed the first level of a classic side‐scrolling video game, Super Mario Bros., by pushing down correct bottoms corresponding to the fluidic input in real time. The study shows the power and versatility of 3D printing in designing and manufacturing the structures and the control systems all at one, followed by mature applications of soft robotics. The case greatly inspires the 3D‐printed wearable robotics in terms of both operation methods and achievable functions. Different trends of soft robotics can be observed from the aforementioned three examples: realistic printed parts as prosthetics, extensions that work synergistically with the human body, and independent systems that accomplish human tasks. Each branch possesses great potential and promises to significantly improve the quality of life. For example, assisting a disabled person in daily activities. Additionally, their applications also encompass soft tools for surgery, artificial organs, and tissue‐mimicking active simulators for studies, which require varying degrees of biocompatibility and biomimicry.^[^ ^557^ ^]^ While polymeric biomaterials address biocompatibility concerns, 3D printing plays a critical role in achieving biomimicry. The combination of these two factors perfectly meets the requirements for creating biomedical soft robotics. With the synergistic advancement of bidirectional neural interactions, some proof‐of‐concept studies have demonstrated the significantly progress of anthropomorphic prosthetics.^[^ ^558^ ^]^ The situation implies that there may be an increasing demand for 3D printing and engineering of polymeric biomaterials in the near future. Yap et al. has published a comprehensive review with more successful cases on 3D‐printed soft robotics.^[^ ^559^ ^]^

### 4.4 Passive Energy Storage Systems for Health Purposes: Batteries, Supercapacitors, and Fuel Cells

The introduction of power storage, generation, and management in health applications plays a crucial role in enhancing patient care and medical device functionality. Advanced energy storage systems, such as batteries, supercapacitors, and fuel cells, are integral to the operation of both in‐body and out‐of‐body medical devices.^[^ ^560^ ^]^ For instance, implantable devices like pacemakers and neurostimulators rely on compact, reliable batteries to ensure continuous operation.^[^ ^561^ ^]^ Similarly, wearable health monitors and emergency medical equipment benefit from efficient energy storage and management systems to provide real‐time data and rapid response capabilities.^[^ ^562^ ^]^ Also, the need for efficient energy storage systems is paramount in managing diabetes and assisting heart functions in artificial hearts. Continuous glucose monitoring systems (CGMs) and insulin pumps for diabetes management require reliable batteries to operate seamlessly, delivering insulin and monitoring glucose levels without interruption.^[^ ^563^ ^]^ Similarly, artificial hearts or ventricular assist devices (VADs), which support or replace the pumping function of the heart, depend on advanced battery systems to ensure life‐sustaining performance.^[^ ^564^ ^]^ These applications highlight the critical role of energy storage technologies in providing sustainable power solutions for life‐saving medical devices.

As a significant power source for biosensors and smart wearables, batteries have made momentous progress regarding its energy and power density, reliability, and cyclability.^[^ ^565^ ^]^ To achieve higher energy and power densities, 3D printing is gaining attention due to its ability to produce intricate 3D structures that enhance the mass loading of active materials without extending ion transport distances, thanks to high surface‐to‐volume ratios.^[^ ^566^, ^567^, ^568^ ^]^ Therefore, application of 3D printing in batteries has been carefully reviewed.^[^ ^568^, ^569^, ^570^ ^]^ However, few studies have focused on the printing of polymeric biomaterial‐based batteries. For example, Maurel et al. explored the possibility of maximizing the graphite loading of graphite/PLA composite filaments that could be printed as negative electrodes in lithium‐ion batteries.^[^ ^571^ ^]^ They produced FDM printed negative electrode discs reaching 200 mAh/g of active material at a current density of C/20 after 6 cycles, though no attractive geometries were studied. Also, Gao et al. also used PLA as the matrix to create the 3D structured carbon framework with FDM, but the PLA was chemically removed before the structure was covered with PANI and polyacrylic acid (PAA) to serve as the cathodes of Zn‐organic batteries.^[^ ^572^ ^]^

The challenge in designing a fully degradable battery lies in ensuring that all components, including electrodes, electrolytes, and casings, are made from biodegradable materials.^[^ ^573^ ^]^ While using one or two biodegradable materials can reduce environmental impact, the remaining non‐degradable parts still pose a significant issue. Achieving full biodegradability requires innovations in materials science to develop alternatives for conventional battery components. These materials must also maintain the performance characteristics required for practical applications, such as energy density, stability, and longevity. This complexity involves not only creating new materials but also ensuring they work synergistically within the battery system. Researchers must also consider the manufacturing processes, such as 3D printing, to integrate these new materials effectively, adding another layer of complexity.^[^ ^568^ ^]^ Successful development of fully degradable batteries would revolutionize the field, making implantable medical devices safer and more environmentally friendly, but significant scientific and engineering hurdles remain. For example, Huang et al. recently developed a fully biodegradable battery with magnesium as the anode, molybdenum trioxide as the cathode, and alginate hydrogel as the electrolyte.^[^ ^574^ ^]^ Although it is a primary battery system, its biocompatibility and biodegradability were proven through both in vitro and in vivo tests. The result opens new possibilities for implantable devices; however, it has not yet been explored for manufacturing with 3D printing.

Supercapacitors and fuel cells are also grappling with the challenge of full biodegradability. For instance, Chen et al. developed an all‐wood biodegradable supercapacitor using wood carbon as the anode, wood membrane as the separator, and MnO_2_/wood as the cathode.^[^ ^575^ ^]^ Similarly, Winfield et al. devised a biodegradable stack of microbial fuel cells, utilizing PLA for the frames, natural rubber as the cation‐exchange membrane, and egg‐based materials for the cathodes.^[^ ^576^ ^]^ Thus, achieving fully degradable energy storage and generation systems involves developing new biodegradable materials for all parts of the system and ensuring they function effectively together. Although some studies have explored 3D printing biodegradable components for energy systems, achieving a completely degradable system through additive manufacturing remains elusive. The establishment of performance criteria and demand for biodegradable energy systems could drive further advancements in 3D printing technologies for batteries, supercapacitors, and fuel cells with potential benefits for medical applications.

### 4.5 Self‐Powered Devices for Biomedical Systems: Thermoelectric, Piezoelectric, Triboelectric, and Pyroelectric Devices

The advent of self‐powered devices represents a significant leap in the development of biomedical systems.^[^ ^577^ ^]^ These devices harness energy from the body's own biomechanical movements, eliminating the need for external power sources, and thereby enhancing their application potential in wearable and implantable technologies. Thermoelectric devices convert body heat into electrical energy, providing a continuous power supply for low‐energy biomedical sensors.^[^ ^578^ ^]^ Piezoelectric devices generate electricity from mechanical stress, which can be used in monitoring physiological parameters like heart rate and muscle activity.^[^ ^579^ ^]^ Triboelectric devices, through contact electrification and electrostatic induction, can power sensors that track motion and other biomechanical activities.^[^ ^580^ ^]^ Pyroelectric devices leverage temperature changes to produce energy, useful in dynamic environments where heat fluxes are common.^[^ ^581^ ^]^ The integration of these self‐powered technologies into biomedical systems ensures a more seamless and efficient operation, ultimately leading to more advanced and responsive healthcare solutions. For example, they can power glucose monitors for diabetes management, cardiac pacemakers, and sensors for real‐time health monitoring without the need for frequent battery replacements. The role of 3D printing in fabricating these devices is crucial, offering precise control over material properties and device architecture,^[^ ^582^ ^]^ thus optimizing their efficiency and functionality in biomedical applications.

**Figure 11:**
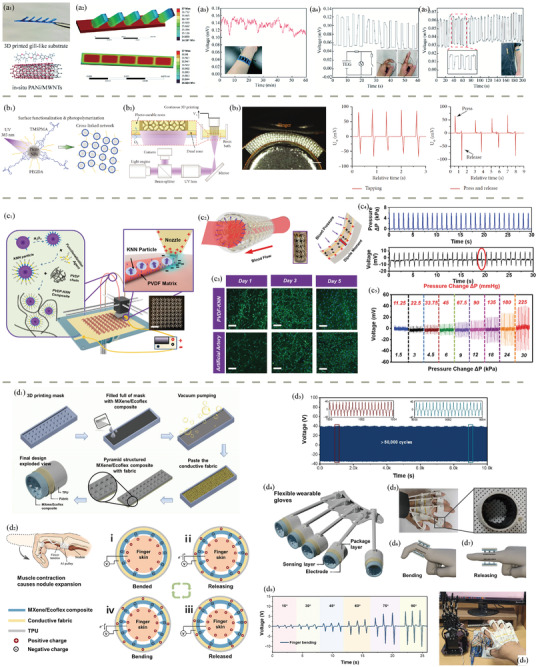
a_1_) Photograph of the FDM printed gill‐mimicking substrate and schematic image of the π‐π interaction between PANI and MWCNTs. a_2_) Simulated temperature distribution of thermoelectric generators with the gill‐mimicking morphology (top) and a flat design (bottom) with the bottom surface in contact with the skin. a_3_) Voltage generated when the thermoelectric generator (TEG) was attached to a human forearm. a_4_) Recorded voltage variation as a function of finger bending, with the inserted circuit diagram of the sensing system and the photo showing finger gestures. a_5_) Voltage change as a response to the chest movement during respiration, with the zoomed‐in figure demonstrating the breathing cycles between 35 and 70 seconds and the photo of the setup. Reproduced with permission.^[^ ^583^ ^]^ Copyright 2021, Royal Society of Chemistry. Schematic of b_1_) the functionalization of the BTO nanoparticles and the photopolymerization during the µCLIP and b_2_) the µCLIP setup. b_3_) Photograph of a 30 wt% functionalized BTO octet‐truss lattice structure, scale bar: 1 mm (left). Recorded voltage signals during the tapping test (middle) and the press‐and‐release test (right). Reproduced with permission.^[^ ^585^ ^]^ Copyright 2022, AAAS. c_1_) Schematic of the electrical field‐assisted FDM platform, with the inserted demonstration of the functionalized KNN nanoparticles and the PVDF matrix (left), and rapid poling of the extruded composite with an inner‐built electric field. c_2_) Schematic illustration of the implanted piezoelectric sensor with the sinusoidal lattice in response to blood pressure. c_3_) Fluorescence microscope images of the 3T3 fibroblasts cultured both on KNN/PVDF film and artificial artery, scale bar: 100 µm. c_4_) Real‐time pressure change in the artificial artery system using a syringe pump to drive the PBS‐simulated blood flow (top), and the corresponding voltage output (bottom). c_5_) Voltage output in response to a series of pressures in the artery system.^[^ ^586^ ^]^ Copyright 2020, Wiley. d_1_) Schematic illustration of the production of the self‐powered toroidal triboelectric sensor (STTS). d_2_) Schematic diagram of the muscle contraction and nodal expansion during finger flexion, and the charge generation and current flow of the STTS in the finger bending cycles. d_3_) Voltage generated by the STTS by 5 Hz external load. d_4_) STTS assembled with a 3D‐printed flexible glove. d_5_) Photograph of a hand wearing the glove with STTS, with the inserted photo of a single sensor. Finger bent d_6_) and released d_7_) wearing the glove, resulting in one output cycle. d_8_) Output voltage of the STTS glove with different bending angles of the finger. d_9_) Photograph of controlling robotic hands with the STTS glove. Reproduced with permission.^[^ ^587^ ^]^ Copyright 2022, Elsevier.

3D printing plays a pivotal role in the development of thermoelectric, piezoelectric, triboelectric, and pyroelectric devices for biomedical applications. By enabling the precise fabrication of intricate geometries and complex structures, 3D printing enhances the performance and integration of these devices. For instance, the technology allows for the creation of micro‐scale features essential for efficient energy conversion in thermoelectric and piezoelectric devices. For example, Zhu etc. proposed a gill‐inspired geometry for wearable thermoelectric generators (TEGs) (**Figure** **11**a).^[^ ^583^ ^]^ The flexible structure was FDM printed with TPU (Figure 11a_1_) to organize the PANI/MWCNTs thermoelectric pellets in an out‐of‐plane way that were beneficial for generating higher voltages on relatively low‐temperature surfaces such as human skin (Figure 11a_3_). By connecting to a strain switch, biosignals, such as joint movement (Figure 11a_4_) and respiratory rate (Figure 11a_5_), could be detected without an external power source. This sensing system unveils the potential for long‐term health monitoring using body temperature as a stable energy source, and more detailed signals (e.g., joint bending angles and pulse rates) can be further studied. In this project, FDM printing plays an important role in the novel geometry design by enabling the utilization of thermoelectricity to harvest body temperature.

Additionally, the customization capabilities of 3D printing ensure that these devices can be tailored to individual anatomical and physiological requirements, optimizing their effectiveness in wearable and implantable biomedical systems.^[^ ^584^ ^]^ This innovative manufacturing process also facilitates the incorporation of diverse materials, thereby improving the functional properties and biocompatibility of the resulting devices. For example, 3D printing‐enabled piezoelectricity is able to transfer physical movement into electrical signals more directly. Chen's group took advantage of micro continuous liquid interface production (µCLIP) to fabricate architectured piezoelectric sensors more than 10 times faster than the traditional SLA method.^[^ ^585^ ^]^ The printed composite consisted of UV‐curable poly(ethylene glycol) diacrylate (PEGDA) as the matrix and barium titanate (BTO) as the piezoelectric functional phase. To prevent the agglomeration of the BTO nanoparticles and enhance the homogeneity of the printed parts, 3‐(trimethoxysilyl) propyl methacrylate (TMSPMA) was adopted to functionalize the particles so that covalent bonds would form between the PEGDA matrix and the BTO fillers (Figure 11b_1_). The lattice‐structured sensor was tested to read biosignals, including finger tapping (Figure 11b_3_), stomping, and coughing, with voltage changes. Apart from macroscopic movements of the body surface, subtler mechanical signals can be recorded via piezoelectricity. Li et al. used the same 3‐(trimethoxysilyl)propylmethacrylate to covalently graft potassium sodium niobate (KNN) piezoceramic particles, obtaining improved dispersibility in the polyvinylidene fluoride (PVDF) polymer matrix.^[^ ^586^ ^]^ Moreover, when the authors conducted FDM to manufacture their piezoelectric sensor, an electric field was applied between the print core and the bottom plate to rapidly align the dipoles of the KNN/PVDF composite (Figure 11c_1_). This yielded a superb piezoelectric performance of d_33_ > 12 pC/N. To implant the sensor in human arteries and monitoring thrombosis, the sinusoidal lattice was designed to match the mechanical properties of the device with the biological tissues (Figure 11c_2_). The sensor successfully recorded pulsating liquid flow (Figure 11c_4_), where the voltage varied linearly with the applied pressure (Figure 11c_5_). With biocompatibility confirmed by the cell culturing tests (Figure 11c_3_), the in vivo application of the sensor is more attainable.

The triboelectric effect is another promising energy conversion mechanism to convert biological motion, such as contact separation,^[^ ^588^ ^]^ sliding,^[^ ^589^ ^]^ and friction^[^ ^590^ ^]^ into electric signals. For instance, Park's group developed a toroidal triboelectric generator to grasp hand movements which could be processed for realistic table games, home appliance switching, and human‐machine interaction to control robotic hands.^[^ ^587^ ^]^ The pyramid‐structured MXene/Ecoflex composite, serving as the negative layer in the triboelectric sensor, was obtained by using a 3D‐printed TPU template (Figure 11d_1_). The pyramidal structure played an important role in the contact separation mode of the sensor, where the contact area between the skin of the finger and the MXene/Ecoflex pyramids varied during muscle expansion (Figure 11d_2_). During the contact separation cycles, the potential between the electrodes and the ground changed due to triboelectrification and electrostatic induction at the contact surface, causing electron flow. Assembled with a 3D‐printed glove, the movement of the fingers and the thumb could be sensed (Figure 11d_4_‐d_7_), offering interesting potentials for futuristic gaming experiences and next‐generation human‐machine interactions (Figure 11d_9_).

The effects of thermoelectricity, piezoelectricity, triboelectricity, and pyroelectricity are examples of converting mechanical energy into electricity. Thus, these systems are theoretically self‐powered to provide signals for biosensing or connecting to the Internet‐of‐Things (IoT). To increase energy efficiency or sensitivity, fine‐scale structures were needed to amplify the stimulus, which is an area of expertise of 3D printing. However, these signals are relatively preliminary due to the limited power these effects can generate. For more complex functions, such as data visualization and Bluetooth signal transfer, batteries are still needed.^[^ ^591^ ^]^ Yet, there could be benefits, such as extending the battery life of smart wearables, as some people criticize the frequent charging requirement of some commercial ones. As an auxiliary method to improve energy efficiency, self‐powered systems might also facilitate the miniaturization of both in vivo and in vitro health‐related devices by reducing the need for battery capacity, thereby improving comfort. In the examples above, it is commendable that the polymer matrices of the aforementioned projects (i.e., TPU, PEGDA, and Ecoflex) are biocompatible even for in vivo applications. It demonstrates the potential for future implantation of the 3D‐printed self‐powered devices. Son et al. have provided more comprehensive opinions on 3D‐printed energy devices.^[^ ^592^ ^]^ It might also be worth waiting for the emergence of self‐powered systems integrated with more functions, given the advancements in materials science, especially polymer science, as the thermoelectric and piezoelectric figures of merit of polymers are still inferior.

### 4.6 Data Science for Bioplotting for Health Applications

In general, the application of data science in health, biomedical, and injury healing has profoundly transformed healthcare by utilizing data‐driven insights to enhance diagnosis, treatment, and patient care.^[^ ^593^ ^]^ Advanced analytics and machine learning algorithms process diverse data types, including medical images, patient records, and genetic information, to uncover patterns, predict outcomes, and customize interventions.^[^ ^594^ ^]^ This approach supports early disease detection, personalized treatment plans, and continuous patient monitoring, leading to more efficient and effective healthcare practices. By integrating data science with medical expertise, researchers are unlocking new dimensions of understanding, accelerating medical advancements, and improving patient outcomes.^[^ ^595^ ^]^ Artificial Intelligence (AI), Machine Learning (ML), and general data science are also revolutionizing bioplotting and biomaterial processing for health applications. By leveraging advanced algorithms, these technologies enhance the precision and efficiency of bioprinting processes, enabling the creation of highly complex and patient‐specific tissue structures.^[^ ^596^ ^]^ AI and ML facilitate the optimization of printing parameters, predict material behaviors, and ensure the reproducibility of bioprinted tissues. Additionally, data science aids in the analysis and interpretation of large datasets, improving the understanding of biomaterial interactions and fostering innovations in personalized medicine. These advancements are crucial for developing sophisticated, functional tissue constructs and accelerating the progress of regenerative medicine and tissue engineering.

**Figure 12:**
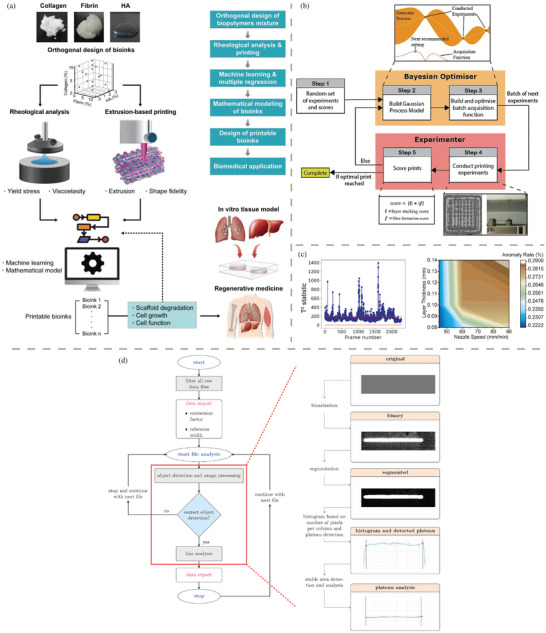
a) Scheme of employing machine learning to predict the relationship between the mechanical properties of the composite ink and the printability, guiding the design of 3D‐printable bioinks comprised of natural polymers. Reproduced with permission.^[^ ^597^ ^]^ Copyright 2020, IOP Publishing. b) Bayesian optimization framework used to evaluate printability and optimize the concentrations of gelatin methacryloyl (GelMA) and hyaluronic acid mechacrylate (HAMA). Reproduced with permission.^[^ ^598^ ^]^ Copyright 2020, Elsevier. c) Hotelling T^2^ control chart to identify printing anomalies (left) and anomaly rate contour revealing the relationship between the printing quality and the printing parameters (right). Reproduced with permission.^[^ ^93^ ^]^ Copyright 2023, Wiley. d) Overview of the image and data processing regarding the printed line analysis. Reproduced with permission.^[^ ^599^ ^]^ Copyright 2020, Elsevier.

In the realm of 3D printing, data science plays a pivotal role in enhancing the capabilities of additive manufacturing techniques, particularly in the context of bioplotting for tissue engineering. By integrating data‐driven approaches, researchers can optimize printing parameters, material selection, and scaffold design. For instance, Lee et al. employed machine learning to establish a relationship between the mechanical properties of ink and printability (**Figure** **12**a).^[^ ^597^ ^]^ The relationship facilitated the optimization of ink formulations for high printing fidelity using multiple regression analysis. Computer simulations and computational modeling allow for the virtual testing of various configurations, accelerating the development of functional and biocompatible tissue constructs. Moreover, data‐driven quality control mechanisms ensure consistent and reproducible printing outcomes. For example, Ruberu et al. use data science to enhance biomaterial processing by employing Bayesian optimization frameworks to evaluate printability and optimize material formulations (Figure 12b).^[^ ^598^ ^]^ They have applied this approach to determine the optimal concentrations of gelatin methacryloyl (GelMA) and hyaluronic acid methacrylate (HAMA), improving the quality and reproducibility of 3D bioprinting outcomes.

Similarly, Zhu et al. utilized data science in DIW for pelvic organ prolapse studies by employing a Hotelling T2 control chart to detect printing anomalies. This statistical tool identified deviations from the standard printing process, ensuring high‐quality prints. Additionally, an anomaly rate contour was used to reveal the relationship between printing quality and various printing parameters, facilitating the optimization of these parameters for improved outcomes in the fabrication of pelvic organ prolapse devices. The resultant mapping showed a trend toward optimizing the printing parameters (Figure 12c).^[^ ^93^ ^]^ Strauß et al., on the other hand, delved into the specifics of bioprinting, aiming to derive more general conclusions.^[^ ^599^ ^]^ They devised a method to discern the parameters of extruded lines, encompassing line width, length, and shrinkage behavior (Figure 12d). Their algorithm performed effectively, especially with opaque hydrogels, providing inspiration for the automated quality analysis of extrusion‐based bioprinting. These examples show that data‐driven methods are the enabler of automated quality control and process optimization in 3D printing.^[^ ^600^ ^]^ A comprehensive overview of the application of machine learning in 3D bioprinting has been compiled elsewhere.^[^ ^601^ ^]^ In general, the marriage of data science with 3D printing technologies is propelling the field forward, enabling the creation of intricate and precise structures for regenerative medicine.

While data science enhances the potential of bioplotting, it also introduces unique challenges in this complex interdisciplinary field. Bioplotting requires precise layering of bioinks to create functional tissue structures, and data‐driven optimization necessitates accurate predictive models for success. One major challenge is representing biological responses in silico, as living systems are inherently dynamic and complex. High‐quality, relevant biological data is crucial for training accurate models. Additionally, designing effective in‐situ data collection methods for high‐resolution bioplotting platforms is promising. Integrating real‐time data feedback on cellular behavior and material properties is vital for adapting and fine‐tuning processes.^[^ ^602^ ^]^ Balancing biology, material science, and engineering with data science is essential to unlock bioplotting's full potential in regenerative medicine.

## 5 Conclusions, Challenges, and Future Perspectives

### 5.1 Conclusions

Compared with conventional manufacturing techniques for polymeric biomaterials, such as injection molding, solution casting, extrusion, electrospinning, blow molding, and compression molding, 3D printing stands out due to several notable advantages. Firstly, it excels in creating customized geometries tailored to the specific needs of individual patients. This capability is particularly crucial in the medical field, where organ geometries can vary significantly among patients. By leveraging 3D printing, healthcare professionals can produce implants, prosthetics, and tissue scaffolds that precisely match the unique anatomical features of each patient, thereby enhancing treatment outcomes and patient satisfaction. Moreover, 3D printing offers the distinct advantage of minimizing material waste during the manufacturing process. Unlike traditional methods that often generate excess material that goes unused, 3D printing enables the precise deposition of material layer by layer, resulting in minimal waste and optimal resource utilization. This not only contributes to cost savings but also aligns with sustainability principles, especially considering the limited availability of tissues or implanted cells extracted from human beings. Thus, this review article comprehensively explores the integration of 3D printing technologies with biopolymers for various biomedical applications, emphasizing the transformative potential in regenerative medicine, health monitoring, and smart wearables. Additionally, 3D printing enables the creation of tunable porous structures within biomaterials. Porosity plays a critical role in various biomedical applications, including tissue engineering and pharmaceuticals. For example, porous structures are essential for facilitating vasculature development and cellular migration within engineered tissues. With 3D printing, researchers can control the porosity of biomaterials to precisely match the requirements of specific applications, enhancing the functionality and effectiveness of biomedical devices/implants.

First, the discussion spans multiple 3D printing techniques, including FDM, DIW, SLA, DLP, and SLS, highlighting their suitability for processing different biopolymers. The review covers FDM, known for its versatility and ease of use, and DIW, which excels in fabricating complex biocompatible structures. SLA and DLP are highlighted for their high‐resolution capabilities in producing intricate designs. SLS and SLM are discussed for their strengths in creating robust and durable components. Additionally, the article explores other innovative 3D printing mechanisms, emphasizing their potential to advance health applications. Then, in examining biopolymers, the article delves into both synthetic and natural macromolecules, analyzing their biocompatibility, biodegradability, and functional properties. We focus on biopolymers utilized in 3D printing from a molecular perspective, detailing both synthetic and natural variants. It provides an overview of various biopolymers, emphasizing their biocompatibility and biodegradability. The synthetic polymers discussed include PAA, PBS, PCL, PDMS, PEG, PGA, PLA, PPF, TPU, and others with limited 3D printing history. Natural macromolecules covered encompass agarose, bacterial cellulose, carrageenan, chitosan, collagen, fibrinogen, gelatin, hyaluronic acid, PHAs, silk, sodium alginate, xylan, and more. Additionally, it addresses the incorporation of human cells and tissues in 3D printing for regenerative medicine, highlighting the potential for tissue engineering and medical applications. By understanding these materials at a molecular level, researchers can optimize their use in creating functional devices, scaffolds, and implants to meet complex biomedical demands.

As a booming manufacturing tool, 3D printing is playing an increasingly important role in health applications, which require customization based on patients’ specific needs. Take the dental field as an example; it has revolutionized the fabrication of dental implants, crowns, and prosthetics with unparalleled precision and patient‐specific customization. In tissue engineering and regenerative medicine, 3D printing is used to fabricate scaffolds, tissue constructs, and organoids using bioinks composed of living cells and biocompatible materials. These constructs mimic native tissue architecture and can be implanted to promote tissue regeneration and repair. Beyond tissue scaffolds, exploring electronics, human‐machine interaction, and smart systems holds great potential for the future of healthcare. This review categorizes existing examples of 3D‐printed polymeric biomedical devices into several groups to highlight their diverse applications into the following groups:

- Sensors for health monitoring.
- Actuators for imitating biological movements.
- Wearable robotics replicating muscular motions.
- Passive energy storage systems for health purposes.
- Self‐powered devices for biomedical systems.
- Data science for bioplotting.

### 5.2 Challenges and Future Perspectives

#### 5.2.1 A Dilemma Between Printing Resolution and Manufacturing Speed

The bioprinting of biomaterials is a revolutionary technology that holds great promise for biomedical applications, including tissue engineering and regenerative medicine. However, this field is facing persistent challenges, especially when it comes to achieving the delicate balance between printing resolution and printing speed. Ideally, a bioprinter would offer both high resolution and fast speeds, but the two often exist in a tradeoff relationship. High resolution is crucial for replicating the fine, intricate structures found in human tissues, but increasing resolution often comes at the cost of speed. Conversely, prioritizing speed can compromise the precision required to build smaller, detailed biological structures. For biomedical applications, where precise replication of tissue microarchitecture is critical, finding ways to address this tradeoff is key to realizing the full potential of bioprinting in clinical settings.

One of the major limitations in bioprinting today is the inability of most commercially available 3D printers to achieve high enough resolution to replicate fine structures like human tissues. For example, the average diameter of human lung capillaries is approximately 8.3 µm,^[^ ^603^ ^]^ a size that most bioprinters struggle to replicate accurately. Although advanced technologies such as 2PP and EHD printing are capable of producing features at the nanometer scale, these printers are often prohibitively expensive, and their use in clinical settings is still quite limited.^[^ ^604^ ^]^ The bulk of bioprinting efforts, as a result, are confined to larger tissue structures, such as bone scaffolds or dermal tissue layers, where pore sizes typically range in the hundreds of microns. This limitation is not just about the technical capacity of the printers; it also affects the overall utility of bioprinting in constructing fine, detailed biological structures.^[^ ^605^ ^]^

While low resolution is a challenge, several innovative approaches have been proposed to overcome this limitation. One strategy is to combine traditional manufacturing methods with bioprinting to improve resolution without sacrificing speed. For example, Men et al. utilized salt leaching in DLP to obtain porous structures potentially useful for biocompatible applications.^[^ ^606^ ^]^ Another approach is to incorporate layer‐by‐layer assembly into the bioprinting process, as demonstrated by Jambhulkar et al., who successfully used this method to control nanofiber layers within larger‐scale structures like biosensors.^[^ ^607^ ^]^ Additionally, the same group explored the adaptability of DIW to the CLIP 3D printing platform for the directed assembly of 2D nanosheets, specifically MXene, enhancing resolution while still maintaining reasonable printing speeds.^[^ ^608^ ^]^ These strategies not only demonstrate the adaptability of existing 3D printing platforms but also point toward future developments that could make high‐resolution bioprinting more accessible for medical applications.

While resolution presents a significant challenge, low printing speed is another major bottleneck in bioprinting technology. The relatively slow nature of most bioprinting techniques means that the process can take several hours or even days to complete, making it inefficient for large‐scale production or applications where time is a critical factor. This slow speed also drives up the cost of producing printed materials, making the technology less viable for widespread use. Moreover, for applications requiring rapid prototyping, such as custom implants or bioactive drug delivery systems,^[^ ^609^, ^610^ ^]^ the slow printing rate can be a serious limitation. Conventional 3D printers are designed for speed, but bioprinting requires careful layering of cells and biomaterials to ensure viability and function, further exacerbating the issue of speed.

To address the issue of low printing speed, researchers have explored several innovations that aim to boost efficiency without compromising on precision. One of the most notable advancements in this regard is the development of CLIP technology, which significantly improves upon traditional stereolithography (SLA) by enabling continuous printing rather than the layer‐by‐layer approach that SLA typically requires.^[^ ^611^ ^]^ This continuous approach dramatically increases printing speeds without sacrificing the level of detail necessary for biomedical applications. On the other hand, a specially designed nozzle might also cut out repetitive work for more complex structures.^[^ ^37^ ^]^ Ravichandran, for instance, introduced a customized printhead for multifaceted processing, termed MDIW.^[^ ^547^ ^]^ The MDIW system incorporates multipliers that allow for the simultaneous feeding of multiple materials into distinct channels, which are then recombined to form complex structures with sub‐micron features. These printheads can reach deposition speeds comparable to traditional ink‐based 3D printing, achieving tens of centimeters per second.^[^ ^37^ ^]^ These advancements suggest that, with further refinement, it may be possible to address the speed‐resolution tradeoff and make bioprinting a more viable option for a broader range of medical applications.^[^ ^612^ ^]^

#### 5.2.2 A Mismatch Between Bioplotting Machines and Biomaterials

The biocompatibility, mechanical properties, degradation rates, and printability of biomaterials vary greatly depending on their molecular structure,^[^ ^613^ ^]^ and these factors influence how well different 3D printing techniques can process them. As listed in Table **2**, several natural polymers possess the highest biocompatibility and are only compatible with DIW printing due to their benign processing environment. For instance, synthetic polymers such as PCL and PLA are commonly used due to their excellent mechanical properties and controlled degradability.^[^ ^614^, ^615^ ^]^ However, they may not always be suitable for bioprinting processes like SLA and DLP due to the rigid requirements for photo‐curable materials and their relatively slow degradation compared to natural biopolymers. On the other hand, natural polymers like gelatin, sodium alginate, or collagen offer high biocompatibility but face challenges in achieving high resolution due to their softer nature, requiring the development of specialized bioinks.^[^ ^192^, ^296^ ^]^

Low printing resolution and slow curing times have been reported as significant challenges, particularly for polymers that do not inherently exhibit the properties required for extrusion‐based or light‐curing‐based printing technologies. Most commercially available 3D printers struggle to achieve the fine resolution needed for detailed structures (e.g., capillaries), leading to a mismatch between the polymer and the selected printing platform. For example, PAA and PGA demonstrate varying degrees of biodegradability but may have issues with thermal extrusion, resulting in suboptimal print quality.^[^ ^616^, ^617^ ^]^ Similarly, PDMS is widely used in biomedical applications due to its excellent biocompatibility and flexibility, but its low resolution in extrusion‐based 3D printing limits its ability to form intricate structures necessary for organ scaffolds.^[^ ^229^ ^]^ Moreover, gelatin‐based hydrogels, which are commonly used due to their cell compatibility, face rapid degradation during light‐based printing due to their sensitivity to UV exposure, compromising the mechanical properties of the final structure.^[^ ^376^, ^377^, ^378^ ^]^ PEG offers tunable mechanical and degradation properties, but it often exhibits poor print fidelity at smaller scales.^[^ ^618^ ^]^ Additionally, natural polymers like sodium alginate and collagen,^[^ ^619^, ^620^ ^]^ which offer high biocompatibility, struggle to maintain structural integrity during the printing process due to their low viscosity, requiring post‐processing steps to enhance resolution and stability. Therefore, achieving a balance between print fidelity, mechanical strength, and biocompatibility continues to be a key challenge in the bioprinting of complex tissue structures​.

Various strategies can be adopted to optimize the compatibility of biomaterials with specific 3D printing platforms. For example, for SLA or DLP printing, photopolymerizable bioinks such as GelMA have shown promise due to their tunable mechanical properties and photo‐curability.^[^ ^376^, ^377^, ^378^ ^]^ However, these materials need to be functionalized further to meet the specific requirements of each application. Near‐infrared (NIR) light curing, as an alternative to UV light curing, has been shown to reduce the damage to cells or bioactive molecules during the bioprinting process, thereby enhancing the preservation of cell viability.^[^ ^621^, ^622^ ^]^ However, recalibrating printing parameters is necessary when switching to the new light source. Moreover, the use of shear‐thinning materials, particularly in DIW, can reduce cell damage caused by shear stress, but this requires careful optimization of bioink viscosity and printhead configurations.^[^ ^623^ ^]^ In this case, customization or optimization of current 3D printing techniques is needed to facilitate the utilization of biocomponents without sacrificing printing quality too much.^[^ ^93^ ^]^ Another area of optimization involves combining biopolymers with reinforcing nanomaterials such as carbon nanotubes or graphene. These materials can significantly enhance mechanical properties and improve the printability of biomaterials across platforms like DIW or FDM. Recent studies have also explored co‐extrusion techniques, where biomaterials with differing mechanical properties are printed simultaneously, allowing for more complex, layered tissue scaffolds with varying degradation rates and functions.^[^ ^624^ ^]^

Emerging strategies to solve the mismatch between biomaterials and 3D printing technologies involve the customization of printers themselves. By developing specialized printheads or multiphase print systems, such as MDIW,^[^ ^547^ ^]^ researchers can combine various materials into a single scaffold, optimizing for both mechanical strength and biological function. MDIW systems allow for the simultaneous extrusion of bioactive and structural materials while achieving submicron resolutions without sacrificing printing speed.^[^ ^37^ ^]^ Additionally, hybrid manufacturing systems that combine techniques like salt‐leaching or freeze‐drying with 3D printing can also address resolution issues in soft materials by providing additional structural support during printing.^[^ ^606^ ^]^ By addressing these challenges with customized approaches to material compatibility and printer design, researchers can greatly enhance the capabilities of 3D‐printed biomedical devices, enabling finer, more functional tissue structures while ensuring that cells and biomaterials retain their properties during and after printing.

#### 5.2.3 A Material Limit for Health Potential—Biocompatibility and Biodegradability

Biocompatibility and biodegradability are two critical factors in designing materials for medical implants, tissue engineering scaffolds, drug delivery systems, and transient medical devices.^[^ ^625^ ^]^ Biocompatibility ensures that the material will not elicit a harmful immune response when introduced into the body, while biodegradability refers to how the material will break down over time and be safely absorbed or excreted by the body. In tissue engineering, for instance, balancing these two properties becomes crucial. Scaffolds for tissue repair need to provide a temporary matrix for cell growth, but the material should degrade at a controlled rate as the tissue regenerates, to avoid the need for secondary surgeries.^[^ ^626^ ^]^ If the material degrades too quickly, it may lose mechanical support before the tissue fully regenerates. Conversely, if it degrades too slowly, it may cause chronic inflammation or fibrosis, undermining the healing process.

Another example is drug delivery systems, where the rate of degradation directly influences drug release kinetics. A biodegradable polymer used in a drug delivery device must degrade at a predictable rate to ensure a consistent and sustained release of the therapeutic agent.^[^ ^627^ ^]^ Materials such as PLGA have been extensively studied for this purpose, as their degradation rate can be tailored by adjusting the ratio of lactic acid to glycolic acid.^[^ ^240^ ^]^ However, while PLGA offers excellent biodegradability and biocompatibility, its mechanical properties may not be ideal for load‐bearing applications, indicating the need to balance material strength with degradation profiles.

One significant challenge arises in the use of functional fillers (as described in **Chapter 4**), such as nanoparticles or conductive polymers, within biodegradable materials. While these fillers can enhance the material's functionality, such as electrical conductivity for biosensors or piezoelectric properties for actuators, their degradation, and long‐term biocompatibility remain poorly understood. For instance, studies on the incorporation of carbon‐based nanoparticles like graphene or carbon nanotubes into biodegradable polymers show promise for enhancing conductivity, but their persistence in the body and potential toxicity as they degrade remains a concern.^[^ ^489^, ^628^ ^]^ Degradable conductive polymers like polyaniline and poly(3,4‐ethylenedioxythiophene) have shown potential, particularly in applications like nerve regeneration,^[^ ^629^ ^]^ but balancing their functionality with predictable degradation rates is still a challenge.

For implants, such as bone scaffolds or soft tissue prosthetics, it is crucial that the material maintains its mechanical integrity for the required duration before gradually degrading.^[^ ^630^ ^]^ Materials such as PCL and PLA are widely used for bone and tissue scaffolds due to their slow degradation rates, making them suitable for long‐term support.^[^ ^631^, ^632^ ^]^ However, tuning their degradation profile to match the rate of tissue regeneration remains a complex task, especially when incorporating bioactive molecules or cells into the matrix. For example, collagen‐based scaffolds used for wound healing offer excellent biocompatibility and moderate degradation rates,^[^ ^633^ ^]^ but their mechanical properties are often insufficient for more load‐bearing applications like cartilage or bone repair.

To address these challenges, future research may explore the functionalization of biopolymers to integrate biodegradability with additional properties, such as electrical conductivity, mechanical strength, or bioactivity. Advances in 3D bioprinting offer the potential for precise control over material deposition, enabling the creation of multi‐material structures that combine biocompatibility, biodegradability, and other functional properties. For example, degradable conjugated polymers might be a candidate to integrate degradation and function since it has been studied for cancer therapy,^[^ ^629^ ^]^ and collagen itself has been reported to possess a piezoelectric effect for years.^[^ ^362^ ^]^ By strategically designing scaffolds with spatially controlled degradation rates, researchers can enhance tissue regeneration while maintaining functionality, as well as the integrity of biosensors/actuators/health devices throughout the healing process.^[^ ^634^ ^]^ In conclusion, balancing biocompatibility and biodegradability with other material properties is a complex task that requires careful consideration of the specific application and the material's role in the body. Ongoing advancements in materials science, particularly in the field of 3D bioprinting, will likely provide new opportunities to develop multifunctional materials that address these challenges. By integrating biodegradable polymers with functional fillers, tuning degradation rates, and leveraging bioactive molecules, it will be possible to create advanced biomedical devices that meet the diverse needs of tissue engineering, drug delivery, and medical implants.

#### 5.2.4 Practical Challenges of Biomaterial Bioprinting—Regulatory Requirements and Ethical Issues

Recent advances in the clinical application of 3D‐printed parts have made significant strides in various medical fields, with numerous studies reporting successful outcomes. For instance, 3D‐printed patient‐specific implants have been validated in orthopedic surgery,^[^ ^635^ ^]^ where they are used to repair bone defects or replace joints, ensuring a better fit and quicker recovery compared to traditional methods. In the field of cardiovascular medicine, 3D‐printed models of complex heart structures are used to plan surgeries, improving precision and reducing operating times.^[^ ^636^ ^]^ Furthermore, clinical trials for 3D‐printed skin grafts and scaffolds are also underway, showing promising results in treating burn victims by promoting faster and more efficient tissue regeneration.^[^ ^637^ ^]^ Additionally, recent FDA‐approved 3D‐printed surgical guides for dental implants have demonstrated significant improvements in both accuracy and patient outcomes.^[^ ^638^ ^]^ These cases exemplify the growing importance of 3D printing in developing tailored solutions to address patient‐specific challenges, showcasing the practical utility and potential for broader clinical adoption in the near future.

One of the primary hurdles is navigating the complex regulatory landscape.^[^ ^639^ ^]^ For instance, 3D‐printed biomaterials, especially those intended for clinical use, must meet stringent safety and efficacy standards set by regulatory bodies such as the U.S. Food and Drug Administration (FDA) and the European Medicines Agency (EMA). The classification of 3D‐printed devices as medical devices or combination products (those that include both drugs and devices) adds layers of regulatory oversight.^[^ ^640^ ^]^ In particular, for combination products, the evaluation process must ensure that both the device component and the biologically active materials (e.g., cells and drugs) meet the necessary safety profiles. Additionally, manufacturers must demonstrate consistent performance in biocompatibility, degradation profiles, and mechanical integrity through extensive preclinical studies and, eventually, clinical trials. For example, while the use of biocompatible polymers such as PLA and PCL is well‐established, combining them with bioactive molecules,^[^ ^641^ ^]^ or even cells, raises new regulatory challenges that could complicate the approval process.

From an ethical standpoint, the development and application of 3D‐printed biomaterials also pose significant concerns. One challenge involves patient‐specific treatments, where 3D‐printed scaffolds or implants are customized for individual patients based on their anatomical data, such as CT or MRI scans.^[^ ^641^ ^]^ While this presents a revolutionary approach to personalized medicine, it also raises ethical questions about data privacy and the ownership of personal medical data used to create these models.^[^ ^642^ ^]^ Moreover, the use of human‐derived cells in bioprinting, particularly for organoid or tissue engineering applications, requires a deep understanding of ethical frameworks surrounding consent, cell sourcing, and the long‐term impact of using such materials. As the field progresses, it is essential to incorporate more detailed discussions on how ethical considerations, such as patient autonomy and the accessibility of these cutting‐edge treatments, will impact the future of 3D‐printed biomaterials in clinical settings.^[^ ^643^ ^]^ These challenges underscore the importance of developing guidelines that address both the regulatory and ethical dimensions of 3D‐printed biomaterials.

## Conflict of Interest

The authors declare no conflict of interest.
